# Correction: Ketogenic diet improves disease activity and cardiovascular risk in psoriatic arthritis: A proof of concept study

**DOI:** 10.1371/journal.pone.0344504

**Published:** 2026-03-06

**Authors:** Roberta Ramonda, Francesca Ometto, Giovanni Striani, Giacomo Cozzi, Daniela Basso, Filippo Evangelista, Mariagrazia Lorenzin, Laura Scagnellato, Ada Aita, Marta Favero, Filippo Brocadello, Andrea Doria

There are errors in references 1, 7, 9, 22, 23, 24 and 25. The correct references are reported below:

**1.** Kris-Etherton PM, Harris WS, Appel LJ. Omega-3 fatty acids and cardiovascular disease: New recommendations from the American Heart Association. Arterioscler Thromb Vasc Biol. 2003;23(2):151-2.

**7.** Ford A, Siegel M, Bagel J, Cordoro K, Garg A. Dietary recommendations for adults with psoriasis or psoriatic arthritis from the Medical Board of the National Psoriasis Foundation: A systematic review. JAMA Dermatol. 2018;154(8):934-950.

**9.** Ometto F, Ortolan A, Farber D, Lorenzin M, Dellamaria G, Cozzi G, et al. Mediterranean diet in axial spondyloarthritis: an observational study in an Italian monocentric cohort. Arthritis Res Ther. 2021;23(1):219.

**22.** Harris A, Hinman RS, Lawford BJ, Egerton T, Keating C, Brown C, et al. Cost-effectiveness of telehealth-delivered exercise and dietary weight loss programs for knee osteoarthritis within a twelve-month randomized trial. Arthritis Care Res. 2023;75(6):1311-1319.

**23.** Castaldo G, Marino C, Atteno M, D’Elia M, Pagano I, Grimaldi M, et al. Investigating the effectiveness of a carb-free oloproteic diet in fibromyalgia treatment. Nutrients. 2024;16(11):1620.

**24.** Castaldo G, Rastrelli L, Galdo G, Molettieri P, Rotondi Aufiero F, Cereda E. Aggressive weight-loss program with a ketogenic induction phase for the treatment of chronic plaque psoriasis: A proof-of-concept, single-arm, open-label clinical trial. Nutrition. 2020:74:110757.

**25.** Barrea L, Caprio M, Camajani E, Verde L, Elce A, Frias-Toral E, et al. Clinical and nutritional management of very-low-calorie ketogenic diet (VLCKD) in patients with psoriasis and obesity: a practical guide for the nutritionist. Crit Rev Food Sci Nutr. 2023;63(31):10775-91.
