# Supplementary material for: Ketogenic diet improves disease activity and cardiovascular risk in psoriatic arthritis: A proof of concept study
Source: PLoS One. 2025 Apr 22;20(4):e0321140. doi: 10.1371/journal.pone.0321140 (PMC12013891; doi:10.1371/journal.pone.0321140)
Supplement: S1 Table — (PDF) [file pone.0321140.s001.pdf]

Table S1. Full list of variables included in the study.

**Sociodemographic variables**	
	Gender
	Age
	Smoke ever or current
	Education (higher: secondary school or graduation)
	Employment
**Anthropometric measures**	
	Height, cm
	Weight, kg
	Height, cm
	BMI, kg/m^2
	Abdominal circumference, cm
**Disease history**	
	Instrumental analysis, number/year,
	Distance from the prescription centre, km
	Disease duration, years
	Psoriasis duration, years
	Arthritis onset (first ever instances): oligoarticular; poliarticular; enthesopatic
	Psoriasis severity: mild (only topical therapy with good results); moderate-severe (phototherapy or systemic therapy)
	Clinical domains: onychopathy; anterior uveitis (diagnosis based on eye examination); ibd; peripheral arthritis; dactylitis; enthesitis; spondylitis; dip arthritis; tenosynovitis
	Peripheral radiological damage
	Axial radiological damage
	Sacroiliitis according to the NY criteria
	RF and/or ACPA
	HLA-B27
	Previous therapies: csDMARDs; tsDMARDs; bDMARDs; number of bDMARDs or tsDMARDs classes
	Current treatment: current csDMARD; current b/tsDMARD; TNF inhibitors; IL-23 inhibitors; IL-17 inhibitors; therapeutic combination; ongoing steroids
**Clinical variables**	
	Tender joints count (0-68)
	Swollen joints count (0-68)
	Tender joints count (0-28)
	Swollen joints count (0-28)
	Dactylitis
	DAPSA
	DAS28-CRP
	CDAI
	SDAI
	BASDAI
	ASDAS-CRP
	SPARCC
	LEI
	BSA
	PASI
	MDA
**Patient- and physician-reported outcomes**	
	PASS
	HAQ
	PtGA (0-10 cm)
	PGA (0-10 cm)
	VAS pain (0-10 cm)
	CQR5 High Adherers
	WPAI: Weekly working hours; lost work hours; impact
**Inflammatory biomarkers**	
	hsCRP, mg/L
	ESR, mm/h
	IL-1α, ng/L
	IL-1β, ng/L
	IL-6, ng/L
	TNFα, ng/L
	Fecal calprotectin μg/g,

Laboratory variables

Lipid profile: total cholesterol, mg/dl; HDL cholesterol, mg/dl
LDL cholesterol, mg/dl; Triglyceride, mg/dl
AST, U/L
ALT, U/L
GGT, U/L
TSH, mU/L
Creatinine, mg/dl
Uricemia, mmol/L
Blood glucose, mg/dl
Insulinemia, mU/L
HOMA-IR index
Azotemia, mmol/L
Blood count: WBC, 10^9 /L; RBC, 10^{12} /L; Hb, g/L; Hc, L/L; MCV, fL; MCH, g/L; MCHC, g/L; RDW, %; platelet, 10^9 /L; neutrophils, 10^9 /L; lymphocytes, 10^9 /L; monocytes, 10^9 /L; eosinophilic, 10^9 /L; basophil, 10^9 /L
Protein profile: total proteins, g/L; albumin; α1-globulin; α2-globulin; β1-globulin; β2-globulin; γ-globulin
Urine test: pH; glucose, mmol/L; protein, g/L; hb, g/L; ketones, g/L; bilirubin, μmol/L; urobilinogen, μmol/L; specific weight
Intestinal permeability test:
diuresis 6h, ml; lactulose, %; mannitol, %; sucrose, %; lactulose/mannitol ratio

Nutritional questionnaires

Food Frequency Questionnaire: cereals and derivatives; processed cereal products; fresh meat; processed meat; seafood ; milk and yoghurt; dairy products; fresh fruit; nuts; vegetables; legumes; eggs; sweets; soda; alcoholic beverages; weekly physical activity
PREDIMED (PREvención con DIeta MEDiterránea) score

Cardiovascular parameters

SBP, systolic blood pressure
DBP, diastolic blood pressure
CUORE, 10 year risk of cardiovascular events according to the Progetto CUORE estimator
SCORE2, Systematic Coronary Risk Evaluation 2 estimator

CQR5, 5-item compliance questionnaire for rheumatology; COPD, chronic obstructive pulmonary disease; MI, acute myocardial infarction; IBD, inflammatory bowel disease; DIP, distal interphalangeal joints; NY, New York criteria for sacroiliitis; RF, rheumatoid factor; ACPAs, anti-citrullinated protein antibodies; HLA, human leukocyte antigen; csDMARDs conventional synthetic disease modifying antirheumatic drugs; b/tsDMARDs biological/targeted synthetic disease-modifying antirheumatic drugs; NSAIDs, non-steroidal anti-inflammatory drugs; TNF-α, tumor necrosis factor α; IL, interleukin.
BMI, body mass index.
DAPSA, disease activity index in psoriatic arthritis; DAS28-CRP, disease activity score on 28 joints based on C reactive protein; CDAI, clinical disease activity index; SDAI, Simple Disease Activity Index; BASDAI, Bath Ankylosing Spondylitis Disease Activity Index; ASDAS-CRP, Ankylosing Spondylitis Disease Activity Score based on CRP; SPARCC, Spondylarthritis Research Consortium of Canada; LEI, Leeds Enthesitis Index; BSA, Body Surface Area; PASI, Psoriasis Area Severity Index; MDA, Minimal Disease Activity; PASS, Patient Acceptable Symptom State; HAQ, Health Assessment Questionnaire; PtGA, patient global assessment; PGA, Physician Global Assessment; VAS, Visual Analogue Scale; WPAI, Work Productivity and Activity Impairment questionnaire.
hsCRP, High Sensitivity C Reactive Protein, ESR, Erythrocyte Sedimentation Rate; IL, interleukin; TNFα, Tumor Necrosis Factor alpha; HDL, High Density Lipoprotein; LDL, Low Density Lipoprotein; ALT, alanine aminotransferase; AST, aspartate aminotransferase; GGT, gamma glutamyl transpeptidase; TSH, thyroid-stimulating hormone; HOMA-IR, Homeostatic Model Assessment for Insulin Resistance; WBC, white blood cells; RBC, red blood cells; Hb; hemoglobin; MCV, mean corpuscular volume; MCH, mean corpuscular hemoglobin; MCHC, mean corpuscular hemoglobin concentration; RDW, red cell distribution width; PREDIMED (PREvención con DIeta MEDiterránea) score; SBP, systolic blood pressure; DBP, diastolic blood pressure; CUORE, 10 year risk of cardiovascular events according to the Progetto CUORE estimator; SCORE2, Systematic Coronary Risk Evaluation 2 estimator.
