# Supplementary material for: Ketogenic diet improves disease activity and cardiovascular risk in psoriatic arthritis: A proof of concept study
Source: PLoS One. 2025 Apr 22;20(4):e0321140. doi: 10.1371/journal.pone.0321140 (PMC12013891; doi:10.1371/journal.pone.0321140)
Supplement: S2 Table — (PDF) [file pone.0321140.s002.pdf]

Table S2. Characteristics of the patients at W0.

Subjects	20
Male, n (%)	9 (45.0)
Age, years, median (IQR)	55.5 (50;59.3)
Height, cm, median (IQR)	173 (164;177.3)
Smoke ever, n (%)	6 (30.0)
Higher education, n (%)	13 (65.0)
Employed, n (%)	16 (80.0)
Instrumental analysis/year, median (IQR)	1 (1;2)
Distance in km from the prescription centre, median (IQR)	15 (6;32.5)
CQR5 High Adherers, n (%)	15 (75.0)
Comorbidities	
Metabolic syndrome, n (%)	3 (15.0)
Cardiovascular comorbidity, n (%)	7 (35.0)
Asthma/COPD, n (%)	0 (0)
MI o stroke o other cardiovascular events, n (%)	0 (0)
Arterial hypertension, n (%)	7 (35.0)
Fibromyalgia, n (%)	2 (10.0)
Ulcer or other gastrointestinal diseases, n (%)	5 (25.0)
Type 2 diabetes, n (%)	2 (10.0)
Fractures, n (%)	2 (10.0)
Depression, n (%)	1 (5.0)
Solid tumor, n (%)	3 (15.0)
Hematological tumor , n (%)	0 (0)
Disease duration, years, median (IQR)	8.8 (4.2;14.3)
Psoriasis duration, years, median (IQR)	23.4 (9.8;122.7)
Arthritis onset (first ever instances)	
Oligoarticular, n (%)	7 (35.0)
Poliarticular, n (%)	11 (55.0)
Enthesopatic, n (%)	2 (10.0)
Psoriasis severity	
Mild (only topical therapy with good results), n (%)	14 (70.0)
Moderate-severe (phototherapy or systemic therapy), n (%)	6 (30.0)
Clinical domains	
Onychopathy, n (%)	10 (50.0)
Anterior uveitis (diagnosis based on eye examination), n (%)	0 (0.0)
IBD, n (%)	0 (0.0)
Peripheral arthritis, n (%)	12 (60.0)
Dactylitis, n (%)	4 (20.0)
Enthesitis, n (%)	14 (70.0)
Spondylitis, n (%)	8 (40.0)
DIP arthritis, n (%)	13 (65.0)
Tenosynovitis, n (%)	11 (55.0)
Peripheral radiological damage, n (%)	7 (35.0)
Axial radiological damage, n (%)	10 (50.0)
Sacroiliitis according to the NY criteria, n (%)	6 (30.0)
RF and/or ACPA, n (%)	1 (5.0)
HLA-B27, n (%)	1 (5.0)
Previous therapies	
csDMARDs, n (%)	19 (95.0)
tsDMARDs, n (%)	0 (0.0)
bDMARDs, n (%)	13 (65.0)
Number of bDMARDs or tsDMARDs classes, median (IQR)	1 (0;1)
Current treatment	
Current csDMARD, n (%)	6 (30.0)
Current b/tsDMARD, n (%)	14 (70.0)
TNF inhibitors, n (%)	9 (45.0)
IL-23 inhibitors, n (%)	1 (5.0)
IL-17 inhibitors, n (%)	3 (15.0)
Therapeutic combination, n (%)	6 (30.0)
Ongoing steroids, n (%)	1 (5.0)

Categorical variables are reported as number and percentage, continuous variables are reported as median and interquartile range.

IQR, interquartile range; CQR5, 5-item compliance questionnaire for rheumatology; COPD, chronic obstructive pulmonary disease; MI, acute myocardial infarction; IBD, inflammatory bowel disease; DIP, distal interphalangeal joints; NY, New York criteria for sacroiliitis; RF, rheumatoid factor; ACPAs, anti-citrullinated protein antibodies; HLA, human leukocyte antigen; csDMARDs conventional synthetic disease modifying antirheumatic drugs; b/tsDMARDs biological/targeted synthetic disease-modifying antirheumatic drugs; NSAIDs, non-steroidal anti-inflammatory drugs; TNF-α, tumor necrosis factor α; IL, interleukin.
