# Supplementary material for: Ketogenic diet improves disease activity and cardiovascular risk in psoriatic arthritis: A proof of concept study
Source: PLoS One. 2025 Apr 22;20(4):e0321140. doi: 10.1371/journal.pone.0321140 (PMC12013891; doi:10.1371/journal.pone.0321140)
Supplement: S3 Table — (PDF) [file pone.0321140.s003.pdf]

Table S3a. Association between continuous and categorical variables at W0 (part 1).

		W0 Age	DD Arthritis	DD Psoriasis	W0 Weight	W0 BMI	W0 Abdominal circumference	W0 SBP	W0 DBP	W0 SCORE2¤^	W0 CUORE§
**Gender**	**1**	52 (47;60)	11.8 (5.3;18.8)	21.7 (15.8;41.5)	97.2 (90.3;107.3)	32.4 (28.8;34.3)	115 (105;124)	145 (130;150)	90 (85;95)	15 (5.9;18)	2 (2;4)
	**0**	56 (52;58.5)	5.3 (3.1;10.1)	42.3 (7.7;122.7)	84.1 (79.9;93)	30.8 (29.5;32.1)	106 (102.5;106.5)	130 (110;140)	80 (67.5;80)	6.4 (3;7.7)	1 (1;1)
**Smoke ever**	**1**	59 (50;59.8)	8.5 (5.1;13.3)	34.4 (18.1;103)	101.4 (91.2;106.9)	32.4 (30.4;33.8)	115.5 (105.3;122)	145 (132.5;150)	85 (80;93.8)	15 (11.3;18)	4 (2;4)
	**0**	54 (50.3;57.8)	8.8 (3.6;17.1)	20.3 (7.8;102.6)	86.6 (80.6;96.1)	30.3 (28.9;32.5)	106 (104.3;107.8)	132.5 (105;140)	80 (67.5;88.8)	6 (3.2;7.7)	1 (1;1.8)
**Higher education**	**1**	52 (47;56)	5.3 (2.9;18.8)	41.5 (18.8;122.7)	90.3 (82.8;105.5)	30.8 (28.7;33)	105 (103;115)	135 (120;140)	80 (70;85)	6.2 (3;13.4)	1 (1;2)
	**0**	59 (57.5;65)	11.5 (7.1;11.8)	11.5 (8.8;29.8)	91.7 (79.9;97)	31.2 (29.7;32.7)	107 (106;112)	140 (125;150)	90 (77.5;90)	6.9 (6.4;11.5)	2 (1;3)
**Employed**	**1**	52.5 (49.3;57.3)	5.3 (3.2;14.3)	23.4 (6.2;122.7)	92.3 (82.7;106)	30.9 (29.3;33.3)	106.5 (102.8;117.3)	137.5 (127.5;145)	80 (77.5;90)	6.2 (3.3;12.1)	1 (1;2)
	**0**	65 (65;66.8)	11.6 (10.8;15.5)	26.5 (11.3;42.1)	86.1 (80.1;93.1)	30.2 (29;31.5)	106 (105.8;108.5)	130 (90;145)	80 (56.3;88.8)	15.2 (7.7;23.7)	2.5 (1;4.5)
**bDMARDs**	**1**	53 (48.5;58)	5 (2.8;14.8)	18.8 (3.7;122.7)	94.2 (83.5;106.4)	32.4 (30.3;33.6)	107 (102.5;119.5)	140 (125;142.5)	80 (75;90)	7.3 (3.3;14.1)	1.5 (1;2)
	**0**	58 (51;65)	11.5 (8.7;11.8)	41.5 (11.5;43.8)	90.3 (79;96.7)	29.5 (28.8;31.2)	106 (105;108)	135 (120;145)	85 (75;90)	6.9 (5.9;12.9)	1 (1;2)
**Axial involvement**	**1**	56.5 (52.3;61.3)	7 (4.9;12)	33.3 (9.2;122.7)	83.5 (80.1;95.1)	30.9 (29.1;32.5)	106 (103.8;107.8)	130 (115;140)	80 (68.8;86.3)	6.9 (4.6;10.7)	1 (1;2)
	**0**	51.5 (48.8;59)	11.8 (3.2;16.6)	20.3 (13.5;42.7)	97 (90;113.9)	31.1 (29.3;35.9)	112 (104.3;125.3)	142.5 (133.8;146.3)	87.5 (80;91.3)	8.6 (3.9;15)	1.5 (1;2.5)
**Comorbidities**	**1**	57 (52.5;62)	8.8 (5.3;17.3)	43.8 (17.3;122.7)	82.2 (79.8;97.5)	30.8 (28.9;32.4)	106 (103.5;113)	140 (130;147.5)	85 (80;90)	8.4 (6.4;15)	2 (1;3)
	**0**	51 (45;59)	5 (2.9;11.8)	11.5 (2.9;25)	94.2 (90.3;105.5)	31.2 (29.5;34.3)	106 (105;115)	130 (0;140)	80 (0;85)	5 (3.5;8.8)	1 (1;1.3)
**Metabolic syndrome**	**1**	57.5 (55.3;63.8)	7 (4.6;11.7)	33.3 (8;102.9)	93 (80.6;97.1)	31 (29.6;32.8)	106.5 (105.3;109.5)	135 (107.5;147.5)	82.5 (68.8;90)	7.3 (5.9;16.7)	1.5 (1;3.5)
	**0**	50.5 (45.5;57.3)	11.3 (3.8;18.1)	20.3 (12.1;102.6)	89.8 (83.1;109.9)	30.4 (28.9;34.6)	105.5 (102.3;124.8)	137.5 (122.5;143.8)	80 (76.3;88.8)	6.9 (3.6;11.3)	1 (1;2)
**Cardiovascular comorbidities**	**1**	57 (51;58.5)	11.8 (5.3;17.3)	43.8 (17.3;122.7)	82.8 (81.5;102.3)	32.4 (30.4;32.7)	107 (101;120)	145 (140;150)	90 (85;92.5)	8.4 (6.4;15)	2 (1.5;3)
	**0**	55 (50;60)	8.7 (2.9;11.8)	21.7 (6.8;42.3)	91.7 (84.1;97.7)	29.8 (28.8;33)	106 (105;110)	130 (100;140)	80 (65;85)	6.4 (3.5;11.7)	1 (1;1.3)
**W0 elevated IL-1β**	**1**	59.5 (57.5;61.3)	8.3 (4.1;12.5)	20.4 (14.7;49.5)	101.6 (96.2;106.8)	32.7 (31.1;34.6)	112.5 (109;117.5)	140 (97.5;150)	85 (60;91.3)	11.5 (6.7;15.8)	2.5 (1;4)
	**0**	52 (48.5;57.5)	8.7 (3.9;16.3)	41.5 (5.7;122.7)	89.2 (81.5;97)	29.8 (28.8;32.7)	105 (102.5;112)	140 (125;142.5)	80 (75;90)	6.4 (3.7;12.5)	1 (1;2)
**W0 elevated IL-6**	**1**	55 (46.3;59)	8.6 (3.2;14.3)	29.1 (12;62.5)	100 (87.9;120.1)	32.6 (28.8;40.3)	113.5 (99.3;130)	137.5 (131.3;142.5)	80 (77.5;82.5)	11.3 (6.8;13.1)	1 (1;2.5)
	**0**	55 (50;62.5)	8.8 (4.8;15.3)	21.7 (8.8;83.2)	91.7 (81.5;97.5)	31 (29;32.7)	106 (105;112.5)	130 (110;142.5)	80 (70;90)	6.9 (3.8;14)	1 (1;2)
**W0 elevated fecal calprotectin**	**1**	58 (53.5;65)	11.5 (7;16.2)	42.3 (31.6;83.2)	90.3 (78.9;94.5)	29.8 (28.8;31.8)	105 (104.5;111)	140 (117.5;147.5)	85 (72.5;87.5)	8 (5.1;17.7)	1 (1;2.5)
	**0**	53 (50;59)	5.3 (2.9;13.8)	15.8 (4.5;122.7)	94.2 (82.8;105.5)	31 (29.5;33)	107 (103;115)	130 (120;140)	80 (75;90)	6.5 (3.8;12.2)	1.5 (1;2)
**W0 MDA**	**1**	52.5 (46.3;57.5)	11.8 (6.2;17.6)	31.6 (9.8;102.9)	87.2 (80.9;97.1)	29.2 (28.6;30.6)	105 (100.3;109.5)	135 (122.5;145)	85 (72.5;90)	6.6 (4.1;15)	1 (1;2)
	**0**	58 (52;59.8)	5.2 (3.6;10.8)	20.4 (11;102.6)	93 (84.4;106.9)	32.7 (31;34)	106.5 (106;121.8)	137.5 (122.5;140)	80 (76.3;87.5)	7.4 (3.8;10.5)	1 (1;2)
**W0 PASS**	**1**	52 (45;58)	11.8 (8.7;18.8)	21.7 (6.8;43.8)	84.1 (80.5;96.7)	28.8 (28.5;29.8)	105 (99;108)	140 (120;145)	85 (70;90)	9.8 (5.4;16.9)	1.5 (1;2.5)
	**0**	57 (52;59.5)	5 (3.1;10.1)	25 (11.1;122.7)	94.2 (86;106.4)	32.4 (30.9;33.6)	107 (106;119.5)	135 (125;140)	80 (77.5;85)	6.9 (3;9.8)	1 (1;2)
**W0 physical activity°**	**1**	56.5 (52.8;58.8)	8.8 (4.7;13.3)	20.3 (6.1;102.9)	83.2 (79.4;90)	29.5 (28.7;31.8)	104.5 (99.8;106)	140 (120;145)	80 (71.3;88.8)	6.9 (6.2;12.9)	1 (1;2)
	**0**	52 (50;59.8)	8.4 (3.8;14.8)	33.3 (12.5;102.6)	101.6 (93;109.9)	31.8 (30.9;35.1)	112.5 (106.5;124.8)	132.5 (130;140)	82.5 (80;90)	6.9 (3.3;13.4)	1.5 (1;3.5)

Gender “1” refers to male, “0” refers to female; for the other variables “1” refers to “yes”, “0” refers to “no”. Data are reported as median and interquartile range.

Significant associations are indicated by green cells. Significance refers to the Kruskal-Wallis test.

° Weekly, according to the Food Frequency Questionnaire.

¤ Computed from 19 subjects.

§ 10 year risk of cardiovascular events according to the Progetto CUORE estimator. SCORE2-OP (Older People) estimator was used for subjects >70 years. Values were adjusted for subjects with inflammatory arthritis. Probability is expressed as percentage of risk. ^ 10 year risk of cardiovascular events according to the ESC (European Society of Cardiology), SCORE2 (Systematic Coronary Risk Evaluation 2) estimator. Values were adjusted for subjects with inflammatory arthritis. Probability is expressed as percentage of risk.

The subsequent baseline variables were excluded from the analysis of the study group due to inadequate case number: elevated IL-1α, fibromyalgia, uveitis, inflammatory bowel disease, HLA-B27.

W0, week 0; DD, disease duration; BMI, body mass index; SBP, systolic blood pressure; DBP, diastolic blood pressure; CUORE, cardiovascular unique offer reengineered; SCORE2, systematic coronary risk evaluation; bDMARDs, biological disease-modifying antirheumatic drugs; IL, interleukin; MDA, Minimal Disease Activity; PASS, Patient Acceptable Symptom State.

Table S3b. Association between continuous and categorical variables at W0 (part 2).

		W0 DAPSA	W0 DAS28-CRP	W0 PASI	W0 BSA	W0 ASDAS-CRP	W0 BASDAI	W0 LEI	W0 SPARCC	W0 VAS pain	W0 PGA	W0 PtGA	W0 WPAI lost work hours	W0 PREDIMED
**Gender**	**1**	8 (3;17)	2.5 (1.9;3.5)	0.5 (0;3)	1 (0;1)	0.4 (0.2;1.5)	1 (0.7;3.3)	0 (0;1)	0 (0;2)	1 (0;4)	2 (1;3)	2 (1;4)	0 (0;0)	7 (6;9)
	**0**	12.1 (9.5;26.1)	2.9 (2.3;3.8)	0 (0;0.5)	0 (0;0.5)	1.5 (1.2;2)	3.7 (2.7;4.5)	0 (0;4)	3 (0;7)	5 (3;7)	4 (3.5;5.5)	4 (3.5;6.5)	0 (0;4)	7 (7;9.5)
**Smoke ever**	**1**	13.6 (8.6;18.8)	2.6 (1.9;3.3)	0.3 (0;1.5)	0.5 (0;1)	1.5 (1.4;1.5)	3.5 (3.3;4.3)	0 (0;0.8)	0 (0;0.8)	5 (4;6.8)	4.5 (3.3;5.8)	4.5 (4;5.8)	0 (0;0)	7 (6.3;7.8)
	**0**	11.5 (5.2;28.1)	2.7 (2.2;4)	0 (0;1.6)	0 (0;1)	0.8 (0.3;1.8)	1.8 (0.8;4.3)	1 (0;4)	2.5 (0;6)	2 (1.3;5)	2.5 (1.3;4.8)	2.5 (1.3;4.8)	0 (0;0)	7.5 (7;9.8)
**Higher education**	**1**	11 (8;19.3)	2.5 (2.2;3.5)	0.4 (0;1.8)	0 (0;1)	1.4 (0.7;1.5)	3.3 (1.7;3.7)	0 (0;1)	1 (0;3)	4 (2;5)	3 (2;5)	4 (2;5)	0 (0;0)	7 (7;9)
	**0**	12.1 (4.1;25.5)	2.9 (1.4;3.8)	0 (0;0.9)	0 (0;0.5)	1.6 (0.5;2.3)	3.8 (1.1;5.5)	4 (0;5)	6 (0;7)	5 (1.5;7.5)	4 (2;7)	4 (2;7)	0 (0;5.3)	8 (7;9.5)
**Employed**	**1**	11.5 (7.3;29.3)	2.6 (2.2;3.7)	0.2 (0;1.8)	0 (0;1)	1.5 (0.7;1.6)	3.3 (1.5;4.5)	0 (0;2)	0.5 (0;3)	4 (2;6.3)	3.5 (2;5)	4 (2;5.3)	0 (0;0)	7 (7;9)
	**0**	8.6 (4.6;14.6)	2.4 (1.7;3.2)	0 (0;1)	0 (0;1.5)	1 (0.4;1.9)	2.4 (0.9;4.5)	2 (0;4.5)	5 (3;6.5)	3 (1;5.8)	3 (1.8;4.8)	3 (1.8;4.8)	0 (0;0)	7.5 (6.8;9.3)
**bDMARDs**	**1**	17 (10.6;29.6)	3.5 (2.4;3.9)	0 (0;1.8)	0 (0;1)	1.5 (1.2;1.7)	3.5 (2.7;4.5)	1 (0;2)	1 (0;3)	4 (3;7)	4 (3;5.5)	4 (3.5;6.5)	0 (0;2)	7 (7;8.5)
	**0**	5.1 (3;12.1)	2.2 (1.9;2.9)	0 (0;1)	0 (0;1)	0.5 (0.2;1.6)	1.2 (0.7;3.8)	0 (0;4)	2 (0;6)	2 (1;5)	2 (1;4)	2 (1;4)	0 (0;0)	8 (7;9)
**Axial involvement**	**1**	11.5 (7.3;18.3)	2.5 (2.2;3.1)	0 (0;0.1)	0 (0;0.3)	1.5 (0.9;1.7)	3.4 (1.9;4.5)	1 (0;4)	3.5 (1.8;6.5)	4 (2;6.3)	4 (2.8;5)	4 (2.8;5.5)	0 (0;0)	7.5 (6.8;9.3)
	**0**	13.7 (2.3;29.4)	3.5 (1.9;4.3)	2.4 (0.3;3.3)	1 (0;2.3)	0.6 (0.2;1.7)	1.3 (0.6;3.9)	0 (0;0.5)	0 (0;0)	1.5 (0;5.5)	2 (0.8;5.3)	2 (0.8;5.3)	0 (0;0)	7 (7;8.3)
**Comorbidities**	**1**	11 (5.2;25.5)	2.5 (2.1;3.8)	0 (0;1.8)	0 (0;1)	0.9 (0.6;2.3)	2 (1.4;5.5)	1 (0;4)	3 (0;7)	2 (2;7.5)	3 (2;6)	3 (2;7)	0 (0;1)	8 (7;9.5)
	**0**	12 (8;19.3)	2.7 (2.2;3.5)	0 (0;0.5)	0 (0;1)	1.5 (0.2;1.5)	3.3 (0.7;3.7)	0 (0;1)	0 (0;2)	4 (0;5)	4 (1;4)	4 (1;4)	0 (0;0)	7 (6;7)
**Metabolic syndrome**	**1**	7.7 (5.1;11.8)	2.3 (1.9;2.6)	0 (0;0.4)	0 (0;0.8)	0.8 (0.4;1.5)	1.8 (1;3.5)	0 (0;3.3)	2.5 (0.3;5.5)	2 (1.3;4)	2.5 (2;3.8)	2.5 (2;4)	0 (0;0)	7.5 (6.3;9.8)
	**0**	20.7 (10.3;30.2)	3.8 (2.7;4.8)	1.1 (0;2.7)	0.5 (0;1)	1.5 (0.9;2.6)	4.1 (2.1;6)	0.5 (0;2)	0 (0;2.8)	5.5 (2.5;7.8)	5 (2.5;5.8)	5 (2.5;6.8)	0 (0;0)	7 (7;8.8)
**Cardiovascular comorbidities**	**1**	17 (6.6;29.6)	3.5 (2.2;4.3)	1.8 (0;2.4)	1 (0;1.5)	1.5 (0.6;2.4)	4.5 (1.4;5.8)	2 (0.5;4)	3 (0;8)	6 (2;7.5)	5 (2;6)	5 (2;7.5)	0 (0;3)	9 (8;9.5)
	**0**	11 (5.1;19.3)	2.4 (2.2;3.5)	0 (0;0.5)	0 (0;1)	1.4 (0.2;1.5)	3.3 (0.7;3.7)	0 (0;1)	1 (0;3)	4 (1;5)	3 (1;4)	4 (1;4)	0 (0;0)	7 (6;7)
**W0 elevated IL-1β**	**1**	11.6 (10.8;16.3)	2.8 (2.6;3.4)	0.3 (0;0.8)	0.5 (0;1)	1.6 (1.4;1.9)	3.6 (3;4.6)	0.5 (0;1.8)	0.5 (0;2.3)	4.5 (3.5;5.8)	3.5 (3;5)	4 (3.8;5)	0 (0;0)	7 (6.8;8.5)
	**0**	8 (5.1;24.7)	2.4 (1.9;3.5)	0 (0;2.4)	0 (0;1)	0.7 (0.3;1.5)	1.7 (0.8;4.1)	0 (0;2)	2 (0;3.5)	2 (1;5.5)	2 (1.5;5)	2 (1.5;5)	0 (0;0)	8 (7;9)
**W0 elevated IL-6**	**1**	24.2 (16.5;29.4)	4.3 (3.1;5.2)	0.9 (0;3.1)	0.5 (0;13.3)	1.8 (1.5;2.3)	4.1 (3.6;5.1)	0 (0;0)	0 (0;0)	6 (4.8;7.3)	5.5 (4.8;6.5)	5.5 (4.8;6.5)	0 (0;1.3)	7 (6.5;7)
	**0**	11 (5.1;19.5)	2.5 (2.2;3.5)	0 (0;1.4)	0 (0;1)	0.9 (0.3;1.6)	2 (0.8;4.2)	1 (0;3)	2 (0;5)	2 (1;5.5)	3 (1.5;4.5)	3 (1.5;4.5)	0 (0;0)	8 (7;9.5)
**W0 elevated fecal calprotectin**	**1**	5.1 (4.1;8.7)	2.2 (1.9;2.7)	0.4 (0;2.5)	0 (0;3.5)	0.5 (0.3;1.2)	1.2 (0.8;2.8)	0 (0;2)	2 (0;5)	2 (1;3.5)	2 (1.5;3)	2 (1.5;3)	0 (0;0)	9 (7.5;10)
	**0**	17 (10.2;29)	3.5 (2.4;4.1)	0 (0;1.8)	0 (0;1)	1.5 (0.9;1.9)	3.5 (2;4.5)	1 (0;2)	1 (0;3)	4 (2;7)	4 (3;6)	4 (3;7)	0 (0;1)	7 (7;8)
**W0 MDA**	**1**	5.2 (3.5;8)	2.1 (1.7;2.4)	0.2 (0;2.5)	0 (0;1)	0.5 (0.2;0.7)	1.1 (0.7;1.7)	0 (0;1.5)	0 (0;2)	1.5 (0.3;2)	2 (1;2)	2 (1;2)	0 (0;0)	7.5 (6.3;9)
	**0**	20.7 (13.4;29.8)	3.5 (2.8;4.8)	0 (0;1.5)	0 (0;1)	1.8 (1.5;2.6)	4.5 (3.7;6)	1 (0;3.5)	3 (0.3;7.5)	6.5 (5;7.8)	5 (4.3;6.8)	5.5 (4.3;7)	0 (0;4.3)	7 (7;8.8)
**W0 PASS**	**1**	5.1 (3;8)	1.9 (1.7;2.4)	0.4 (0;3)	0 (0;1)	0.4 (0.2;0.7)	1 (0.7;1.7)	0 (0;2)	0 (0;2)	1 (0;2)	2 (1;2)	2 (1;2)	0 (0;0)	8 (6;9)
	**0**	19.3 (12.1;29.6)	3.5 (2.6;4.6)	0 (0;1.2)	0 (0;1)	1.6 (1.5;2.4)	4.5 (3.6;5.5)	1 (0;3)	3 (0;7)	6 (4.5;7.5)	5 (4;6.5)	5 (4;7)	0 (0;4)	7 (7;8.5)
**W0 physical activity°**	**1**	8 (5.2;17.5)	2.4 (2;3.5)	0 (0;0.9)	0 (0;0.8)	1.1 (0.6;1.5)	2.5 (1.3;3.6)	0 (0;4)	1 (0;5.3)	3 (2;6.3)	3 (2;5.5)	3 (2;5.5)	0 (0;1.3)	8.5 (7;9.8)
	**0**	14.6 (10.4;30.2)	2.8 (2.3;4.8)	0.3 (0;1.8)	0.5 (0;1)	1.5 (0.4;2)	3.6 (1;4.5)	0.5 (0;1.8)	1.5 (0;3.8)	4.5 (1.3;5.8)	3.5 (1.5;5)	4 (1.5;5)	0 (0;0)	7 (6.3;7.8)

Gender "1" refers to male, "0" refers to female; for the other variables "1" refers to "yes", "0" refers to "no".
Data are reported as median and interquartile range.
Significant associations are indicated by green cells. Significance refers to the Kruskal-Wallis test.
° Weekly, according to the Food Frequency Questionnaire.
The subsequent baseline variables were excluded from the analysis of the study group due to inadequate case number: elevated IL-1α, fibromyalgia, uveitis, inflammatory bowel disease, HLA-B27.
W0, week 0; DAPSA, disease activity index in psoriatic arthritis; DAS28-CRP, disease activity score on 28 joints with C reactive protein; PASI, Psoriasis Area Severity Index; BSA, Body Surface Area; ASDAS-CRP, Ankylosing Spondylitis Disease Activity Score – C Reactive Protein; BASDAI, Bath Ankylosing Spondylitis Disease Activity Index; LEI, Leeds Enthesitis Index; SPARCC, Spondylarthritis Research Consortium of Canada; VAS, Visual Analogue Scale; PGA, Physician Global Assessment; PtGA, patient global assessment; WPAI, Work Productivity and Activity Impairment questionnaire; PREDIMED, PREvención con DIeta MEDiterránea; bDMARDs, biological disease-modifying antirheumatic drugs; IL, interleukin; MDA, Minimal Disease Activity; PASS, Patient Acceptable Symptom State.

Table S3c. Association between continuous and categorical variables at W0 (part 3).

		W0 hsCRP	W0 ESR	W0 TNFα	W0 Insulinemia	W0 Total cholesterol	W0 HDL cholesterol	W0 LDL cholesterol	W0 Triglyceride	W0 Uricemia	W0 Blood glucose	W0 Mannitol	W0 Lactulose
**Gender**	**1**	0.2 (0.1;0.3)	9 (3;19)	9.2 (6.4;92)	15.6 (15.3;21.5)	192 (171;220)	49 (44;52)	126 (113;136)	118 (88;131)	0.4 (0.4;0.4)	106 (88;117)	21.1 (16.6;22.8)	0 (0;0.1)
	**0**	0.2 (0.1;0.8)	26 (10;40.5)	14.2 (7.6;94.1)	11.4 (8.3;20)	196 (164;234)	61 (49;67)	112 (90.3;153.5)	91 (81;122.5)	0.3 (0.3;0.4)	99 (89;112.5)	15.3 (10.4;17.8)	0 (0;0.1)
**Smoke ever**	**1**	0.1 (0;0.3)	8.5 (4.3;34.5)	7.8 (6.7;8.9)	15.5 (11.4;19.7)	197 (151.3;215.8)	57 (50.5;62)	113.5 (93.3;123.3)	87 (62;116.5)	0.3 (0.3;0.4)	104.5 (89;122.3)	19.2 (16.3;22.4)	0.1 (0;0.1)
	**0**	0.2 (0.1;0.6)	17 (9.5;27.5)	22 (8.6;98.8)	14.8 (11.3;22.3)	194 (167.3;238.3)	50 (42.5;63.3)	121.5 (101;163.5)	115 (85.8;129.5)	0.4 (0.3;0.4)	100 (87;111.5)	16.7 (10.6;21.1)	0 (0;0.1)
**Higher education**	**1**	0.3 (0.1;0.6)	15 (9;28)	11.4 (7.6;83.1)	15.3 (10;21.5)	191 (162;228)	50 (44;61)	113 (92;130)	88 (80;124)	0.4 (0.3;0.4)	92 (86;110)	17.2 (11.9;21.5)	0 (0;0.1)
	**0**	0.1 (0.1;0.2)	11 (5;42)	28.9 (7;100.3)	15.4 (13.4;20)	220 (194;235.5)	62 (54;65)	136 (111;153.5)	113 (92.5;129.5)	0.3 (0.3;0.4)	115 (103.5;117)	16.6 (12.7;23.3)	0.1 (0;0.1)
**Employed**	**1**	0.3 (0.1;0.7)	17 (8.8;39.8)	10.3 (7.3;42.5)	14.9 (11;22.8)	191.5 (160.5;229)	50.5 (43.5;61.3)	112.5 (91.2;135.3)	102.5 (77.8;128.5)	0.4 (0.3;0.4)	100 (87.5;112.8)	17 (14.4;21.2)	0 (0;0.1)
	**0**	0.1 (0.1;0.1)	7 (2.8;14.8)	93.8 (70.6;108.1)	15.4 (13.5;15.9)	229.5 (214;245)	64 (58.8;66.5)	146 (130;163.3)	103.5 (93.3;134)	0.3 (0.3;0.4)	103.5 (89;130.5)	17.8 (9.6;25.7)	0 (0;0.1)
**bDMARDs**	**1**	0.3 (0.2;1)	13 (8.5;29)	7.6 (6.1;10.3)	14.2 (10.7;22.3)	191 (164;197.5)	50 (41.5;61.5)	112 (89.5;120.5)	88 (71.5;121)	0.4 (0.3;0.4)	97 (87;113.5)	16.8 (13.6;21.3)	0 (0;0.1)
	**0**	0.1 (0.1;0.2)	23 (3;28)	95.5 (83.1;105)	15.4 (12.5;21.5)	232 (196;263)	60 (49;64)	151 (112;183)	113 (91;130)	0.3 (0.3;0.4)	106 (90;117)	18.4 (10.2;25.4)	0 (0;0.1)
**Axial involvement**	**1**	0.1 (0.1;0.4)	10 (7.8;29.3)	12.8 (7.8;95.3)	14.8 (8.4;18.3)	199.5 (184.8;236.8)	56 (49.8;63)	120.5 (105.5;158.5)	120.5 (89.5;136)	0.4 (0.3;0.4)	98 (87.5;111.3)	13.6 (10.2;17.2)	0 (0;0.1)
	**0**	0.3 (0.2;0.4)	21 (10.5;31.8)	17.7 (5.7;86.2)	18.5 (12.2;30.3)	181.5 (155;222)	47.5 (43.3;62.5)	112.5 (95.7;131.5)	85 (67.3;100)	0.4 (0.3;0.4)	109 (90.5;130.5)	21.3 (19.4;25.9)	0 (0;0.1)
**Comorbidities**	**1**	0.1 (0.1;0.7)	11 (7.5;40.5)	14.2 (7;93.8)	15.4 (12.8;20)	203 (192;234)	50 (45;60.5)	130 (111;158.5)	118 (103.5;129)	0.4 (0.3;0.4)	101 (93.5;116)	18.4 (11;24.1)	0 (0;0.1)
	**0**	0.2 (0.1;0.3)	15 (9;26)	11.4 (7.6;83.1)	12.5 (8.5;21.5)	171 (156;228)	62 (47;68)	112 (88.6;115)	85 (80;91)	0.3 (0.3;0.4)	92 (85;112)	16.8 (16;20.1)	0 (0;0.1)
**Metabolic syndrome**	**1**	0.1 (0.1;0.2)	9 (4;23.3)	60.5 (12.1;102.6)	14.8 (9.1;19.6)	226 (191.3;257)	60.5 (51.3;63.5)	143.5 (111.3;176.3)	129 (91.8;148.3)	0.4 (0.3;0.4)	103.5 (91.8;113.8)	15.6 (10.6;16.7)	0 (0;0.1)
	**0**	0.3 (0.1;0.6)	21 (11.5;36.3)	7 (5.9;13.6)	16.5 (11.3;27.8)	181.5 (153;201.3)	47.5 (42.5;59)	112 (91;122.8)	85 (73.3;116)	0.3 (0.3;0.4)	95.5 (86.5;115.8)	21.3 (17.9;25.7)	0 (0;0.1)
**Cardiovascular comorbidities**	**1**	0.2 (0.1;0.7)	19 (7.5;48.5)	9.2 (7;55.3)	15.4 (12.8;19.1)	203 (192;226)	48 (41.5;55)	130 (118;143.5)	118 (105.5;126)	0.4 (0.3;0.4)	101 (93.5;116)	21.1 (12.7;24.1)	0 (0;0.1)
	**0**	0.2 (0.1;0.3)	13 (9;26)	14.2 (7.6;92)	15.3 (8.5;21.5)	191 (162;239)	61 (49;66)	112 (92;156)	88 (80;130)	0.3 (0.3;0.4)	97 (86;112)	16.8 (11.9;20.1)	0 (0;0.1)
**W0 elevated IL-1β**	**1**	0.6 (0.2;1)	17.5 (9;34)	11.1 (7.5;47.2)	22.3 (17.8;25.1)	176.5 (156;203)	56.5 (51;62.8)	103.5 (90.8;125.3)	77 (55;106.8)	0.3 (0.3;0.3)	107 (94.3;118.8)	14 (11.5;17.3)	0.1 (0.1;0.1)
	**0**	0.2 (0.1;0.4)	15 (5;33.5)	15.1 (7.6;93.8)	14.2 (10.7;18.6)	203 (168.5;234)	50 (43;62)	126 (104;158.5)	117 (83.5;129)	0.4 (0.3;0.4)	99 (87;111)	17.2 (12.9;22.1)	0 (0;0.1)
**W0 elevated IL-6**	**1**	0.5 (0.3;0.7)	35.5 (21.5;46.8)	7 (6.3;26.5)	20 (9.6;30.3)	145 (138;171)	55 (47.8;73.8)	87.8 (81.3;94.5)	68 (49.8;80.5)	0.3 (0.2;0.3)	104.5 (89.8;131.8)	19.2 (15.5;22.7)	0.1 (0;0.1)
	**0**	0.1 (0.1;0.4)	11 (7.5;21)	14.2 (7.8;93.8)	15.3 (11.4;19.2)	196 (181;237.5)	51 (43;63)	126 (111;161)	117 (89.5;130.5)	0.4 (0.3;0.4)	99 (87;113.5)	16.8 (13.6;22.1)	0 (0;0.1)
**W0 elevated fecal calprotectin**	**1**	0.1 (0.1;0.2)	26 (13;35)	101 (93.8;107.5)	15.4 (11.7;22.1)	232 (188;251)	51 (48;61)	151 (117;170.5)	94 (86.5;129)	0.3 (0.3;0.4)	101 (88;140.5)	18.4 (10.2;22.8)	0 (0;0.1)
	**0**	0.3 (0.1;1)	11 (8;19)	7.6 (6.2;11.4)	14.2 (11.3;21)	192 (166;203)	52 (42;64)	112 (92;126)	113 (80;124)	0.4 (0.3;0.4)	99 (88;115)	16.8 (15.3;21.5)	0 (0;0.1)
**W0 MDA**	**1**	0.2 (0.1;0.3)	11 (2.3;22)	60.5 (9.3;99.6)	15.4 (11.6;22.3)	206 (164.3;255.3)	55.5 (45.3;61.8)	133 (101.8;175)	106 (82;129.5)	0.4 (0.3;0.4)	99 (87;109)	17.5 (10.9;21.1)	0 (0;0.1)
	**0**	0.3 (0.1;0.9)	20.5 (9.5;36.3)	8.6 (6.7;14.2)	14.9 (10.4;20.1)	194 (172.3;221.8)	51 (47.3;67.5)	112 (94;123.3)	102 (82.8;122.3)	0.3 (0.3;0.4)	107 (89;117)	17 (15.5;22.4)	0.1 (0;0.1)
**W0 PASS**	**1**	0.1 (0.1;0.2)	13 (2;23)	92 (7.6;101)	15.3 (11.3;21.5)	220 (171;263)	51 (44;62)	136 (113;183)	118 (88;130)	0.4 (0.3;0.4)	101 (86;110)	18.4 (10.5;21.5)	0 (0;0.1)
	**0**	0.3 (0.1;1)	15 (9;33.5)	9.2 (7;14.7)	15.6 (10.7;22.3)	192 (164;215.5)	52 (47.5;67)	112 (90.3;120.5)	91 (72.5;120.5)	0.3 (0.3;0.4)	99 (90;117)	16.8 (13.6;21.9)	0 (0;0.1)
**W0 physical activity°**	**1**	0.2 (0.1;0.3)	17 (8;37.3)	9.5 (6.6;99.6)	12.8 (8.9;16.9)	194 (172.5;226)	61 (45.8;65)	112 (101;134.5)	103.5 (81.3;125.5)	0.3 (0.3;0.4)	96.5 (85.3;113.8)	17.8 (15.6;24.1)	0.1 (0;0.1)
	**0**	0.3 (0.1;0.9)	11 (8.3;27.5)	14.7 (8.2;69.6)	18.3 (13.2;28.3)	197 (164.3;238.3)	49.5 (47.3;58.8)	120.5 (97.3;163.5)	104 (83.5;129.3)	0.4 (0.3;0.4)	102.5 (91.8;115.8)	16.3 (10.6;21.4)	0 (0;0.1)

Gender “1” refers to male, “0” refers to female; for the other variables “1” refers to “yes”, “0” refers to “no”. Data are reported as median and interquartile range.
Significant associations are indicated by green cells. Significance refers to the Kruskal-Wallis test.
° Weekly, according to the Food Frequency Questionnaire.

The subsequent baseline variables were excluded from the analysis of the study group due to inadequate case number: elevated IL-1α, fibromyalgia, uveitis, inflammatory bowel disease, HLA-B27.
W0, week 0; hsCRP, High Sensitivity C Reactive Protein; ESR, Erythrocyte Sedimentation Rate; TNFα, Tumor Necrosis Factor alpha; HDL, High Density Lipoprotein; LDL, Low Density Lipoprotein; bDMARDs, biological disease-modifying antirheumatic drugs; IL, interleukin; MDA, Minimal Disease Activity; PASS, Patient Acceptable Symptom State.

Table S3d. Association between continuous and categorical variables at W0 (part 4).

		W0 Albumin	W0 α1-globulin	W0 α2-globulin	W0 β1-globulin	W0 β2-globulin	W0 γ-globulin	W0 WBC	W0 RBC	W0 Hb	W0 platelet	W0 Neutrophils	W0 Lymphocytes
**Gender**	**1**	61 (59.4;63)	3.5 (3.4;4)	8.6 (8.3;9.4)	5.8 (5.5;6.4)	5.8 (5.1;6.5)	14 (13;14.9)	6.2 (5.2;6.6)	5.1 (5;5.3)	158 (154;163)	261 (230;275)	3.1 (2.5;4)	1.5 (1.3;2.7)
	**0**	58.7 (55.3;59.9)	3.6 (3.2;4.1)	9.6 (8;9.8)	6.1 (5.9;6.3)	5.3 (4.9;6.4)	16.7 (14.8;19.7)	6.3 (4.9;6.6)	4.4 (4.3;4.6)	134 (132;144)	252 (188.5;294)	2.7 (2.4;3.2)	2.2 (1.8;2.8)
**Smoke ever**	**1**	60.3 (58.3;61.4)	3.6 (3.5;4.5)	8.7 (7.9;9.5)	5.9 (5.7;6.2)	5.2 (5;6.2)	14.7 (14.1;16.1)	6.4 (5.2;7)	4.8 (4.6;5)	148.5 (144.3;152.8)	218 (182;263)	3.1 (2.8;3.9)	1.4 (1.3;2.6)
	**0**	59.6 (57.9;62.5)	3.6 (3.2;4)	9.1 (8.2;9.8)	6.2 (5.5;6.3)	5.9 (4.9;6.4)	16 (12.9;18.9)	6.2 (5;6.4)	4.7 (4.4;5.1)	145.5 (133.3;161)	257.5 (220.5;306.5)	2.8 (2.3;3.3)	2.2 (1.8;2.7)
**Higher education**	**1**	59.5 (58.7;63)	3.8 (3.5;4.2)	9.4 (8.6;9.8)	5.9 (5.7;6.2)	5.1 (4.8;6.2)	14.5 (13;16.7)	6.2 (4.9;6.7)	4.6 (4.4;5)	145 (133;158)	254 (174;308)	3 (2.4;3.4)	1.8 (1.4;2.7)
	**0**	59.7 (54.7;60.8)	3.2 (3.2;3.2)	8.1 (7.6;9.2)	6.3 (5.8;6.5)	5.8 (5.3;6.9)	16.1 (14.5;19.1)	6.2 (5.7;6.4)	5 (4.7;5.2)	154 (140;161)	252 (235;272.5)	2.7 (2.6;3.5)	2.2 (1.8;2.8)
**Employed**	**1**	59.5 (57.8;61.6)	3.6 (3.5;4.2)	8.8 (8;9.7)	6.2 (5.8;6.3)	5.9 (5;6.5)	15.2 (13.3;17.4)	6.3 (4.9;6.8)	4.7 (4.4;5.1)	146.5 (133;158.5)	253 (198;283.3)	2.9 (2.4;3.6)	2 (1.4;3)
	**0**	60.6 (57.9;61.9)	3.2 (3.1;3.4)	9.2 (8.1;9.9)	5.7 (5.4;6.1)	5.3 (5;5.6)	15.5 (14.4;18.7)	6.1 (5.8;6.3)	5 (4.7;5.1)	150 (143;158.8)	244 (211.3;289)	2.9 (2.5;3.3)	2.4 (2;2.6)
**bDMARDs**	**1**	60 (58.9;62.2)	3.6 (3.5;4.6)	9.4 (8.2;10.3)	5.9 (5.6;6.3)	5.1 (4.8;6.2)	14 (13;16.8)	6.3 (4.9;6.9)	4.6 (4.4;5.1)	148 (138;159)	230 (172.5;289.5)	3 (2.8;3.7)	1.5 (1.4;2.4)
	**0**	59.4 (53;61.5)	3.2 (3.2;3.8)	8.6 (7.8;9.8)	6.2 (6;6.4)	6 (5.3;6.5)	16.1 (14.9;21)	6.2 (5.2;6.4)	4.9 (4.4;5.1)	146 (134;158)	254 (240;275)	2.7 (2.3;3.1)	2.5 (2.2;2.7)
**Axial involvement**	**1**	59.9 (58.4;63.1)	3.6 (3.2;3.9)	9.2 (7.8;9.9)	5.9 (5.6;6.1)	5.3 (4.9;6.3)	15.3 (13.3;17.4)	6.3 (4.8;6.6)	4.6 (4.4;4.9)	144 (133.8;148.3)	233 (195;310.3)	2.7 (2.3;3.1)	2.4 (1.8;3)
	**0**	59.3 (57.5;61.1)	3.7 (3.4;4.4)	8.7 (8.5;9.5)	6.3 (6;6.4)	6 (5.2;6.5)	15.4 (13.7;17.3)	6.1 (5.1;6.6)	5 (4.6;5.2)	158 (148.3;160.8)	257.5 (237.5;274.3)	3.5 (2.8;4.3)	1.4 (1.4;2.3)
**Comorbidities**	**1**	59.7 (58.2;62.3)	3.5 (3.2;4.2)	9.8 (8.2;10.6)	6 (5.7;6.3)	5.3 (5;6.1)	14.9 (13.6;16.9)	6.4 (6.2;6.8)	5 (4.5;5.2)	149 (141.5;161)	271 (241;312.5)	3.2 (2.8;4.1)	2.2 (1.6;2.8)
	**0**	59.5 (57.9;61)	3.6 (3.5;3.8)	8.8 (7.6;9.4)	6.2 (5.7;6.3)	6.1 (5;6.5)	15.8 (12.9;18.4)	4.9 (4.9;6.2)	4.6 (4.4;4.7)	143 (133;158)	206 (174;254)	2.5 (2.3;2.9)	2.1 (1.4;2.5)
**Metabolic syndrome**	**1**	61.3 (58.2;63.3)	3.4 (3.2;3.8)	8.7 (7.9;9.8)	6.2 (5.5;6.4)	5.6 (4.7;6.4)	14.7 (12.9;17)	5.7 (4.8;6.4)	4.7 (4.4;5.1)	143 (133.3;160)	227 (179;266.3)	2.6 (2.2;3)	2.2 (1.6;2.6)
	**0**	59.3 (58.1;59.7)	3.7 (3.5;4.3)	9.1 (8.4;9.8)	6 (5.8;6.3)	5.7 (5;6.4)	16 (13.8;17.9)	6.4 (6.2;7.7)	4.8 (4.5;5.1)	147.5 (143.5;158)	268 (236;314.8)	3.1 (2.8;4.6)	2.2 (1.4;2.9)
**Cardiovascular comorbidities**	**1**	59.1 (57.5;60.8)	3.4 (3.2;3.9)	8.3 (8;10.1)	5.9 (5.7;6.4)	5.3 (5.2;6.8)	14.9 (13.9;18.3)	6.4 (6.3;7.5)	5 (4.7;5.2)	154 (147;161)	271 (256.5;291)	4 (3;5)	1.8 (1.4;3)
	**0**	59.7 (57.9;63)	3.6 (3.5;4)	9.4 (8.6;9.8)	6.1 (5.7;6.3)	5.8 (4.8;6.5)	15.8 (12.9;16.7)	5.2 (4.9;6.3)	4.6 (4.4;4.9)	143 (133;158)	214 (174;275)	2.7 (2.3;3)	2.2 (1.5;2.6)
**W0 elevated IL-1β**	**1**	58.7 (55.3;61.6)	4.2 (3.4;5)	9.8 (8.3;11)	5.6 (5.4;5.8)	6.5 (5.7;6.7)	14.3 (13.4;17.5)	5.6 (4.8;6.7)	4.5 (4.3;4.8)	141 (133.8;151)	170 (136.3;209.8)	2.9 (2.3;4)	1.5 (1.2;1.9)
	**0**	59.5 (58.3;62.3)	3.6 (3.3;3.9)	8.6 (8;9.7)	6.2 (5.9;6.4)	5.3 (4.9;6.2)	15.8 (13.2;17.8)	6.2 (5.1;6.7)	4.7 (4.4;5.1)	149 (135.5;160)	261 (227;291.5)	2.9 (2.6;3.6)	2.2 (1.5;3)
**W0 elevated IL-6**	**1**	57.1 (55.4;58.3)	4.3 (3.8;5)	9.3 (8.5;10.2)	6.1 (5.8;6.3)	5.8 (5;6.7)	17.5 (15.9;19.3)	7.3 (6.3;8)	4.5 (4.4;4.8)	137 (129.3;147.3)	218 (198;236)	3.9 (2.9;5.1)	2 (1.3;2.7)
	**0**	60.9 (59.3;63.2)	3.5 (3.2;3.9)	8.8 (8.2;9.8)	6 (5.6;6.3)	5.3 (5;6.2)	14.5 (13;16.4)	6.2 (4.9;6.4)	4.9 (4.5;5.1)	149 (141.5;160)	271 (208.5;312.5)	2.8 (2.3;3.3)	2.1 (1.5;2.6)
**W0 elevated fecal calprotectin**	**1**	57.6 (52.8;60.5)	3.5 (3.2;3.8)	8.6 (8.2;9.3)	6.2 (5.8;6.4)	6.5 (5.7;6.5)	16.7 (15.4;21.5)	6.2 (5.5;6.5)	4.7 (4.4;5)	138 (132.5;156)	254 (233;274.5)	2.7 (2.2;3.6)	2.5 (2.2;2.8)
	**0**	60 (59.1;63.4)	3.6 (3.4;4.3)	9.4 (8.1;9.8)	5.9 (5.7;6.3)	5.2 (4.8;6.1)	14 (12.9;16.5)	6.2 (4.9;6.7)	4.7 (4.5;5.1)	148 (143;160)	240 (174;308)	2.9 (2.7;3.4)	1.8 (1.4;2.6)
**W0 MDA**	**1**	60.2 (59.2;62.6)	3.6 (3.3;4)	8.6 (7.9;9.7)	6.2 (6;6.5)	5.6 (4.9;6.1)	15.4 (12.9;18)	6.2 (5.4;6.4)	4.8 (4.6;5.2)	156 (139.3;161.8)	256.5 (220.5;274.8)	2.9 (2.5;3.3)	2.2 (1.5;2.9)
	**0**	59.2 (56.7;60.8)	3.6 (3.3;4.2)	9.2 (8.3;9.8)	5.9 (5.6;6.2)	5.8 (5.1;6.5)	15.3 (13.8;17)	6.3 (4.9;7.7)	4.5 (4.4;5)	145.5 (133.3;148.8)	242 (181.3;298.8)	2.9 (2.5;4.4)	2 (1.4;2.6)
**W0 PASS**	**1**	59.5 (59.1;61.5)	3.5 (3.2;3.8)	8.6 (7.8;9.4)	6.2 (5.9;6.5)	5.8 (5;6.1)	15.8 (13;18.4)	6.2 (5.2;6.4)	5 (4.7;5.2)	158 (143;163)	261 (240;275)	2.7 (2.5;3.1)	2.2 (1.5;3)
	**0**	59.7 (57.1;62.2)	3.6 (3.4;4.6)	9.6 (8.5;10.3)	5.9 (5.6;6.3)	5.3 (5;6.5)	14.5 (13.6;16.8)	6.3 (4.9;7.5)	4.5 (4.3;5)	145 (133;148.5)	230 (172.5;289.5)	2.9 (2.5;4.1)	1.8 (1.5;2.6)
**W0 physical activity°**	**1**	59.5 (58.2;59.9)	3.5 (3.2;3.6)	8.6 (8.2;9.7)	6.2 (5.9;6.3)	5.3 (4.9;5.8)	16.6 (15.9;18.1)	6.2 (5.2;6.3)	4.8 (4.5;5.1)	144.5 (134.3;157)	266 (217.5;274.8)	2.7 (2.4;2.9)	2.4 (1.5;3)
	**0**	61 (56.9;63.3)	3.9 (3.5;4.3)	9.1 (8;10.7)	5.9 (5.6;6.3)	6.2 (5.3;6.5)	13.6 (12.9;14.4)	6.5 (5;7.7)	4.7 (4.4;5.1)	148.5 (136.8;159.5)	235 (205.8;294.5)	3.2 (2.6;4.5)	2 (1.4;2.4)

Gender “1” refers to male, “0” refers to female; for the other variables “1” refers to “yes”, “0” refers to “no”. Data are reported as median and interquartile range.
Significant associations are indicated by green cells. Significance refers to the Kruskal-Wallis test.
° Weekly, according to the Food Frequency Questionnaire.
The subsequent baseline variables were excluded from the analysis of the study group due to inadequate case number: elevated IL-1α, fibromyalgia, uveitis, inflammatory bowel disease, HLA-B27.
W0, week 0; WBC, white blood cells; RBC, red blood cells; Hb; hemoglobin; bDMARDs, biological disease-modifying antirheumatic drugs; IL, interleukin; MDA, Minimal Disease Activity; PASS, Patient Acceptable Symptom State.
