# Supplementary material for: Ketogenic diet improves disease activity and cardiovascular risk in psoriatic arthritis: A proof of concept study
Source: PLoS One. 2025 Apr 22;20(4):e0321140. doi: 10.1371/journal.pone.0321140 (PMC12013891; doi:10.1371/journal.pone.0321140)
Supplement: S4 Table — (PDF) [file pone.0321140.s004.pdf]

Table S4a. Association between continuous variables at W0 (part 1).

		W0 Weight	W0 BMI	W0 Abdominal circumference	W0 Age	DD Arthritis	DD Psoriasis	W0 DAPSA	W0 DAS28-CRP	W0 PASI	W0 BSA	W0 BASDAI	W0 ASDAS-CRP	W0 LEI	W0 SPARCC	W0 VAS pain	W0 PGA	W0 PtGA
**W0 Weight**	**Spearman's r_s**	1.000	0.714	0.818	-0.260	-0.359	-0.093	0.289	0.203	0.379	0.350	0.093	0.095	-0.336	-0.475	-0.042	0.071	0.065
	**p***		0.000	0.000	0.268	0.120	0.696	0.217	0.391	0.099	0.131	0.697	0.691	0.147	0.034	0.861	0.765	0.785
**W0 BMI**	**Spearman's r_s**		1.000	0.805	0.069	-0.526	-0.116	0.586	0.474	0.354	0.414	0.367	0.390	0.069	0.083	0.253	0.345	0.360
	**p***			0.000	0.771	0.017	0.627	0.007	0.035	0.125	0.070	0.111	0.089	0.774	0.728	0.282	0.136	0.119
**W0 Abdominal circumference**	**Spearman's r_s**			1.000	0.035	-0.409	-0.102	0.325	0.261	0.332	0.339	0.144	0.140	-0.117	-0.138	0.006	0.131	0.103
	**p***				0.884	0.073	0.667	0.161	0.266	0.153	0.144	0.546	0.557	0.622	0.563	0.978	0.581	0.664
**W0 Age**	**Spearman's r_s**				1.000	0.017	0.181	-0.076	0.011	-0.140	-0.019	0.150	0.188	0.151	0.284	0.226	0.207	0.178
	**p***					0.942	0.444	0.750	0.962	0.557	0.937	0.528	0.426	0.525	0.225	0.339	0.381	0.454
**DD Arthritis**	**Spearman's r_s**					1.000	-0.062	-0.373	-0.173	0.132	0.092	-0.087	-0.075	-0.036	0.008	-0.050	-0.198	-0.118
	**p***						0.794	0.105	0.466	0.580	0.701	0.716	0.753	0.882	0.974	0.834	0.403	0.621
**DD Psoriasis**	**Spearman's r_s**						1.000	-0.108	-0.029	0.063	0.151	0.061	0.023	-0.233	-0.106	0.095	0.041	0.082
	**p***							0.649	0.905	0.792	0.524	0.799	0.923	0.322	0.657	0.689	0.865	0.731
**W0 DAPSA**	**Spearman's r_s**							1.000	0.892	0.168	0.251	0.772	0.741	0.391	0.361	0.712	0.734	0.762
	**p***								0.000	0.479	0.286	0.000	0.000	0.089	0.118	0.000	0.000	0.000
**W0 DAS28-CRP**	**Spearman's r_s**								1.000	0.392	0.428	0.710	0.711	0.402	0.271	0.663	0.634	0.678
	**p***									0.087	0.060	0.000	0.000	0.079	0.248	0.001	0.003	0.001
**W0 PASI**	**Spearman's r_s**									1.000	0.947	-0.027	0.010	-0.099	-0.338	-0.130	-0.125	-0.051
	**p***										0.000	0.909	0.967	0.679	0.145	0.584	0.598	0.832
**W0 BSA**	**Spearman's r_s**										1.000	0.129	0.168	-0.078	-0.216	0.029	0.013	0.112
	**p***											0.588	0.480	0.745	0.359	0.905	0.958	0.637
**W0 BASDAI**	**Spearman's r_s**											1.000	0.983	0.305	0.350	0.976	0.957	0.978
	**p***												0.000	0.191	0.131	0.000	0.000	0.000
**W0 ASDAS-CRP**	**Spearman's r_s**												1.000	0.295	0.319	0.959	0.932	0.962
	**p***													0.207	0.171	0.000	0.000	0.000
**W0 LEI**	**Spearman's r_s**													1.000	0.686	0.325	0.243	0.269
	**p***														0.001	0.161	0.302	0.251
**W0 SPARCC**	**Spearman's r_s**														1.000	0.368	0.256	0.310
	**p***															0.110	0.275	0.183
**W0 VAS pain**	**Spearman's r_s**															1.000	0.966	0.982

	p*	0.000	0.000
**W0 PGA**	**Spearman's r_s**	1.000	0.978
	**p***		0.000
**W0 PtGA**	**Spearman's r_s**		1.000
	**p***		
**W0 hsCRP**	**Spearman's r_s**		
	**p***		
**W0 ESR**	**Spearman's r_s**		
	**p***		
**W0 TNFα**	**Spearman's r_s**		
	**p***		
**W0 Insulinemia**	**Spearman's r_s**		
	**p***		
**W0 WPAI lost work hours**	**Spearman's r_s**		
	**p***		
**W0 WPAI impact**	**Spearman's r_s**		
	**p***		
**W0 PREDIMED**	**Spearman's r_s**		
	**p***		
**W0 SCORE2¤^**	**Spearman's r_s**		
	**p***		
**W0 CUORE§**	**Spearman's r_s**		
	**p***		
**W0 SBP**	**Spearman's r_s**		
	**p***		
**W0 DBP**	**Spearman's r_s**		
	**p***		

* Significance refers to the Spearman correlation test, indicated by the coefficient r_s.¤ Computed from 19 subjects.

§ 10 year risk of cardiovascular events according to the Progetto estimator. SCORE2-OP (Older People) estimator was used for subjects >70 years. Values were adjusted for subjects with inflammatory arthritis. Probability is expressed as percentage of risk.

^ 10 year risk of cardiovascular events according to the ESC (European Society of Cardiology), SCORE2 (Systematic Coronary Risk Evaluation 2) estimator. Values were adjusted for subjects with inflammatory arthritis. Probability is expressed as percentage of risk.

The subsequent baseline variables were excluded from the analysis of the study group due to inadequate case number: elevated IL-1α, fibromyalgia, uveitis, inflammatory bowel disease, HLA-B27.

W0, week 0; BMI, body mass index; DD, disease duration; DAPSA, disease activity index in psoriatic arthritis; DAS28-CRP, disease activity score on 28 joints with C reactive protein; PASI, Psoriasis Area Severity Index; BSA, Body Surface Area; BASDAI, Bath Ankylosing Spondylitis Disease Activity Index; ASDAS-CRP, Ankylosing Spondylitis Disease Activity Score – C Reactive Protein; LEI, Leeds Enthesitis Index;

SPARCC, Spondylarthritis Research Consortium of Canada; VAS, Visual Analogue Scale; PGA, Physician Global Assessment; PtGA, patient global assessment; hsCRP, High Sensitivity C Reactive Protein; ESR, Erythrocyte Sedimentation Rate; TNFα, Tumor Necrosis Factor alpha; WPAI, Work Productivity and Activity Impairment questionnaire; PREDIMED, PREvención con DIeta MEDiterránea; SCORE2, systematic coronary risk evaluation; CUORE, cardiovascular unique offer reengineered; SBP, systolic blood pressure; DBP, diastolic blood pressure.

Table S4b. Association between continuous variables at W0 (part 2).

		W0 hsCRP	W0 ESR	W0 TNFα	W0 Insulinemia	W0 WPAI lost work hours	W0 WPAI impact	W0 PREDIMED	W0 SCORE2¤^	W0 CUORE§	W0 PAS	W0 PAD
**W0 Weight**	**Spearman's r_s**	0.225	-0.097	-0.178	0.490	-0.371	0.229	-0.309	0.046	0.011	-0.033	0.008
	**p***	0.340	0.684	0.452	0.028	0.157	0.361	0.185	0.853	0.963	0.889	0.972
**W0 BMI**	**Spearman's r_s**	0.350	0.174	-0.098	0.472	0.026	0.519	-0.042	-0.035	-0.001	0.040	-0.047
	**p***	0.130	0.463	0.682	0.035	0.924	0.027	0.859	0.887	0.997	0.866	0.843
**W0 Abdominal circumference**	**Spearman's r_s**	0.036	-0.168	-0.058	0.617	-0.242	0.299	-0.195	-0.041	0.078	-0.029	-0.013
	**p***	0.879	0.480	0.808	0.004	0.367	0.229	0.410	0.867	0.750	0.904	0.957
**W0 Age**	**Spearman's r_s**	-0.089	0.066	0.158	-0.084	0.253	0.288	0.045	0.559	0.441	0.261	0.165
	**p***	0.709	0.781	0.505	0.726	0.345	0.246	0.850	0.013	0.059	0.267	0.486
**DD Arthritis**	**Spearman's r_s**	-0.214	-0.075	0.235	-0.217	-0.060	-0.073	0.075	0.011	0.468	0.353	0.468
	**p***	0.366	0.755	0.318	0.359	0.824	0.774	0.754	0.963	0.043	0.127	0.037
**DD Psoriasis**	**Spearman's r_s**	0.142	0.362	0.327	0.054	0.175	-0.185	0.237	0.021	-0.029	0.350	0.303
	**p***	0.551	0.117	0.160	0.821	0.517	0.462	0.315	0.932	0.906	0.131	0.193
**W0 DAPSA**	**Spearman's r_s**	0.421	0.322	-0.476	0.235	0.488	0.733	0.055	-0.252	-0.191	-0.239	-0.344
	**p***	0.065	0.167	0.034	0.319	0.055	0.001	0.816	0.298	0.432	0.310	0.138
**W0 DAS28-CRP**	**Spearman's r_s**	0.512	0.493	-0.450	0.228	0.449	0.711	0.199	-0.176	-0.133	-0.080	-0.169
	**p***	0.021	0.027	0.047	0.334	0.081	0.001	0.401	0.472	0.588	0.736	0.476
**W0 PASI**	**Spearman's r_s**	0.322	0.279	-0.058	0.330	-0.185	0.306	0.157	-0.085	0.069	0.328	0.347
	**p***	0.167	0.234	0.809	0.155	0.494	0.216	0.508	0.731	0.779	0.159	0.134
**W0 BSA**	**Spearman's r_s**	0.323	0.263	-0.027	0.240	-0.068	0.428	0.085	-0.034	0.140	0.307	0.360
	**p***	0.165	0.262	0.911	0.307	0.803	0.076	0.723	0.891	0.567	0.188	0.119
**W0 BASDAI**	**Spearman's r_s**	0.251	0.313	-0.297	-0.045	0.475	0.625	0.128	0.059	0.045	-0.076	-0.167
	**p***	0.286	0.180	0.204	0.851	0.063	0.006	0.591	0.810	0.855	0.751	0.481
**W0 ASDAS-CRP**	**Spearman's r_s**	0.314	0.347	-0.250	-0.049	0.461	0.651	0.107	0.074	0.004	-0.044	-0.131
	**p***	0.177	0.134	0.287	0.836	0.073	0.003	0.654	0.763	0.987	0.854	0.582
**W0 LEI**	**Spearman's r_s**	-0.105	0.097	-0.239	0.042	0.322	0.172	0.475	-0.061	0.149	-0.032	-0.032
	**p***	0.659	0.684	0.310	0.860	0.224	0.496	0.034	0.806	0.544	0.893	0.894
**W0 SPARCC**	**Spearman's r_s**	-0.126	0.055	0.143	-0.175	0.370	0.174	0.317	-0.187	0.035	-0.242	-0.273
	**p***	0.596	0.817	0.549	0.460	0.159	0.491	0.173	0.442	0.888	0.303	0.244

**W0 VAS pain**	**Spearman's r_s**	0.198	0.332	-0.326	-0.116	0.587	0.631	0.110	0.079	0.094	-0.011	-0.127
	**p***	0.403	0.153	0.161	0.627	0.017	0.005	0.646	0.747	0.701	0.962	0.595
**W0 PGA**	**Spearman's r_s**	0.184	0.259	-0.396	-0.035	0.552	0.620	0.060	0.102	0.058	-0.033	-0.189
	**p***	0.438	0.270	0.084	0.885	0.026	0.006	0.801	0.677	0.815	0.892	0.425
**W0 PtGA**	**Spearman's r_s**	0.245	0.286	-0.368	-0.042	0.585	0.666	0.054	0.062	0.089	0.021	-0.102
	**p***	0.298	0.221	0.111	0.861	0.017	0.003	0.822	0.801	0.719	0.930	0.670
**W0 hsCRP**	**Spearman's r_s**	1.000	0.618	-0.193	0.179	0.059	0.222	0.007	0.122	-0.406	0.071	0.021
	**p***		0.004	0.416	0.449	0.829	0.375	0.975	0.618	0.085	0.767	0.930
**W0 ESR**	**Spearman's r_s**		1.000	0.106	0.075	0.082	0.149	0.439	-0.051	-0.332	0.128	-0.042
	**p***			0.658	0.755	0.762	0.555	0.053	0.834	0.165	0.589	0.859
**W0 TNFα**	**Spearman's r_s**			1.000	-0.126	-0.231	-0.365	0.270	-0.143	-0.119	0.015	0.021
	**p***				0.595	0.389	0.137	0.250	0.559	0.629	0.949	0.931
**W0 Insulinemia**	**Spearman's r_s**				1.000	-0.250	0.161	-0.205	-0.247	-0.073	0.344	0.256
	**p***					0.350	0.523	0.386	0.307	0.767	0.138	0.276
**W0 WPAI lost work hours**	**Spearman's r_s**					1.000	0.489	0.196	-0.248	0.125	0.157	0.011
	**p***						0.054	0.468	0.373	0.656	0.562	0.968
**W0 WPAI impact**	**Spearman's r_s**						1.000	-0.335	-0.283	0.116	0.077	0.015
	**p***							0.175	0.271	0.659	0.763	0.953
**W0 PREDIMED**	**Spearman's r_s**							1.000	0.017	-0.011	0.051	-0.056
	**p***								0.944	0.965	0.833	0.815
**W0 SCORE2¤^**	**Spearman's r_s**								1.000	0.453	0.160	0.164
	**p***									0.051	0.513	0.502
**W0 CUORE§**	**Spearman's r_s**									1.000	0.569	0.633
	**p***										0.011	0.004
**W0 SBP**	**Spearman's r_s**										1.000	0.905
	**p***											0.000
**W0 DBP**	**Spearman's r_s**											1.000
	**p***											

* Significance refers to the Spearman correlation test, indicated by the coefficient r_s.
¤ Computed from 19 subjects.
§ 10 year risk of cardiovascular events according to the Progetto estimator. SCORE2-OP (Older People) estimator was used for subjects >70 years. Values were adjusted for subjects with inflammatory arthritis. Probability is expressed as percentage of risk.
^ 10 year risk of cardiovascular events according to the ESC (European Society of Cardiology), SCORE2 (Systematic Coronary Risk Evaluation 2) estimator. Values were adjusted for subjects with inflammatory arthritis. Probability is expressed as percentage of risk.

The subsequent baseline variables were excluded from the analysis of the study group due to inadequate case number: elevated IL-1α, fibromyalgia, uveitis, inflammatory bowel disease, HLA-B27.
W0, week 0; BMI, body mass index; DD, Significant associations are indicated by green cells. Significance refers to the Kruskal-Wallis test.; DAPSA, disease activity index in psoriatic arthritis; DAS28-CRP, disease activity score on 28 joints with C reactive protein; PASI, Psoriasis Area Severity Index; BSA, Body Surface Area; BASDAI, Bath Ankylosing Spondylitis Disease Activity Index; ASDAS-CRP, Ankylosing Spondylitis Disease Activity Score – C Reactive Protein; LEI, Leeds Enthesitis Index; SPARCC, Spondylarthritis Research Consortium of Canada; VAS, Visual Analogue Scale; PGA, Physician Global Assessment; PtGA, patient global assessment; hsCRP, High Sensitivity C Reactive Protein; ESR, Erythrocyte Sedimentation Rate; TNFα, Tumor Necrosis Factor alpha; WPAI, Work Productivity and Activity Impairment questionnaire; PREDIMED, PREvención con DIeta MEDiterránea; SCORE2, systematic coronary risk evaluation; CUORE, cardiovascular unique offer reengineered; SBP, systolic blood pressure; DBP, diastolic blood pressure.

Table S4c. Association between continuous variables at W0 (part 3).

		W0 Total cholesterol	W0 HDL cholesterol	W0 LDL cholesterol	W0 Triglyceride	W0 Uricemia	W0 Albumin	W0 α1-globulin	W0 α2-globulin	W0 β1-globulin	W0 β2-globulin	W0 γ-globulin	W0 WBC	W0 Hb	W0 Platelet	W0 Neutrophils	W0 Lymphocytes
**W0 Age**	**Spearman's r_s**	0.374	0.363	0.287	0.231	-0.270	-0.119	-0.170	0.245	-0.202	-0.012	0.142	-0.225	-0.025	-0.241	-0.071	-0.267
	**p***	0.105	0.115	0.220	0.328	0.250	0.617	0.475	0.297	0.393	0.958	0.549	0.340	0.917	0.306	0.767	0.255
**DD Arthritis**	**Spearman's r_s**	0.209	-0.196	0.347	0.162	0.382	-0.164	-0.150	-0.012	-0.388	0.144	0.119	0.362	0.503	0.220	0.167	0.239
	**p***	0.376	0.409	0.133	0.494	0.096	0.490	0.528	0.960	0.091	0.544	0.618	0.116	0.024	0.352	0.482	0.310
**DD Psoriasis**	**Spearman's r_s**	0.336	0.081	0.327	-0.032	-0.275	-0.190	0.321	0.316	0.114	-0.050	0.059	0.156	-0.398	-0.005	0.290	-0.002
	**p***	0.148	0.733	0.160	0.892	0.241	0.423	0.168	0.174	0.632	0.835	0.806	0.512	0.082	0.982	0.214	0.995
**W0 Weight**	**Spearman's r_s**	-0.505	-0.072	-0.396	-0.427	-0.076	0.208	0.270	0.002	0.081	0.162	-0.347	0.038	0.024	-0.227	0.317	-0.428
	**p***	0.023	0.762	0.084	0.060	0.750	0.380	0.249	0.992	0.733	0.494	0.133	0.875	0.920	0.336	0.173	0.060
**W0 BMI**	**Spearman's r_s**	-0.334	-0.171	-0.304	-0.146	-0.075	0.012	0.146	0.117	0.188	0.398	-0.197	0.003	-0.144	-0.084	0.232	-0.439
	**p***	0.150	0.472	0.193	0.539	0.752	0.960	0.539	0.622	0.428	0.082	0.405	0.990	0.543	0.724	0.326	0.053
**W0 Abdominal circumference**	**Spearman's r_s**	-0.295	-0.173	-0.206	-0.093	0.046	0.257	0.086	0.101	0.223	0.230	-0.347	0.039	0.157	0.045	0.266	-0.301
	**p***	0.207	0.465	0.384	0.698	0.846	0.274	0.717	0.673	0.344	0.328	0.134	0.870	0.508	0.850	0.257	0.198
**W0 DAPSA**	**Spearman's r_s**	-0.296	-0.276	-0.351	-0.248	-0.150	-0.268	0.387	0.327	-0.011	0.133	0.005	0.332	-0.221	0.143	0.395	-0.253
	**p***	0.204	0.239	0.129	0.292	0.528	0.254	0.092	0.160	0.965	0.576	0.985	0.152	0.349	0.548	0.084	0.281
**W0 DAS28-CRP**	**Spearman's r_s**	-0.219	-0.419	-0.199	-0.189	-0.061	-0.444	0.378	0.428	-0.139	0.255	0.125	0.263	-0.062	0.268	0.386	-0.351
	**p***	0.354	0.066	0.401	0.424	0.798	0.050	0.101	0.060	0.560	0.277	0.600	0.262	0.796	0.254	0.092	0.129
**W0 PASI**	**Spearman's r_s**	-0.228	-0.533	0.034	-0.145	0.116	-0.303	0.238	0.247	0.122	0.389	0.091	0.100	0.179	0.391	0.487	-0.459
	**p***	0.335	0.015	0.888	0.542	0.626	0.194	0.312	0.293	0.609	0.090	0.704	0.673	0.449	0.088	0.029	0.042
**W0 BSA**	**Spearman's r_s**	-0.106	-0.431	0.118	-0.034	0.078	-0.293	0.260	0.307	0.087	0.370	0.075	0.134	0.069	0.324	0.522	-0.505
	**p***	0.655	0.058	0.619	0.886	0.744	0.210	0.268	0.188	0.715	0.108	0.752	0.574	0.772	0.164	0.018	0.023
**W0 BASDAI**	**Spearman's r_s**	-0.210	0.007	-0.331	-0.189	-0.287	-0.316	0.200	0.267	-0.338	0.100	0.185	0.421	-0.281	0.014	0.306	-0.074
	**p***	0.375	0.976	0.154	0.424	0.220	0.175	0.397	0.255	0.144	0.674	0.435	0.065	0.230	0.955	0.189	0.758
**W0 ASDAS-CRP**	**Spearman's r_s**	-0.230	0.034	-0.346	-0.196	-0.292	-0.365	0.158	0.265	-0.340	0.177	0.224	0.355	-0.306	-0.048	0.243	-0.117
	**p***	0.330	0.886	0.135	0.407	0.211	0.114	0.505	0.258	0.142	0.454	0.341	0.125	0.189	0.842	0.301	0.624
**W0 LEI**	**Spearman's r_s**	0.188	-0.261	0.210	0.427	0.218	-0.206	-0.479	-0.255	-0.064	0.207	0.339	0.049	0.090	0.328	-0.038	0.064
	**p***	0.427	0.266	0.374	0.061	0.355	0.385	0.032	0.279	0.788	0.382	0.144	0.838	0.704	0.158	0.874	0.787
**W0 SPARCC**	**Spearman's r_s**	0.433	-0.103	0.346	0.541	0.238	-0.032	-0.255	0.038	-0.137	0.094	0.025	0.043	-0.051	0.297	-0.204	0.271
	**p***	0.056	0.664	0.135	0.014	0.312	0.892	0.278	0.875	0.566	0.692	0.916	0.858	0.830	0.204	0.389	0.248
**W0 VAS pain**	**Spearman's r_s**	-0.128	0.082	-0.288	-0.156	-0.286	-0.344	0.174	0.256	-0.323	0.076	0.235	0.384	-0.285	-0.016	0.244	-0.046
	**p***	0.590	0.730	0.218	0.512	0.222	0.138	0.464	0.277	0.165	0.749	0.320	0.095	0.223	0.947	0.301	0.846

**W0 PGA**	**Spearman's r_s**	-0.272	0.094	-0.452	-0.256	-0.374	-0.274	0.205	0.242	-0.221	0.004	0.209	0.380	-0.292	-0.062	0.280	-0.064
	**p***	0.245	0.692	0.045	0.275	0.104	0.242	0.385	0.304	0.349	0.986	0.376	0.099	0.211	0.795	0.232	0.788
**W0 PtGA**	**Spearman's r_s**	-0.226	0.032	-0.372	-0.204	-0.281	-0.283	0.243	0.297	-0.246	0.064	0.162	0.414	-0.283	-0.033	0.329	-0.105
	**p***	0.337	0.894	0.106	0.388	0.230	0.226	0.302	0.203	0.296	0.787	0.496	0.069	0.227	0.891	0.156	0.659
**W0 hsCRP**	**Spearman's r_s**	-0.349	-0.196	-0.313	-0.439	-0.311	-0.264	0.612	0.576	-0.157	0.112	-0.191	0.032	-0.303	-0.298	0.245	-0.558
	**p***	0.131	0.408	0.179	0.053	0.183	0.260	0.004	0.008	0.510	0.638	0.420	0.894	0.194	0.202	0.299	0.011
**W0 ESR**	**Spearman's r_s**	0.002	-0.059	-0.011	-0.365	-0.365	-0.765	0.306	0.315	0.000	0.520	0.340	-0.011	-0.505	0.109	0.039	-0.265
	**p***	0.992	0.806	0.962	0.113	0.113	0.000	0.190	0.176	0.999	0.019	0.143	0.962	0.023	0.647	0.870	0.260
**W0 TNFα**	**Spearman's r_s**	0.433	0.167	0.452	0.196	0.026	-0.187	-0.288	-0.184	0.093	0.340	0.174	-0.101	-0.254	0.022	-0.297	0.400
	**p***	0.057	0.482	0.045	0.407	0.914	0.431	0.217	0.436	0.696	0.142	0.464	0.672	0.281	0.927	0.203	0.080
**W0 Insulinemia**	**Spearman's r_s**	-0.454	-0.415	-0.265	-0.195	-0.053	-0.023	0.214	0.212	0.426	0.386	-0.157	0.360	0.094	0.112	0.431	-0.096
	**p***	0.044	0.069	0.259	0.410	0.825	0.922	0.364	0.370	0.061	0.093	0.508	0.119	0.694	0.638	0.058	0.686
**W0 WPAI lost work hours**	**Spearman's r_s**	0.326	-0.168	0.091	0.082	0.195	-0.138	0.111	0.181	0.197	-0.156	0.082	0.203	-0.026	0.108	0.224	0.022
	**p***	0.218	0.533	0.739	0.763	0.470	0.610	0.683	0.501	0.464	0.564	0.763	0.451	0.924	0.691	0.404	0.937
**W0 WPAI impact**	**Spearman's r_s**	-0.059	-0.047	-0.156	-0.129	-0.100	-0.291	0.216	0.334	0.081	0.235	0.078	0.296	-0.045	0.077	0.460	-0.322
	**p***	0.817	0.852	0.536	0.610	0.693	0.241	0.390	0.176	0.748	0.348	0.758	0.232	0.858	0.761	0.055	0.193
**W0 PREDIMED**	**Spearman's r_s**	0.243	-0.287	0.272	0.124	0.072	-0.440	-0.306	-0.210	-0.110	0.220	0.484	-0.057	-0.066	0.417	-0.049	0.140
	**p***	0.302	0.220	0.246	0.603	0.763	0.052	0.190	0.373	0.644	0.352	0.031	0.811	0.781	0.067	0.839	0.555
**W0 SCORE2¤^**	**Spearman's r_s**	-0.057	0.342	-0.034	-0.030	-0.274	0.103	0.020	0.099	-0.623	-0.221	-0.043	-0.347	0.083	-0.550	-0.112	-0.519
	**p***	0.818	0.152	0.889	0.903	0.256	0.676	0.936	0.686	0.004	0.363	0.861	0.146	0.734	0.015	0.647	0.023
**W0 CUORE§**	**Spearman's r_s**	0.259	-0.096	0.367	0.426	0.361	0.123	-0.223	-0.167	-0.234	0.062	0.021	0.181	0.575	-0.067	0.290	-0.192
	**p***	0.284	0.697	0.122	0.069	0.128	0.616	0.359	0.493	0.334	0.802	0.932	0.459	0.010	0.786	0.229	0.430
**W0 SBP**	**Spearman's r_s**	-0.075	-0.278	0.066	0.099	0.090	-0.145	-0.010	0.087	0.111	0.292	0.149	0.324	0.245	-0.071	0.431	-0.126
	**p***	0.754	0.235	0.782	0.679	0.705	0.542	0.967	0.717	0.640	0.212	0.529	0.163	0.298	0.768	0.058	0.597
**W0 DBP**	**Spearman's r_s**	0.087	-0.237	0.265	0.246	0.248	0.038	-0.112	0.023	0.053	0.206	-0.009	0.294	0.378	-0.043	0.452	-0.192
	**p***	0.715	0.314	0.260	0.295	0.291	0.873	0.637	0.924	0.823	0.385	0.969	0.208	0.100	0.858	0.045	0.418
**W0 Total cholesterol**	**Spearman's r_s**	1.000	0.219	0.924	0.618	0.303	-0.008	-0.329	-0.106	0.058	-0.038	0.038	-0.257	0.050	0.233	-0.214	0.079
	**p***		0.353	0.000	0.004	0.194	0.975	0.157	0.656	0.807	0.874	0.875	0.275	0.835	0.324	0.366	0.742
**W0 HDL cholesterol**	**Spearman's r_s**		1.000	-0.036	-0.229	-0.522	0.139	-0.207	-0.134	-0.067	-0.259	-0.024	-0.457	-0.509	-0.475	-0.453	-0.059
	**p***			0.880	0.331	0.018	0.558	0.380	0.574	0.777	0.271	0.920	0.043	0.022	0.034	0.045	0.806
**W0 LDL cholesterol**	**Spearman's r_s**			1.000	0.670	0.451	-0.041	-0.301	-0.096	0.027	0.108	0.029	-0.176	0.225	0.326	-0.077	0.010
	**p***				0.001	0.046	0.863	0.198	0.687	0.910	0.649	0.902	0.458	0.340	0.161	0.748	0.966

**W0 Triglyceride**	**Spearman's r_s**	1.000	0.584	0.253	-0.500	-0.264	-0.021	0.009	0.039	-0.075	0.391	0.248	-0.208	0.252
	**p***		0.007	0.283	0.025	0.260	0.930	0.970	0.870	0.753	0.088	0.292	0.380	0.284
**W0 Uricemia**	**Spearman's r_s**		1.000	0.291	-0.235	-0.185	0.075	0.182	-0.260	-0.030	0.714	0.428	-0.081	0.122
	**p***			0.213	0.318	0.436	0.753	0.444	0.269	0.899	0.000	0.060	0.733	0.610
**W0 Albumin**	**Spearman's r_s**			1.000	-0.067	-0.015	0.005	-0.607	-0.771	-0.194	0.365	-0.146	-0.104	0.006
	**p***				0.778	0.950	0.982	0.005	0.000	0.413	0.114	0.539	0.663	0.980
**W0 α1-globulin**	**Spearman's r_s**				1.000	0.773	-0.056	-0.179	-0.400	0.258	-0.158	-0.140	0.440	-0.358
	**p***					0.000	0.814	0.451	0.081	0.272	0.507	0.556	0.052	0.122
**W0 α2-globulin**	**Spearman's r_s**					1.000	-0.113	-0.149	-0.452	0.127	-0.052	0.046	0.341	-0.407
	**p***						0.636	0.531	0.045	0.593	0.829	0.848	0.141	0.075
**W0 β1-globulin**	**Spearman's r_s**						1.000	0.225	-0.005	-0.016	-0.170	0.299	0.084	0.100
	**p***							0.340	0.982	0.947	0.474	0.200	0.724	0.676
**W0 β2-globulin**	**Spearman's r_s**							1.000	0.369	0.069	-0.099	0.234	-0.050	-0.030
	**p***								0.110	0.771	0.677	0.320	0.835	0.899
**W0 γ-globulin**	**Spearman's r_s**								1.000	0.122	-0.266	0.105	-0.080	0.305
	**p***									0.609	0.258	0.659	0.738	0.190
**W0 WBC**	**Spearman's r_s**									1.000	0.130	0.150	0.749	0.344
	**p***										0.584	0.527	0.000	0.138
**W0 Hb**	**Spearman's r_s**										1.000	0.269	0.191	-0.033
	**p***											0.252	0.420	0.890
**W0 Platelet**	**Spearman's r_s**											1.000	0.126	0.254
	**p***												0.596	0.279
**W0 Neutrophils**	**Spearman's r_s**												1.000	-0.253
	**p***													0.281
**W0 Lymphocytes**	**Spearman's r_s**													1.000
	**p***													

* Significance refers to the Spearman correlation test, indicated by the coefficient r_s.¤ Computed from 19 subjects.

§ 10 year risk of cardiovascular events according to the Progetto estimator. SCORE2-OP (Older People) estimator was used for subjects >70 years. Values were adjusted for subjects with inflammatory arthritis. Probability is expressed as percentage of risk. ^ 10 year risk of cardiovascular events according to the ESC (European Society of Cardiology), SCORE2 (Systematic Coronary Risk Evaluation 2) estimator. Values were adjusted for subjects with inflammatory arthritis. Probability is expressed as percentage of risk.

The subsequent baseline variables were excluded from the analysis of the study group due to inadequate case number: elevated IL-1α, fibromyalgia, uveitis, inflammatory bowel disease, HLA-B27.

W0, week 0; BMI, body mass index; DD, disease duration; DAPSA, disease activity index in psoriatic arthritis; DAS28-CRP, disease activity score on 28 joints with C reactive protein; PASI, Psoriasis Area Severity Index; BSA, Body Surface Area; BASDAI, Bath Ankylosing Spondylitis Disease Activity Index; ASDAS-CRP, Ankylosing Spondylitis Disease Activity Score – C Reactive Protein; LEI, Leeds Enthesitis Index; SPARCC, Spondylarthritis Research Consortium of Canada; VAS, Visual Analogue Scale; PGA, Physician Global Assessment; PtGA, patient global assessment; hsCRP, High Sensitivity C Reactive Protein; ESR, Erythrocyte Sedimentation Rate; TNFα, Tumor Necrosis Factor alpha; WPAI, Work Productivity and Activity Impairment questionnaire; PREDIMED, PREvención con DIeta MEDiterránea; SCORE2, systematic

coronary risk evaluation; CUORE, cardiovascular unique offer reengineered; SBP, systolic blood pressure; DBP, diastolic blood pressure; HDL, High Density Lipoprotein; LDL, Low Density Lipoprotein; WBC, white blood cells; Hb; hemoglobin.
