# Supplementary material for: Ketogenic diet improves disease activity and cardiovascular risk in psoriatic arthritis: A proof of concept study
Source: PLoS One. 2025 Apr 22;20(4):e0321140. doi: 10.1371/journal.pone.0321140 (PMC12013891; doi:10.1371/journal.pone.0321140)
Supplement: S5 Table — Association between categorical variables at W0. (PDF) [file pone.0321140.s005.pdf]

Table S5. Association between categorical variables at W0.

		Gender 1	Smoke ever 1	Higher education 1	Employed 1	bDMARDs 1	Axial involvement 1	Comorbidities 1	Metabolic syndrome 1	Cardiovascular comorbidities 1	W0 MDA 1	W0 PASS 1	W0 elevated IL-1β	W0 elevated IL-6	W0 elevated fecal calprotectin	W0 physical activity°
**Gender**	**1**	9 (100)	4 (44.4)	6 (66.7)	7 (77.8)	5 (55.6)	3 (3.3)	5 (55.6)	4 (44.4)	4 (44.4)	6 (66.7)	6 (66.7)	2 (22.2)	1 (11.1)	3 (33.3)	3 (33.3)
	**0**	0 (0)	2 (18.2)	7 (63.6)	9 (81.8)	6 (54.5)	9 (81.8)	6 (54.5)	6 (54.5)	3 (27.3)	3 (27.3)	4 (36.4)	2 (18.2)	3 (27.3)	4 (36.4)	7 (63.6)
**Smoke ever**	**1**	4 (66.7)	6 (100)	4 (66.7)	5 (83.3)	5 (83.3)	3 (50)	3 (50)	2 (33.3)	3 (50)	2 (33.3)	2 (33.3)	2 (33.3)	3 (50)	1 (16.7)	3 (50)
	**0**	5 (35.7)	0 (0)	9 (64.3)	11 (78.6)	6 (42.9)	9 (64.3)	8 (57.1)	8 (57.1)	4 (28.6)	7 (50)	8 (57.1)	2 (14.3)	1 (7.1)	6 (42.9)	7 (50)
**Higher education**	**1**	6 (46.2)	4 (30.8)	13 (100)	12 (92.3)	9 (69.2)	8 (61.5)	6 (46.2)	5 (38.5)	3 (23.1)	6 (46.2)	7 (53.8)	2 (15.4)	3 (23.1)	4 (30.8)	6 (46.2)
	**0**	3 (42.9)	2 (28.6)	0 (0)	4 (57.1)	2 (28.6)	4 (57.1)	5 (71.4)	5 (71.4)	4 (57.1)	3 (42.9)	3 (42.9)	2 (28.6)	1 (14.3)	3 (42.9)	4 (57.1)
**Employed**	**1**	7 (43.8)	5 (31.3)	12 (75)	16 (100)	11 (68.8)	9 (56.3)	8 (50)	7 (43.8)	6 (37.5)	7 (43.8)	8 (50)	3 (18.8)	4 (25)	4 (25)	8 (50)
	**0**	2 (50)	1 (25)	1 (25)	0 (0)	0 (0)	3 (75)	3 (75)	3 (75)	1 (25)	2 (50)	2 (50)	1 (25)	0 (0)	3 (75)	2 (50)
**bDMARDs**	**1**	5 (45.5)	5 (45.5)	9 (81.8)	11 (100)	11 (100)	7 (63.6)	6 (54.5)	4 (36.4)	5 (45.5)	3 (27.3)	4 (36.4)	3 (27.3)	3 (27.3)	0 (0)	5 (45.5)
	**0**	4 (44.4)	1 (11.1)	4 (44.4)	5 (55.6)	0 (0)	5 (55.6)	5 (55.6)	6 (66.7)	2 (22.2)	6 (66.7)	6 (66.7)	1 (11.1)	1 (11.1)	7 (77.8)	5 (55.6)
**Axial involvement**	**1**	3 (25)	3 (25)	8 (66.7)	9 (75)	7 (58.3)	12 (100)	8 (66.7)	8 (66.7)	4 (33.3)	4 (33.3)	5 (41.7)	3 (25)	1 (8.3)	4 (33.3)	6 (50)
	**0**	6 (75)	3 (37.5)	5 (62.5)	7 (87.5)	4 (50)	0 (0)	3 (37.5)	2 (25)	3 (37.5)	5 (62.5)	5 (62.5)	1 (12.5)	3 (37.5)	3 (37.5)	4 (50)
**Comorbidities**	**1**	5 (45.5)	3 (27.3)	6 (54.5)	8 (72.7)	6 (54.5)	8 (72.7)	11 (100)	6 (54.5)	7 (63.6)	5 (45.5)	6 (54.5)	2 (18.2)	1 (9.1)	4 (36.4)	6 (54.5)
	**0**	4 (44.4)	3 (33.3)	7 (77.8)	8 (88.9)	5 (55.6)	4 (44.4)	0 (0)	4 (44.4)	0 (0)	4 (44.4)	4 (44.4)	2 (22.2)	3 (33.3)	3 (33.3)	4 (44.4)
**Metabolic syndrome**	**1**	4 (40)	2 (20)	5 (50)	7 (70)	4 (40)	8 (80)	6 (60)	10 (100)	3 (30)	5 (50)	6 (60)	3 (30)	0 (0)	5 (50)	5 (50)
	**0**	5 (50)	4 (40)	8 (80)	9 (90)	7 (70)	4 (40)	5 (50)	0 (0)	4 (40)	4 (40)	4 (40)	1 (10)	4 (40)	2 (20)	5 (50)
**Cardiovascular comorbidities**	**1**	4 (57.1)	3 (42.9)	3 (42.9)	6 (85.7)	5 (71.4)	4 (57.1)	7 (100)	3 (42.9)	7 (100)	3 (42.9)	3 (42.9)	1 (14.3)	1 (14.3)	2 (28.6)	4 (57.1)
	**0**	5 (38.5)	3 (23.1)	10 (76.9)	10 (76.9)	6 (46.2)	8 (61.5)	4 (30.8)	7 (53.8)	0 (0)	6 (46.2)	7 (53.8)	3 (23.1)	3 (23.1)	5 (38.5)	6 (46.2)
**W0 MDA**	**1**	6 (66.7)	2 (22.2)	6 (66.7)	7 (77.8)	3 (33.3)	4 (44.4)	5 (55.6)	5 (55.6)	3 (33.3)	9 (100)	9 (100)	0 (0)	1 (11.1)	5 (55.6)	6 (66.7)
	**0**	3 (27.3)	4 (36.4)	7 (63.6)	9 (81.8)	8 (72.7)	8 (72.7)	6 (54.5)	5 (45.5)	4 (36.4)	0 (0)	1 (9.1)	4 (36.4)	3 (27.3)	2 (18.2)	4 (36.4)
**W0 PASS**	**1**	6 (60)	2 (20)	7 (70)	8 (80)	4 (40)	5 (50)	6 (60)	6 (60)	3 (30)	9 (90)	10 (100)	1 (10)	1 (10)	5 (50)	6 (60)
	**0**	3 (30)	4 (40)	6 (60)	8 (80)	7 (70)	7 (70)	5 (50)	4 (40)	4 (40)	0 (0)	0 (0)	3 (30)	3 (30)	2 (20)	4 (40)
**W0 elevated IL-1β**	**1**	2 (50)	2 (50)	2 (50)	3 (75)	3 (75)	3 (75)	2 (50)	3 (75)	1 (25)	0 (0)	1 (25)	4 (100)	1 (25)	1 (25)	0 (0)
	**0**	7 (46.7)	4 (26.7)	11 (73.3)	13 (86.7)	8 (53.3)	8 (53.3)	8 (53.3)	7 (46.7)	6 (40)	9 (60)	9 (60)	0 (0)	3 (20)	6 (40)	9 (60)
**W0 elevated IL-6**	**1**	1 (25)	3 (75)	3 (75)	4 (100)	3 (75)	1 (25)	1 (25)	0 (0)	1 (25)	1 (25)	1 (25)	1 (25)	4 (100)	1 (25)	2 (50)
	**0**	8 (53.3)	3 (20)	10 (66.7)	11 (73.3)	8 (53.3)	10 (66.7)	9 (60)	9 (60)	5 (33.3)	7 (46.7)	8 (53.3)	3 (20)	0 (0)	5 (33.3)	7 (46.7)
**W0 elevated fecal calprotectin**	**1**	3 (42.9)	1 (14.3)	4 (57.1)	4 (57.1)	0 (0)	4 (57.1)	4 (57.1)	5 (71.4)	2 (28.6)	5 (71.4)	5 (71.4)	1 (14.3)	1 (14.3)	7 (100)	4 (57.1)
	**0**	6 (46.2)	5 (38.5)	9 (69.2)	12 (92.3)	11 (84.6)	8 (61.5)	7 (53.8)	5 (38.5)	5 (38.5)	4 (30.8)	5 (38.5)	3 (23.1)	3 (23.1)	0 (0)	6 (46.2)
**W0 physical activity°**	**1**	3 (30)	3 (30)	6 (60)	8 (80)	5 (50)	6 (60)	6 (60)	5 (50)	4 (40)	6 (60)	6 (60)	0 (0)	2 (20)	4 (40)	10 (100)
	**0**	6 (60)	3 (30)	7 (70)	8 (80)	6 (60)	6 (60)	5 (50)	5 (50)	3 (30)	3 (30)	4 (40)	4 (40)	2 (20)	3 (30)	0 (0)

Gender “1” refers to male, “0” refers to female; for the other variables “1” refers to “yes”, “0” refers to “no”.
Data are reported as number and percentage.
Significance refers to the Chi square test.
° Weekly, according to the Food Frequency Questionnaire.
The subsequent baseline variables were excluded from the analysis of the study group due to inadequate case number: elevated IL-1α, fibromyalgia, uveitis, inflammatory bowel disease, HLA-B27.
bDMARDs, biological disease-modifying antirheumatic drugs; W0, week 0; IL, interleukin; MDA, Minimal Disease Activity; PASS, Patient Acceptable Symptom State; CUORE, cardiovascular unique offer reengineered; SCORE2, systematic coronary risk evaluation.
