# Supplementary material for: Ketogenic diet improves disease activity and cardiovascular risk in psoriatic arthritis: A proof of concept study
Source: PLoS One. 2025 Apr 22;20(4):e0321140. doi: 10.1371/journal.pone.0321140 (PMC12013891; doi:10.1371/journal.pone.0321140)
Supplement: S6 Table — (PDF) [file pone.0321140.s006.pdf]

Table S6. Modification of anthropometric measurements during the study.

	W0	W9	Δ (W9-W0)	p*
Weight, kg, median (IQR)	91 (81.8;99.7)	83.6 (70,5;88,5)	-10.2 (-12.6;-7.7)	<0.001
Height, cm, median (IQR)	173 (164;177.3)	173 (164.8;177.3)	0 (0;0)	NA
BMI, kg/m^2, median (IQR)	30.9 (29.1;33)	27.2 (25.8;29.7)	-3.5 (-4;-2.6)	<0.001
Abdominal circumference, cm, median (IQR)	106 (103.8;115.3)	96 (91;106)	-11.8 (-14;-10)	<0.001

Continuous variables are expressed as median and interquartile range.

* Significance refers to the tests of comparison between variables at W0 and W9, Wilcoxon test for continuous variables for paired data, Pearson or Chi square test for categorical variables. The significant results are those that have reached a p<0.05.

W0, week 0; W9, week 9; IQR, range interquartile; BMI, body mass index.
