# Supplementary material for: Ketogenic diet improves disease activity and cardiovascular risk in psoriatic arthritis: A proof of concept study
Source: PLoS One. 2025 Apr 22;20(4):e0321140. doi: 10.1371/journal.pone.0321140 (PMC12013891; doi:10.1371/journal.pone.0321140)
Supplement: S7 Table — (PDF) [file pone.0321140.s007.pdf]

Table S7. Modification of clinical variables during the study.

	W0	W9	Δ (W9-W0)	p*
Tender joints count (0-68), median (IQR)	3 (1;6)	0 (0;2.5)	-1 (-2;0)	0.007
Swollen joints count (0-68), median (IQR)	0 (0;2)	0 (0;0.3)	0 (-1.3;0)	0.033
Tender joints count (0-28), median (IQR)	2 (0;6)	0 (0;2.5)	0 (-1.3;0)	0.011
Swollen joints count (0-28), median (IQR)	0 (0;0)	0 (0;0)	0 (0;0)	0.197
Dactylitis, n (%)	0 (0)	0(0)	0 (0)	NA
DAPSA, median (IQR)	11.5 (5.2;23.8)	8.6 (2.1;13.5)	-6.1 (-16.8;3.7)	0.006
DAS28-CRP, median (IQR)	2.6 (2.1;3.7)	2.4 (1.8;2.8)	-0.9 (-1.8;0.7)	0.068
CDAI, median (IQR)	8.5 (4.8;19.3)	7.5 (1.8:12.3)	-4.5 (-10.8;4)	0.011
SDAI, median (IQR)	9.1 (4.9;19.3)	7.6 (1.8;12.3)	-4.1 (-10.5;3.9)	0.017
BASDAI, median (IQR)	3.3 (1,1;4,5)	1.8 (0.8;3)	-1 (-1.8;-0.1)	0.005
ASDAS-CRP, median (IQR)	1.5 (0,5;1,7)	0.9 (0.4;1.4)	-0.4 (-0.7;0)	0.016
SPARCC, median (IQR)	1.5 (0;4.5)	0 (0;4.3)	0 (-3.5;2.5)	0.072
LEI, median (IQR)	0 (0;2.5)	0 (0;2)	0 (-2.5;2)	0.305
BSA, median (IQR)	0 (0;1)	0 (0;1)	0 (-1;0)	0.180
PASI, median (IQR)	0 (0;1.8)	0 (0;0.2)	0 (-1.2;0)	0.027
MDA, n (%)	9 (45.0)	9 (45.0)	2 (10.0)	0.019
PASS, n (%)	10 (50.0)	14 (70.0)	5 (25.0)	0.089
HAQ, median (IQR)	0.4 (0.2;0.8)	0.5 (0.2;0.7)	0 (-0.8;0.4)	0.169
PtGA (0-10 cm), median (IQR)	4 (2;5.3)	3 (1;4.3)	-0.5 (-4;1.5)	0.103
PGA (0-10 cm), median (IQR)	3.5 (2;5)	2.5 (0.8;4)	-0.5 (-4;1.3)	0.038
VAS pain (0-10 cm), median (IQR)	4 (1.8;6.3)	3 (1;4.3)	-0.5 (-4.3;2.3)	0.090
WPAI – Weekly working hours, median (IQR)	40 (30;49.3)	40 (30;42.3)	0 (-3;1)	0.180
WPAI – Lost work hours, median (IQR)	0 (0;0)	0 (0;0)	0 (0;0)	0.196
WPAI – Impact, median (IQR)	4.5 (1.7-6.0)	4 (0;5.0)	0 (-3;1)	0.188

Categorical variables are reported as number and percentage, continuous variables are reported as median and interquartile range.
* Significance refers to the tests of comparison between variables at W0 and W9, Wilcoxon test for continuous variables for paired data, Pearson or Chi square test for categorical variables. The significant results are those that have reached a $p<0.05$.
W0, week 0; W9, week 9; IQR, interquartile range; DAPSA, disease activity index in psoriatic arthritis; DAS28-CRP, disease activity score on 28 joints with C reactive protein; CDAI, clinical disease activity index; SDAI, Simple Disease Activity Index; BASDAI, Bath Ankylosing Spondylitis Disease Activity Index; ASDAS-CRP, Ankylosing Spondylitis Disease Activity Score – C Reactive Protein; SPARCC, Spondylarthritis Research Consortium of Canada; LEI, Leeds Enthesitis Index; BSA, Body Surface Area; PASI, Psoriasis Area Severity Index; MDA, Minimal Disease Activity; PASS, Patient Acceptable Symptom State; HAQ, Health Assessment Questionnaire; PtGA, patient global assessment; PGA, Physician Global Assessment; VAS, Visual Analogue Scale; WPAI, Work Productivity and Activity Impairment questionnaire.
