# Supplementary material for: Ketogenic diet improves disease activity and cardiovascular risk in psoriatic arthritis: A proof of concept study
Source: PLoS One. 2025 Apr 22;20(4):e0321140. doi: 10.1371/journal.pone.0321140 (PMC12013891; doi:10.1371/journal.pone.0321140)
Supplement: S8 Table — (PDF) [file pone.0321140.s008.pdf]

Table S8. Modification of inflammatory biomarkers during the study.

	W0	W9	Δ (W9-W0)	p*
hsCRP, mg/L, median (IQR)	0.2 (0.1;0.4)	0.2 (0.1;0.7)	0 (-0.1;0.1)	0.777
ESR, mm/h, median (IQR)	14 (7.8;30.8)	19 (8.8;42.5)	4 (-0.3;12)	0.055
IL-1α°				NA
<3,9 ng/L, n (%)	19 (100)	19 (100)	0 (0)	
≥ 3,9 ng/L, n (%)	0 (0)	0 (0)	0 (0)	
IL-1β°				0.582
<5,0 ng/L, n (%)	15 (78.9)	18 (94.7)	3 (15.8)	
≥ 5,0 ng/L, n (%)	4 (21.1)	1 (5.3)	-3 (-15.8)	
IL-6°				0.108
<7,0 ng/L, n (%)	16 (84.2)	15 (78.9)	-1 (-5.3)	
≥7,0 ng/L, n (%)	3 (15.8)	4 (21.0)	1 (5.3)	
TNFα, ng/L, median (IQR) °	12.8 (7.3;92.9)	12.5 (6.6;63.5)	-1.7 (-14.4;0.4)	0.227
Fecal calprotectin^				0.001
<70μg/g, n (%)	13 (65.0)	13 (72.2)	0 (-0.55)	
≥70 μg/g, n (%)	7 (35.0)	4 (27.8)	-3 (15.8)	

Categorical variables are reported as number and percentage, continuous variables are reported as median and interquartile range.

* Significance refers to the tests of comparison between variables at W0 and W9, Wilcoxon test for continuous variables for paired data, Pearson or Chi square test for categorical variables. The significant results are those that have reached a p<0.05.° Data calculated from 19 patients. ^ Data calculated from 18 patients.

W0, week 0; W9, week 9; IQR, interquartile range; hsCRP, High Sensitivity C Reactive Protein, ESR, Erythrocyte Sedimentation Rate; IL, interleukin;TNFα, Tumor Necrosis Factor alpha.
