# Supplementary material for: Ketogenic diet improves disease activity and cardiovascular risk in psoriatic arthritis: A proof of concept study
Source: PLoS One. 2025 Apr 22;20(4):e0321140. doi: 10.1371/journal.pone.0321140 (PMC12013891; doi:10.1371/journal.pone.0321140)
Supplement: S9 Table — (PDF) [file pone.0321140.s009.pdf]

Table S9. Modification of laboratory variables during the study.

	W0	W9	Δ (W9-W0)	p*
Total cholesterol, mg/dl, median (IQR)	194 (165;233)	185 (135.3;209.5)	-23 (-41.5;-4)	0.007
HDL cholesterol , mg/dl, median (IQR)	51.5 (46.3;62.5)	52.5 (42.3;64.5)	-3.5 (-9.3;2.3)	0.150
LDL cholesterol , mg/dl, median (IQR)	114 (96.5;152.3)	110 (68.3;136)	-10 (-31.3;-2.5)	0.037
Triglyceride, mg/dl, median (IQR)	103.5 (81.5;128.5)	79 (65;89.5)	-25 (-50.3;-3.8)	0.004
AST, U/L, median (IQR)	25 (20;32)	22 (18.8;30.8)	-1.5 (-4.8;1.3)	0.211
ALT, U/L, median (IQR)	27.5 (17.8;38.3)	24.5 (18.8;32)	0.5 (-10.3;8.3)	0.808
GGT, U/L, median (IQR)	25.5 (17.5;37.3)	14 (12;18.3)	-11 (-22;-6)	<0.001
TSH, mU/L, median (IQR)	1.7 (1.2;2.2)	2.3 (1.4;3.1)	0.1 (0;0.8)	0.025
Creatinine, mg/dl, median (IQR)	0.8 (0.7;0.9)	0.8 (0.7;0.9)	0 (0;0.1)	0.247
Uricemia, mmol/L, median (IQR)	0.4 (0.3;0.4)	0.4 (0.3;0.4)	0 (0;0)	0.861
Blood glucose, mg/dl, median (IQR)	100 (87.5;115.5)	95 (89.5;102.8)	-7 (-16.3;2.5)	0.076
Insulinemia, mU/L, median (IQR)	15.4 (11;21.8)	9.2 (6.6;63.5) °	-5.6 (-9.7;-3.9)	0.000
HOMA-IR, median (IQR)	3.7 (2.4;5.8)	2.3 (1.4;3.4)	-2.1(-1.1;-3.0)	<0.001
Azotemia, mmol/L, median (IQR)	5.8 (5.1;6.9)	6.5 (4.9;7)	0.6 (-1;1.5)	0.370
Blood count				
WBC, 10^9 /L, median (IQR)	6.2 (4.9;6.6)	5.4 (4.5;6.1)	-0.7 (-1.1;-0.1)	0.032
RBC, 10^{12} /L, median (IQR)	4.7 (4.4;5.1)	4.7 (4.5;5)	0 (-0.2;0.1)	0.970
Hb, g/L, median (IQR)	147 (133.8;158.5)	147 (139.5;151.8)	0 (-3;4)	0.831
Hc, L/L, median (IQR)	0.4 (0.4;0.5)	0.4 (0.4;0.5)	0 (0;0)	0.828
MCV, fL, median (IQR)	93.3 (90.2;94.3)	92.9 (91.9;95.1)	0.3 (-1.4;1.3)	0.952
MCH, g/L, median (IQR)	30.9 (29.8;31.5)	30.8 (29.8;31.6)	-0.1 (-0.3;0.3)	0.762
MCHC, g/L, median (IQR)	330 (324.5;335.3)	331 (324.8;334.8)	-0.5 (-4;3.3)	0.631
RDW, %, median (IQR)	13.2 (12.7;13.4)	13.4 (12.9;13.7)	0.3 (0.1;0.5)	0.018
Platelet, 10^9 /L, median (IQR)	253 (205.3;283.3)	225 (176.8;265.5)	-25 (-46.8;-13.5)	<0.001
Neutrophils, 10^9 /L, median (IQR)	2.9 (2.4;3.6)	2.7 (2.3;3)	-0.2 (-0.5;0.1)	0.104
Lymphocytes, 10^9 /L, median (IQR)	2.2 (1.4;2.7)	1.8 (1.2;2.1)	-0.3 (-0.4;-0.1)	0.006
Monocytes, 10^9 /L, median (IQR)	0.5 (0.5;0.5)	0.5 (0.4;0.5)	-0.1 (-0.1;0)	0.023
Eosinophilic, 10^9 /L, median (IQR)	0.2 (0.1;0.2)	0.1 (0.1;0.2)	0 (-0.1;0)	0.111
Basophil, 10^9 /L, median (IQR)	0 (0;0.1)	0 (0;0.1)	0 (0;0)	0.592
Protein profile				
Total proteins, g/L, median (IQR)	70.6 (68.8;74)	69.8 (68.2;74.7)	0 (-1.6;2.3)	0.867
Albumin, median (IQR)	59.6 (57.8;61.9)	60.4 (56.5;62.4)	0.2 (-0.9;1.1)	0.681
α1-globulin, median (IQR)	3.6 (3.2;4.1)	3.8 (3.5;4.3)	0.2 (0;0.4)	0.022
α2-globulin, median (IQR)	8.8 (8;9.8)	8.5 (7.7;9.6)	-0.4 (-0.6;0.4)	0.250
β1-globulin, median (IQR)	6.1 (5.7;6.3)	5.7 (5.5;6.3)	-0.1 (-0.4;0)	0.073
β2-globulin, median (IQR)	5.6 (5;6.5)	5.6 (5.2;6.3)	0 (-0.2;0.1)	0.526
γ-globulin, median (IQR)	15.4 (13.3;17.4)	15.4 (13.2;18.3)	0 (-0.1;0.8)	0.244
Urine test				
pH, median (IQR)	5.5 (5;5.8)	5.5 (5;5.6)	0 (0;-0.5)	0.378
Glucose, mmol/L, median (IQR)	0 (0;0)	0 (0;0)	0 (0;0)	0.998
Protein, g/L, median (IQR)	0 (0;0)	0 (0;0)	0 (0;0)	0.180
Hb, g/L, median (IQR)	0 (0;0)	0 (0;0)	0 (0;0)	0.480
Ketones, g/L, median (IQR)	0 (0;0)	0 (0;0.1)	0 (0;0.1)	0.035
Bilirubin, µmol/L, median (IQR)	0 (0;0)	0 (0;0)	0 (0;0)	0.998
Urobilinogen, µmol/L, median (IQR)	3.4 (3.4;3.4)	3.4 (3.4;3.4)	0 (0;0)	0.317
Specific weight, median (IQR)	1017 (1013.5;1020.5)	1014 (1010.8;1017.3)	-4 (-7;3.5)	0.131
Intestinal permeability test				
Diuresis 6h, ml, median (IQR)	285 (223.8;362.5)	300 (250;452.5)	17.5 (-62.5;107.5)	0.421
Lactulose, %, median (IQR)	0.2 (0.1;0.2)	0.2 (0.1;0.3)	0 (-0.1;0.1)	0.296
Mannitol, %, median (IQR)	17 (11.6;21.8)	13.7 (8.8;17.2)	-3.3 (-8.3;1.7)	0.079
Sucrose, %, median (IQR)	0 (0;0.1)	0 (0;0.1)	0 (0;0)	0.936
Lactulose/mannitol ratio, median (IQR)	0 (0;0)	0 (0;0)	0 (0;0)	0.035

Categorical variables are reported as number and percentage, continuous variables are reported as median and interquartile range.

* Significance refers to the tests of comparison between variables at W0 and W9, Wilcoxon test for continuous variables for paired data, Pearson or Chi square test for categorical variables. The significant results are those that have reached a $p<0.05$.

° Data calculated from 19 patients.

W0, week 0; W9, week 9; IQR, interquartile range; HDL, High Density Lipoprotein; LDL, Low Density Lipoprotein; ALT, alanine aminotransferase; AST, aspartate aminotransferase; GGT, gamma glutamyl transpeptidase; TSH, thyroid-stimulating hormone; HOMA-IR, Homeostatic Model Assessment for Insulin Resistance; WBC, white blood cells; RBC, red blood cells; Hb; hemoglobin; MCV, mean

corpuscular volume; MCH, mean corpuscular hemoglobin; MCHC, mean corpuscular hemoglobin concentration; RDW, red cell distribution width.
