# Supplementary material for: Ketogenic diet improves disease activity and cardiovascular risk in psoriatic arthritis: A proof of concept study
Source: PLoS One. 2025 Apr 22;20(4):e0321140. doi: 10.1371/journal.pone.0321140 (PMC12013891; doi:10.1371/journal.pone.0321140)
Supplement: S10 Table — (PDF) [file pone.0321140.s010.pdf]

Table S10. Modification of nutritional questionnaires during the study.

	W0	W9	Δ (W9-W0)	p*
Food Frequency Questionnaire				
Cereals and derivatives	0.6 (0.3;1)	0 (0;0)	-0.6 (-1;-0.3)	<0.001
Processed cereal products	0.9 (0.1;1)	0 (0;0)	-0.9 (-1;-0.1)	<0.001
Fresh meat	0.6 (0.3;0.7)	0.5 (0.4;1)	0 (-0.2;0.5)	0.174
Processed meat	0.2 (0.1;0.4)	0.3 (0.1;0.4)	0 (-0.2;0.2)	0.571
Seafood	0.1 (0.1;0.3)	0.4 (0.3;0.5)	0.1 (0;0.4)	0.001
Milk and yoghurt	1 (0.1;1)	1 (0;1.1)	0.1 (-0.3;1)	0.078
Dairy products	0.4 (0.3;0.4)	0.1 (0;0.3)	-0.3 (-0.4;0)	0.046
Fresh fruit	1 (0.8;1.6)	0 (0;0)	-1 (-1.6;-0.7)	<0.001
Nuts	0.1 (0.1;0.4)	1 (1;2)	0.8 (0.1;1.2)	<0.001
Vegetables	1 (0.6;2)	2 (1;2)	0 (-0.5;1.1)	0.058
Legumes	0.3 (0.1;0.6)	0 (0;0)	-0.3 (-0.6;-0.1)	<0.001
Eggs	0.1 (0.1;0.2)	0.2 (0.1;0.3)	0.1 (0;0.2)	0.033
Sweets	0.4 (0.1;0.6)	0 (0;0)	-0.4 (-0.6;-0.1)	<0.001
Soda	0 (0;0.1)	0 (0;0)	0 (-0.1;0)	<0.007
Alcoholic beverages	0.1 (0;0.5)	0 (0;0)	0 (-0.5;0)	0.003
Physical activity	3 (3;3)	3 (2;3)	0 (-1,5;0)	0.421
PREDIMED score	7 (7;9)	8.5 (6.8;10)	0.5 (-2.3;2)	0.761

Food intake and physical activity refer to weekly frequency. Data are reported as median and interquartile range.
*Significance refers to Wilcoxon test which evaluate the difference between W9 and W0. The significant results are those that have reached a p<0.05.
PREDIMED, PREvención con DIeta MEDiterránea.
