# Supplementary material for: Ketogenic diet improves disease activity and cardiovascular risk in psoriatic arthritis: A proof of concept study
Source: PLoS One. 2025 Apr 22;20(4):e0321140. doi: 10.1371/journal.pone.0321140 (PMC12013891; doi:10.1371/journal.pone.0321140)
Supplement: S11 Table — (PDF) [file pone.0321140.s011.pdf]

Table S11. Modification of cardiovascular parameters during the study.

	**W0**	**W9**	**Δ (W9-W0)**	**p***
Cardiovascular events, n (%)	0 (0)	0 (0)	0 (0;0)	-
Current smoking, n (%)	0 (0)	0 (0)	0 (0;0)	-
Type 2 diabetes, n (%)	2 (10.0)	2 (10.0)	0 (0;0)	-
Antihypertensive medication use, n (%)	7 (35.0)	7 (35.0)	0 (0;0)	-
SBP, mmHg, median (IQR)	140 (130;145)	130 (120;140)	0 (-6.3;0)	0.246
DBP, mmHg, median (IQR)	85 (80;90)	80 (80;90)	0 (0;5)	0.674
CUORE §, median (IQR)	4,35(2,7;9,05)	3.3 (2.4;7)	-0.4 (-1.5;0.2)	0.045
CUORE class				<0.001
I, n (%)	11 (57.9)	13 (68.4)		
II, n (%)	4 (21.0)	2 (10.5)		
III, n (%)	0 (0)	1 (5.3)		
IV, n (%)	3 (15.8)	2 (10.5)		
V, n (%)	0 (0)	0 (0)		
VI, n (%)	1 (5.3)	1 (5.3)		
SCORE2¤^, median (IQR)	6.9 (3.8;14)	7.4 (4.1;12.3)	-0.2 (-0.7;0.1)	0.009
SCORE2 risk class				<0.001
Low, n (%)	16 (84.2)	16 (84.2)		
Moderate, n (%)	0 (0)	0 (0)		
High, n (%)	2 (10.5)	2 (10.5)		
Very high, n (%)	1 (5.3)	1 (5.3)		

Continuous variables are reported as median and interquartile range, categorical variables are reported as number and percentage.
* Significance refers to the tests of comparison between variables at W0 and W9, Wilcoxon test for continuous variables for paired data, Pearson or Chi square test for categorical variables. The significant results are those that have reached a p<0.05.
¤ 19 subjects included.
§ 10 year risk of cardiovascular events according to the Progetto CUORE estimator. SCORE2-OP (Older People) estimator was used for subjects >70 years. Values were adjusted for subjects with inflammatory arthritis. Probability is expressed as percentage of risk.
^ 10 year risk of cardiovascular events according to the ESC (European Society of Cardiology), SCORE2 (Systematic Coronary Risk Evaluation 2) estimator. Values were adjusted for subjects with inflammatory arthritis. Probability is expressed as percentage of risk.
IQR, interquartile range; SBP, systolic blood pressure; DBP, diastolic blood pressure.
