# Supplementary material for: Ketogenic diet improves disease activity and cardiovascular risk in psoriatic arthritis: A proof of concept study
Source: PLoS One. 2025 Apr 22;20(4):e0321140. doi: 10.1371/journal.pone.0321140 (PMC12013891; doi:10.1371/journal.pone.0321140)
Supplement: S12 Table — (PDF) [file pone.0321140.s012.pdf]

Table S12. Correlation between the modification of anthropometric measurements during the study.

	BMI		Abdominal circumference	
	**Spearman's r_s**	**p***	**Spearman's r_s**	**p***
Weight	0.946	0.000	0.613	0.004
BMI	-	-	0.544	0.013

* Significance refers to the Spearman correlation test, indicated by the coefficient r_s.
BMI, Body Mass Index.
