# Supplementary material for: Ketogenic diet improves disease activity and cardiovascular risk in psoriatic arthritis: A proof of concept study
Source: PLoS One. 2025 Apr 22;20(4):e0321140. doi: 10.1371/journal.pone.0321140 (PMC12013891; doi:10.1371/journal.pone.0321140)
Supplement: S13 Table — (PDF) [file pone.0321140.s013.pdf]

Table S13. Correlation between the modification of anthropometric measurements and the modification of clinical variables during the study.

	Weight		BMI		Abdominal circumference	
	**Spearman's r_S**	**p***	**Spearman's r_S**	**p***	**Spearman's r_S**	**p***
Tender joints count (0-68)	0.282	0.228	0.222	0.346	0.479	0.033
Swollen joints count (0-68)	0.428	0.060	0.389	0.090	0.308	0.186
Tender joints count (0-28)	0.297	0.203	0.305	0.191	0.266	0.256
Swollen joints count (0-28)	0.326	0.161	0.289	0.216	0.241	0.306
DAPSA	0.406	0.076	0.516	0.020	0.360	0.119
DAS28-CRP	0.266	0.257	0.347	0.133	0.337	0.146
CDAI	0.410	0.073	0.485	0.030	0.381	0.097
SDAI	0.403	0.078	0.471	0.036	0.392	0.087
BASDAI	0.034	0.887	0.078	0.745	0.080	0.737
ASDAS-CRP	0.002	0.995	0.076	0.750	-0.094	0.692
SPARCC	-0.135	0.570	0.063	0.792	-0.151	0.526
LEI	-0.008	0.972	0.151	0.525	-0.245	0.297
BSA	0.028	0.906	0.055	0.819	0.079	0.740
PASI	0.068	0.777	0.072	0.762	-0.061	0.798
HAQ	0.328	0.158	0.435	0.055	0.116	0.627
PtGA	0.248	0.292	0.314	0.177	0.141	0.553
PGA	0.218	0.356	0.271	0.248	0.161	0.498
VAS pain	0.257	0.274	0.340	0.143	0.155	0.513
WPAI lost work hours	0.181	0.554	0.262	0.022	0.379	0.669

* Significance refers to the Spearman correlation test, indicated by the coefficient r_S.

BMI, Body Mass Index; DAPSA, disease activity index in psoriatic arthritis; DAS28-CRP, disease activity score on 28 joints with C reactive protein; CDAI, clinical disease activity index; SDAI, Simple Disease Activity Index; BASDAI, Bath Ankylosing Spondylitis Disease Activity Index; ASDAS-CRP, Ankylosing Spondylitis Disease Activity Score – C Reactive Protein; SPARCC, Spondylarthritis Research Consortium of Canada; LEI, Leeds Enthesitis Index; BSA, Body Surface Area; PASI, Psoriasis Area Severity Index; HAQ, Health Assessment Questionnaire; PtGA, patient global assessment; PGA, Physician Global Assessment; VAS, Visual Analogue Scale; WPAI, Work Productivity and Activity Impairment questionnaire.
