# Supplementary material for: Ketogenic diet improves disease activity and cardiovascular risk in psoriatic arthritis: A proof of concept study
Source: PLoS One. 2025 Apr 22;20(4):e0321140. doi: 10.1371/journal.pone.0321140 (PMC12013891; doi:10.1371/journal.pone.0321140)
Supplement: S14 Table — (PDF) [file pone.0321140.s014.pdf]

Table S14. Correlation between the modification of anthropometric measurements and the modification of inflammatory biomarkers during the study.

	Weight		BMI		Abdominal circumference	
	**Spearman's r_s**	**p***	**Spearman's r_s**	**p***	**Spearman's r_s**	**p***
hsCRP	-0.290	0.216	-0.278	0.236	-0.497	0.046
ESR	-0.143	0.547	-0.214	0.365	-0.055	0.816
TNFα	0.153	0.533	0.181	0.459	0.071	0.772

* Significance refers to the Spearman correlation test, indicated by the coefficient r_s.
BMI, Body Mass Index; hsCRP, High Sensitivity C Reactive Protein, ESR, Erythrocyte Sedimentation Rate; TNFα, Tumor Necrosis Factor alpha.
