# Supplementary material for: Ketogenic diet improves disease activity and cardiovascular risk in psoriatic arthritis: A proof of concept study
Source: PLoS One. 2025 Apr 22;20(4):e0321140. doi: 10.1371/journal.pone.0321140 (PMC12013891; doi:10.1371/journal.pone.0321140)
Supplement: S15 Table — (PDF) [file pone.0321140.s015.pdf]

Table S15. Correlation between the modification of anthropometric measurements and the modification of laboratory variables during the study.

	Weight		BMI		Abdominal circumference	
	**Spearman's r_S**	**p***	**Spearman's r_S**	**p***	**Spearman's r_S**	**p***
Total cholesterol	0.133	0.576	0.154	0.516	-0.089	0.708
HDL cholesterol	0.399	0.082	0.259	0.270	0.443	0.050
LDL cholesterol	-0.096	0.686	-0.038	0.875	-0.216	0.361
Triglyceride	-0.252	0.284	-0.068	0.777	-0.566	0.009
AST	0.151	0.524	0.131	0.582	0.114	0.631
ALT	-0.063	0.794	-0.090	0.705	-0.157	0.510
GGT	0.066	0.781	0.071	0.767	-0.125	0.600
TSH	0.094	0.693	0.023	0.922	-0.038	0.875
Creatinine	0.099	0.679	0.058	0.808	-0.129	0.589
Uricemia	-0.547	0.013	-0.437	0.054	-0.367	0.112
Blood glucose	0.602	0.005	0.629	0.003	0.267	0.256
Insulinemia	0.274	0.257	0.136	0.188	0.192	0.432
HOMA-IR	0.451	0.053	0.463	0.046	0.200	0.413
Azotemia	0.248	0.292	0.175	0.461	0.151	0.524
Blood count						
WBC	0.334	0.150	0.448	0.048	0.252	0.284
RBC	0.300	0.199	0.317	0.173	0.007	0.977
Hb	0.287	0.219	0.356	0.123	-0.014	0.953
Hc	0.397	0.083	0.411	0.072	0.034	0.886
MCV	0.223	0.345	0.164	0.490	-0.058	0.808
MCH	-0.163	0.492	-0.158	0.506	0.031	0.897
MCHC	-0.334	0.150	-0.230	0.329	-0.033	0.890
RDW	-0.127	0.595	-0.025	0.917	-0.231	0.327
Platelet	0.005	0.982	0.077	0.748	0.378	0.100
Neutrophils	0.487	0.029	0.583	0.007	0.207	0.382
Lymphocytes	-0.002	0.995	0.090	0.705	-0.001	0.996
Monocytes	0.184	0.437	0.311	0.181	0.112	0.639
Eosinophilic	-0.344	0.138	-0.267	0.255	-0.010	0.967
Basophil	-0.221	0.349	-0.150	0.527	-0.225	0.341
Protein profile						
Total proteins	0.416	0.068	0.371	0.107	0.038	0.872
Albumin	-0.087	0.714	-0.062	0.794	0.038	0.872
α1-globulin	-0.010	0.967	-0.072	0.764	0.024	0.921
α2-globulin	0.052	0.828	0.044	0.855	0.001	0.997
β1-globulin	0.151	0.524	0.136	0.569	0.293	0.210
β2-globulin	-0.252	0.284	-0.249	0.290	-0.390	0.089
γ-globulin	-0.096	0.687	-0.054	0.820	-0.297	0.203
Urine test						
pH	-0.100	0.684	-0.048	0.845	-0.007	0.976
Protein	0.299	0.213	0.174	0.475	0.449	0.054
Hb	0.303	0.208	0.386	0.102	-0.272	0.259
Ketones	-0.564	0.012	-0.512	0.025	-0.381	0.108
Urobilinogen	0.172	0.481	0.172	0.481	0.173	0.480
Specific weight	0.083	0.737	0.107	0.662	-0.075	0.760
Intestinal permeability test						
Diuresis 6h	0.542	0.013	0.545	0.013	0.261	0.267
Lactulose	0.108	0.650	0.113	0.636	0.300	0.199
Mannitol	0.540	0.014	0.498	0.026	0.288	0.218
Sucrose	-0.024	0.920	0.053	0.826	0.059	0.805
Lactulose/mannitol ratio	-0.439	0.053	-0.324	0.163	-0.193	0.416

* Significance refers to the Spearman correlation test, indicated by the coefficient r_S.
BMI, Body Mass Index; HDL, High Density Lipoprotein; LDL, Low Density Lipoprotein; ALT, alanine aminotransferase; AST, aspartate aminotransferase; GGT, gamma glutamyl transpeptidase; TSH, thyroid-stimulating hormone; HOMA-IR, Homeostatic Model Assessment for Insulin Resistance; WBC, white blood cells; RBC, red blood cells; Hb; hemoglobin; MCV, mean corpuscular volume; MCH, mean corpuscular hemoglobin; MCHC, mean corpuscular hemoglobin concentration; RDW, red cell distribution width.
