# Supplementary material for: Ketogenic diet improves disease activity and cardiovascular risk in psoriatic arthritis: A proof of concept study
Source: PLoS One. 2025 Apr 22;20(4):e0321140. doi: 10.1371/journal.pone.0321140 (PMC12013891; doi:10.1371/journal.pone.0321140)
Supplement: S16 Table — (PDF) [file pone.0321140.s016.pdf]

Table S16. Correlation between the modification of anthropometric measurements and the modification of food frequencies during the study.

	Weight		BMI		Abdominal circumference	
	**Spearman's r_s**	**p***	**Spearman's r_s**	**p***	**Spearman's r_s**	**p***
Food Frequency Questionnaire						
Cereals and derivatives	0.320	0.168	0.219	0.354	0.509	0.022
Bakery products	-0.069	0.774	-0.003	0.990	-0.108	0.652
Fresh meat	0.044	0.855	0.147	0.536	-0.017	0.942
Processed meat	0.163	0.493	0.093	0.696	0.515	0.020
Seafood and related products	0.003	0.990	-0.011	0.965	0.124	0.601
Milk and yoghurt	0.073	0.760	0.138	0.561	0.226	0.338
Dairy products	0.075	0.752	-0.019	0.937	0.115	0.630
Fresh fruit	-0.031	0.898	0.004	0.987	0.115	0.629
Nuts	-0.353	0.127	-0.387	0.092	-0.204	0.388
Vegetables	0.087	0.715	0.035	0.884	0.189	0.424
Legumes	-0.443	0.049	-0.409	0.074	-0.349	0.132
Eggs	0.060	0.800	-0.054	0.820	0.182	0.444
Sweets	-0.085	0.720	-0.185	0.434	-0.321	0.168
Soda	0.099	0.678	-0.050	0.835	0.304	0.192
Alcoholic beverages	0.094	0.693	0.011	0.964	0.094	0.695
Physical activity	0.000	1.000	0.040	0.932	0.162	0.728
PREDIMED score	-0.109	0.649	-0.118	0.619	-0.100	0.675

* Significance refers to the Spearman correlation test, indicated by the coefficient r_s.
PREDIMED, PREvención con DIeta MEDiterránea; BMI,
