# Supplementary material for: Ketogenic diet improves disease activity and cardiovascular risk in psoriatic arthritis: A proof of concept study
Source: PLoS One. 2025 Apr 22;20(4):e0321140. doi: 10.1371/journal.pone.0321140 (PMC12013891; doi:10.1371/journal.pone.0321140)
Supplement: S17 Table — (PDF) [file pone.0321140.s017.pdf]

Table S17. Correlation between the modification of anthropometric measurements and the modification of cardiovascular parameters during the study.

	Weight		BMI		Abdominal circumference	
	**Spearman's r_s**	**p***	**Spearman's r_s**	**p***	**Spearman's r_s**	**p***
CUORE§	0.173	0.479	0.216	0.375	-0.318	0.184
SCORE2¤^	-0.034	0.889	0.034	0.889	-0.336	0.159
SBP	0.515	0.020	0.529	0.017	0.105	0.660
DBP	0.481	0.032	0.404	0.077	0.100	0.676

* Significance refers to the Spearman correlation test, indicated by the coefficient r_s.

CUORE, cardiovascular unique offer reengineered; SCORE2, systematic coronary risk evaluation; SBP, systolic blood pressure; DBP, diastolic blood pressure.

¤ Computed from 19 subjects. § 10 year risk of cardiovascular events according to the Progetto CUORE estimator. SCORE2-OP (Older People) estimator was used for subjects >70 years. Values were adjusted for subjects with inflammatory arthritis. Probability is expressed as percentage of risk. ^ 10 year risk of cardiovascular events according to the ESC (European Society of Cardiology), SCORE2 (Systematic Coronary Risk Evaluation 2) estimator. Values were adjusted for subjects with inflammatory arthritis. Probability is expressed as percentage of risk.
