# Supplementary material for: Ketogenic diet improves disease activity and cardiovascular risk in psoriatic arthritis: A proof of concept study
Source: PLoS One. 2025 Apr 22;20(4):e0321140. doi: 10.1371/journal.pone.0321140 (PMC12013891; doi:10.1371/journal.pone.0321140)
Supplement: S18 Table — (PDF) [file pone.0321140.s018.pdf]

Table S18. Association between the modification of anthropometric measurements and the modification of categorical variables during the study.

		Δ weight	Δ BMI	Δ abdominal circumference
IL-1α improvement	1	0 (0;0)	-3.1 (-3.1;-3.1)	-10 (-10;-10)
	0	0 (-0.1;0.2)	-3.6 (-4.1;-2.6)	-12 (-14;-10)
IL-1β improvement	1	0 (-0.1;0)	-3.1 (-3.6;-3.1)	-11 (-12;-10)
	0	0 (-0.2;0.3)	-3.6 (-4.7;-2.6)	-13 (-14.5;-9)
IL-6 improvement	1	0.1 (0;0.2)	-4.6 (-5.6;-3.6)	-11.5 (-14.8;-8.3)
	0	0 (-0.1;0.1)	-3.5 (-3.9;-2.7)	-11.8 (-13.9;-10)
Fecal calproctectin improvement	1	0.2 (0.1;0.3)	-5.1 (-5.9;-4.3)	-12.5 (-15.3;-9.8)
	0	0 (-0.1;0.1)	-3.3 (-3.9;-2.6)	-11.8 (-13.9;-10)
Physical activity improvement	1	0 (0;0.1)	-2.6 (-3.1;-2.5)	-10 (-10.5;-9)
	0	0 (-0.1;0.1)	-3.6 (-4.3;-3.1)	-13 (-14;-10)
PASS improvement	1	n.a.	n.a.	n.a.
	0	0 (-0.1;0.1)	-3.5 (-4;-2.6)	-11.8 (-14;-10)
MDA improvement	1	0.1 (0;0.2)	-2.5 (-2.6;-2.5)	-9 (-9.5;-8.5)
	0	0 (-0.1;0.1)	-3.6 (-4.2;-3.1)	-12.5 (-14;-10.3)
CUORE class improvement §	1	-0.1 (-0.1;-0.1)	-2.6 (-2.6;-2.6)	-5 (-5;-5)
	0	0 (-0.1;0.2)	-3.6 (-4.1;-2.8)	-12 (-14;-10)
SCORE2 class improvement ¤^	1	0 (-0.1;0)	-3.1 (-3.3;-2.9)	-8.3 (-9.9;-6.6)
	0	0 (-0.1;0.2)	-3.5 (-4.2;-2.7)	-12.5 (-14;-10)

For categorical variables “1” refers to “yes”, “0” refers to “no”. Improvement refers to difference between week 0 and week 9. Data are reported as median and interquartile range.

Significance refers to the Kruskal-Wallis test. No association has proven to be statistically significant. ¤ Computed from 19 subjects.

§ 10 year risk of cardiovascular events according to the Progetto CUORE estimator. SCORE2-OP (Older People) estimator was used for subjects >70 years. Values were adjusted for subjects with inflammatory arthritis. Probability is expressed as percentage of risk.^ 10 year risk of cardiovascular events according to the ESC (European Society of Cardiology), SCORE2 (Systematic Coronary Risk Evaluation 2) estimator. Values were adjusted for subjects with inflammatory arthritis. Probability is expressed as percentage of risk.

BMI, Body Mass Index; hsCRP, High Sensitivity C Reactive Protein; IL, interleukin; PASS, Patient Acceptable Symptom State; MDA, Minimal Disease Activity; CUORE, cardiovascular unique offer reengineered; SCORE2, systematic coronary risk evaluation.
