# Supplementary material for: Ketogenic diet improves disease activity and cardiovascular risk in psoriatic arthritis: A proof of concept study
Source: PLoS One. 2025 Apr 22;20(4):e0321140. doi: 10.1371/journal.pone.0321140 (PMC12013891; doi:10.1371/journal.pone.0321140)
Supplement: S20 Table — (PDF) [file pone.0321140.s020.pdf]

Table S20. Analysis of the association between categorical variables at W0 and the modification of continuous clinical variables during the study.

	Gender		Smoke ever		Higher education		Employed		bDMARDs		Axial involvement		Comorbidities		Metabolic syndrome		Cardiovascular comorbidities		W0 elevated IL-1β		W0 elevated IL-6		W0 elevated fecal calprotectin		W0 MDA		W0 PASS		W0 physical activity°	
	**1**	**0**	**1**	**0**	**1**	**0**	**1**	**0**	**1**	**0**	**1**	**0**	**1**	**0**	**1**	**0**	**1**	**0**	**1**	**0**	**1**	**0**	**1**	**0**	**1**	**0**	**1**	**0**	**1**	**0**
Δ TJC (0-68)	-2 (-2;0)	-1 (-2;0)	-1.5 (-2;-0.3)	-1 (-2.8;0)	-2 (-3;0)	0 (-1;0)	-1.5 (-2.3;0)	0 (-0.3;0.5)	-2 (-2.5;-0.5)	0 (-1;0)	-1 (-2;0)	-1.5 (-4.8;0)	-1 (-2;0)	-1 (-3;0)	-1 (-1.8;0)	-1.5 (-2;0)	-1 (-2;0)	-1 (-3;0)	-2 (-2.3;-1.8)	-1 (-2;0)	-1.5 (-4.8;-0.8)	-1 (-2;0)	-1 (-1;0)	-2 (-2;0)	0 (-1;0)	-2 (-2.5;-0.5)	-0.5 (-1.8;0)	-1.5 (-2;-0.3)	-0.5 (-1;0)	-2 (-2.8;-0.3)
Δ SJC (0-68)	0 (-1;0)	0 (-1;0)	0 (-1.5;0)	0 (-0.8;0)	0 (-2;0)	0 (0;0)	0 (-2;0)	0 (-0.3;0)	0 (-2;0)	0 (0;0)	0 (0;0)	-1 (-2.8;0)	0 (-1.5;0)	0 (0;0)	0 (0;0)	0 (-2;0)	0 (-1;0)	0 (-1;0)	-1 (-3;0)	0 (-0.5;0)	-3.5 (-5.3;-1.5)	0 (0;0)	0 (-0.5;0)	0 (-2;0)	0 (0;0)	0 (-2;0)	0 (-0.8;0)	0 (-1.5;0)	0 (0;0)	0 (-1.8;0)
Δ TJC (0-28)	0 (-2;0)	0 (-1;0)	-1.5 (-2;-0.3)	0 (-0.8;0)	0 (-2;0)	0 (-0.5;0)	-0.5 (-2;0)	0 (0;0)	0 (-2;0)	0 (-1;0)	0 (-1;0)	-0.5 (-2.8;0)	0 (-1;0)	0 (-2;0)	0 (-0.8;0)	-0.5 (-2;0)	0 (-1.5;0)	0 (-1;0)	-1 (-2;0)	0 (-1;0)	-1.5 (-2.8;-0.8)	0 (-0.5;0)	0 (-1;0)	0 (-2;0)	0 (-1;0)	0 (-2;0)	0 (-0.8;0)	-0.5 (-2;0)	0 (-0.8;0)	-1 (-2;0)
Δ SJC (0-28)	0 (0;0)	0 (0;0)	0 (-0.8;0)	0 (0;0)	0 (0;0)	0 (0;0)	0 (0;0)	0 (0;0)	0 (0;0)	0 (0;0)	0 (0;0)	0 (-2;0)	0 (0;0)	0 (0;0)	0 (0;0)	0 (-0.8;0)	0 (0;0)	0 (0;0)	0 (-1.5;0)	0 (0;0)	-3 (-5.3;-0.8)	0 (0;0)	0 (0;0)	0 (0;0)	0 (0;0)	0 (-0.5;0)	0 (0;0)	0 (-0.8;0)	0 (0;0)	0 (0;0)
Δ Dactylitis	0 (0;0)	0 (0;0)	0 (0;0)	0 (0;0)	0 (0;0)	0 (0;0)	0 (0;0)	0 (0;0)	0 (0;0)	0 (0;0)	0 (0;0)	0 (0;0)	0 (0;0)	0 (0;0)	0 (0;0)	0 (0;0)	0 (0;0)	0 (0;0)	0 (0;0)	0 (0;0)	0 (0;0)	0 (0;0)	0 (0;0)	0 (0;0)	0 (0;0)	0 (0;0)	0 (0;0)	0 (0;0)	0 (0;0)	0 (0;0)
Δ DAPSA	-5.9 (-9.2;9.9)	-8 (-20.4;-0.1)	-7.1 (-14;-1.1)	-3.9 (-16.7;5.2)	-5.9 (-9.2;0.7)	-10 (-20.4;7.5)	-6.1 (-16.8;1.2)	-1 (-12.9;9)	-8 (-17.5;-3.9)	2.9 (-10;9.9)	-7.1 (-16.8;0.1)	-2.6 (-14;10.4)	-5.9 (-20.4;4.4)	-6.2 (-9.2;0.7)	1 (-5.2;7.4)	-12.6 (-26.7;-6.5)	-16 (-23.7;-1.5)	-1.9 (-9.2;5.9)	-8.1 (-14.6;-3.2)	-1.9 (-12.6;5.4)	-18.2 (-28.6;-5.9)	-5.9 (-13;6.9)	2.9 (-5.4;8.9)	-8 (-19;-1.9)	2.9 (-5.9;9.9)	-16 (-25;-4.1)	4.4 (-4.7;9.4)	-17.5 (-26.7;-7.2)	-1.4 (-7.5;2.4)	-9.6 (-25.3;2.9)
Δ HAQ	0.3 (-0.6;0.6)	-0.3 (-0.9;0.2)	-0.2 (-0.7;0.1)	0.2 (-0.8;0.6)	-0.1 (-0.6;0.5)	0.1 (-0.8;0.3)	-0.2 (-0.8;0.4)	0.2 (-0.1;0.5)	-0.4 (-0.9;0.1)	0.3 (0.1;0.6)	-0.2 (-0.8;0.3)	0.3 (-0.7;0.6)	-0.6 (-0.9;0.4)	0.1 (-0.3;0.6)	0.3 (-0.1;0.5)	-0.7 (-0.9;0.1)	-0.8 (-1;-0.2)	0.2 (-0.3;0.6)	0 (-0.3;0.2)	0.2 (-0.8;0.5)	-0.6 (-0.9;-0.2)	0.1 (-0.7;0.6)	0.3 (0.2;0.6)	-0.4 (-0.8;0.2)	0.4 (0.2;0.6)	-0.8 (-1;0)	0.5 (0.2;0.6)	-0.8 (-1.1;-0.2)	-0.1 (-0.6;0.3)	0 (-0.9;0.6)
Δ DAS28-CRP	-0.3 (-1;0.6)	-1.5 (-2;0.4)	-0.5 (-1.5;0.7)	-1 (-2;0.4)	-0.9 (-1.7;-0.1)	-1.5 (-1.9;2.6)	-0.9 (-1.8;0.2)	-0.4 (-1.7;1.3)	-1 (-1.9;-0.1)	-0.3 (-1.5;2.2)	-1 (-1.5;0.1)	-0.7 (-2.4;1.3)	-1.1 (-2.2;1)	-0.9 (-1.5;-0.1)	0.3 (-1;2)	-1.9 (-3.1;-0.5)	-1.5 (-2.7;1.4)	-0.9 (-1.5;0.6)	-1.2 (-1.9;-0.3)	-0.3 (-1.6;0.8)	-2.5 (-3.3;-1)	-0.9 (-1.5;0.3)	-0.3 (-1.3;1.7)	-1 (-2.1;-0.1)	-0.3 (-1.1;2.2)	-1.5 (-2.8;-0.1)	0.1 (-1.1;2)	-1.5 (-3.1;-0.3)	-0.7 (-1.6;0.7)	-0.9 (-2.8;0.4)
Δ CDAI	-2 (-8;7)	-8 (-14;2)	-7.5 (-14;-0.3)	-1.5 (-8.8;6)	-2 (-8;2)	-8 (-14;5)	-4.5 (-10;2.3)	-1 (-11.5;7.5)	-8 (-12;0)	3 (-9;7)	-7.5 (-10.8;2.3)	0 (-11;7)	-2 (-17.5;5)	-7 (-8;2)	2.5 (-5.5;8.5)	-12 (-19.8;-3.5)	-8 (-22;0.5)	-1 (-8;7)	-8 (-13.8;-2.8)	-1 (-8;5)	-14 (-22;-5.5)	-2 (-8.5;7)	3 (-5;7)	-8 (-16;2)	3 (-2;7)	-9 (-19.5;-2.5)	5 (-1.8;8.5)	-12.5 (-19.8;-7.3)	0.5 (-6.5;2.8)	-8.5 (-19;5)
Δ SDAI	-1.9 (-8.2;7)	-8 (-13.8;1.9)	-7.1 (-13.3;0.4)	-1.4 (-8.8;5.9)	-1.9 (-8.2;2.7)	-8 (-13.8;5)	-4.1 (-9.9;2.7)	-1 (-11.4;7.5)	-8 (-11.6;-0.4)	2.9 (-9;7)	-7.1 (-10.5;1.5)	0.4 (-11.3;6.9)	-1.9 (-16.9;5)	-6.2 (-8.2;2.7)	2 (-4.9;8.5)	-11.6 (-20;-3.5)	-8 (-21.7;0.5)	-0.9 (-8.2;6.9)	-7.6 (-13.8;-2.2)	-0.9 (-8.1;4.9)	-14.3 (-22.4;-5.4)	-1.9 (-8.6;6.9)	2.9 (-4.9;6.9)	-8 (-15;1.1)	2.9 (-1.9;7)	-9 (-19.6;-2.6)	4.9 (-1.7;8.5)	-12 (-20;-6.7)	0.1 (-6.5;2.9)	-8.6 (-19.1;5.2)
Δ SPARCC	0 (-1;6)	-2 (-7;0)	0 (-0.8;4.5)	-1 (-5.8;1.5)	0 (-2;2)	-5 (-7;3)	0 (-2.3;2.5)	-4.5 (-6.5;-0.8)	0 (-2;1)	-2 (-5;4)	-3 (-6.5;-0.8)	5 (0;6.5)	-3 (-6.5;0)	2 (0;6)	-1.5 (-4.5;1.5)	0 (-2.3;3)	-3 (-7.5;0)	0 (-2;4)	-0.5 (-2.3;0)	0 (-3;5)	0 (0;1.5)	-1 (-4.5;3)	-2 (-4;2)	0 (-3;2)	0 (-2;6)	-1 (-7;0)	0 (-1.5;5.5)	-2 (-7.5;0)	0 (-4.3;3.5)	-0.5 (-3;0)
Δ BSA	0 (-1;1)	0 (-0.5;0)	0 (-0.8;0.8)	0 (-0.8;0)	0 (-1;0)	0 (0;0)	0 (-1;0.3)	0 (-1.5;0)	0 (-0.5;0.5)	0 (-1;0)	0 (-0.3;0)	0 (-2.3;1.3)	0 (-0.5;0)	0 (-1;0)	0 (-0.8;0)	0 (-0.8;0.8)	0 (-1;0.5)	0 (-1;0)	0 (-0.3;0)	0 (-1;0.5)	0 (-12.3;0.3)	0 (-1;0)	0 (-3.5;0)	0 (0;1)	0 (-1;0)	0 (-0.5;0)	0 (-0.8;0)	0 (-0.8;0)	0 (0;0)	0 (-1;0)
Δ PASI	-0.4 (-3;0.6)	0 (-0.5;0)	0.5 (-0.4;1.2)	0 (-1.6;0)	-0.4 (-1.8;0)	0 (0;0.3)	0 (-1.2;0.2)	0 (-1;0)	0 (-1.2;0.5)	0 (-1;0)	0 (-0.1;0)	-1.7 (-3.3;0.8)	0 (-1.4;0)	0 (-0.5;0)	0 (-0.4;0)	-0.2 (-2.7;0.8)	0 (-2.4;0.5)	0 (-0.5;0)	0 (-0.1;0.3)	0 (-2.4;0)	0.6 (-1.8;1.4)	0 (-1.4;0)	-0.4 (-2.5;0)	0 (-0.5;0.6)	-0.4 (-3;0)	0 (-0.3;0.5)	-0.2 (-2.5;0)	0 (-0.4;0.8)	0 (-0.9;0)	0 (-1.5;0.5)

Δ LEI	0 (-1;4)	0 (-4;0)	0 (-0.8;1.5)	0 (-4;3)	0 (-1;2)	-4 (-5;1)	0 (-1.3;2.5)	-2 (-4.5;0.5)	0 (-1.5;1)	0 (-4;2)	-1 (-4;0)	2 (0;4)	-1 (-4;0)	2 (0;4)	0 (-3.3;1.5)	0 (-1.8;1.5)	-2 (-4;-0.5)	0 (0;4)	-0.5 (-1.8;0)	0 (-1.5;3)	0 (0;0.5)	0 (-3;3)	0 (-2;1)	0 (-2;2)	0 (0;4)	-1 (-3;0)	0 (0;3.5)	-1 (-3.5;0)	0 (-4;2)	0 (-1;0)
Δ PtGA	1 (-4;3)	-1 (-4;0.5)	-4 (-4.8;-0.3)	0 (-2.5;3)	0 (-4;3)	-1 (-5;1)	-0.5 (-4;1.5)	-1 (-4;1.5)	-1 (-4.5;0.5)	1 (-3;3)	-2 (-4.3;0.3)	1 (-1.8;3.5)	-1 (-6;1)	0 (-4;3)	0.5 (-0.8;2.5)	-4 (-6.5;0.5)	-1 (-6;0)	0 (-4;3)	-3.5 (-5;-1.3)	0 (-2.5;2)	-4 (-5;-2.8)	0 (-3.5;3)	1 (-1.5;2)	-1 (-5;1)	1 (0;3)	-4 (-6;-0.5)	2 (0.3;3.8)	-4 (-6.5;-1.5)	0 (-1;1)	-3.5 (-4.8;3)
Δ PGA	1 (-1;3)	-1 (-4;0.5)	-2.5 (-4.8;0.5)	0 (-3.3;2.8)	0 (-4;2)	-1 (-5.5;1)	-0.5 (-4;1.3)	-1.5 (-4.8;1.5)	-1 (-4.5;0.5)	1 (-4;2)	-1 (-4.3;0.3)	1 (-1.8;2.3)	-1 (-5;1)	0 (-4;2)	0.5 (-0.8;2.5)	-4 (-5;0.5)	-1 (-5;0)	0 (-4;3)	-2.5 (-5;0.3)	0 (-2.5;1.5)	-4 (-5;-2.8)	0 (-2.5;2.5)	1 (-2;1.5)	-1 (-5;1)	1 (0;3)	-4 (-5;-0.5)	1.5 (0.3;3)	-4 (-5;-1)	0 (-1;1)	-2.5 (-4.8;3)
Δ VAS pain	2 (-1;4)	-2 (-4.5;0.5)	-2.5 (-5.5;0.5)	0 (-3.5;3.8)	0 (-4;3)	-2 (-6;1.5)	-0.5 (-4.3;1.8)	-1 (-5;2.3)	-1 (-5;0.5)	1 (-4;3)	-1.5 (-4.5;0.3)	1.5 (-2;4.3)	-1 (-6.5;1.5)	0 (-4;4)	0.5 (-0.8;2.8)	-4.5 (-6.8;0.5)	-2 (-6.5;0)	0 (-4;4)	-2.5 (-5;0.3)	0 (-3;2.5)	-4.5 (-5.8;-2.8)	0 (-3;3.5)	1 (-2;2.5)	-1 (-6;1)	2 (0;4)	-4 (-6.5;-0.5)	2.5 (0.3;4)	-4.5 (-6.8;-1.3)	0 (-1.8;1)	-2.5 (-5.8;3.8)
Δ ASDAS-CRP	-0.1 (-0.5;0)	-0.4 (-0.7;-0.3)	-0.2 (-0.9;0)	-0.4 (-0.6;-0.1)	-0.4 (-0.5;0)	-0.2 (-1;-0.1)	-0.4 (-0.7;-0.1)	-0.1 (-0.6;0)	-0.5 (-1;-0.2)	-0.2 (-0.4;0)	-0.4 (-0.9;0)	-0.3 (-0.4;-0.1)	-0.4 (-0.9;0)	-0.2 (-0.4;-0.1)	-0.3 (-0.6;-0.1)	-0.4 (-1;0)	-0.5 (-0.9;0)	-0.3 (-0.4;-0.1)	-0.7 (-1.2;-0.3)	-0.2 (-0.5;0.1)	-0.4 (-0.6;-0.2)	-0.3 (-0.7;-0.1)	-0.1 (-0.3;0.1)	-0.5 (-1.1;-0.2)	0 (-0.2;0.2)	-0.6 (-1.1;-0.4)	-0.1 (-0.3;0.2)	-0.7 (-1.1;-0.4)	-0.4 (-0.6;0)	-0.3 (-0.9;-0.1)
Δ BASDAI	-0.5 (-1.2;0)	-1.7 (-1.8;-0.8)	-1.3 (-2.3;-0.2)	-1 (-1.8;-0.3)	-1 (-1.8;-0.2)	-0.5 (-2.4;-0.3)	-1.1 (-1.8;-0.4)	-0.3 (-1.4;0)	-1.7 (-2.2;-0.8)	-0.5 (-1;0)	-1 (-2;-0.3)	-0.8 (-1.8;-0.1)	-1 (-2.2;-0.3)	-0.5 (-1.8;-0.2)	-0.8 (-1.5;-0.1)	-1.5 (-2.5;-0.3)	-1.2 (-2.2;-0.3)	-1 (-1.8;-0.2)	-1.8 (-2.7;-0.9)	-0.7 (-1.8;0.1)	-1.8 (-2.2;-1)	-1 (-1.8;-0.3)	-0.2 (-0.8;0.1)	-1.7 (-2.5;-0.7)	0 (-0.5;0.8)	-1.8 (-2.6;-1.3)	-0.1 (-0.9;0.7)	-1.8 (-2.6;-1.7)	-1.1 (-1.8;0)	-0.8 (-2.3;-0.5)
Δ WPAI lost work hours	0 (0;0)	0 (-2;0)	0 (0;0)	0 (0;0)	0 (0;0)	0 (0;0)	0 (0;0)	0 (0;0)	0 (-2;0)	0 (0;0)	0 (0;0)	0 (0;0)	0 (0;0)	0 (0;0)	0 (0;0)	0 (0;0)	0 (-2;0)	0 (0;0)	0 (0;0)	0 (0;0)	0 (-1.3;0)	0 (0;0)	0 (0;0)	0 (0;0)	0 (0;0)	0 (-2;0)	0 (0;0)	0 (-3;0)	0 (0;0)	0 (0;0)
Δ WPAI impact	0 (-2;1)	0 (-3;0.5)	-2.5 (-3.8;-1.3)	0 (0;1.8)	0 (-3;1)	0 (-1.5;0.5)	0 (-3.3;1.3)	0 (-0.8;0.3)	-1 (-3.5;0)	0 (0;1)	0 (-3;0.3)	0 (-2.3;2)	0 (-2.5;0.5)	0 (-3;2)	0.5 (0;1.8)	-2.5 (-5.3;-0.3)	0 (-4;0)	0 (-3;2)	-2 (-4.5;1.5)	0 (-1.5;1)	-4.5 (-12.3;-2.5)	0 (-1;1.5)	0 (0;1)	-1 (-3;0)	0 (0;1)	-2 (-5;0)	0.5 (0;4)	-2.5 (-5.5;-0.3)	0 (-0.8;1)	-1 (-5.5;0)

Gender “1” refers to male, “0” refers to female; for the other variables “1” refers to “yes”, “0” refers to “no”.
Δ refers to difference between week 0 and week 9.
Data are reported as median and interquartile range.
Significant associations are indicated by green cells. Significance refers to the Kruskal-Wallis test.
° Weekly, according to the Food Frequency Questionnaire.
W0, week 0; bDMARDs, biological disease-modifying antirheumatic drugs; IL, interleukin; MDA, Minimal Disease Activity; PASS, Patient Acceptable Symptom State; TJC, tender joint count; SJC, swollen joint count; DAPSA, disease activity index in psoriatic arthritis; HAQ, Health Assessment Questionnaire; DAS28-CRP, disease activity score on 28 joints with C reactive protein; CDAI, clinical disease activity index; SDAI, Simple Disease Activity Index; SPARCC, Spondylarthritis Research Consortium of Canada; BSA, Body Surface Area; PASI, Psoriasis Area Severity Index; LEI, Leeds Enthesitis Index; PtGA, patient global assessment; PGA, Physician Global Assessment; VAS, Visual Analogue Scale; ASDAS-CRP, Ankylosing Spondylitis Disease Activity Score – C Reactive Protein; BASDAI, Bath Ankylosing Spondylitis Disease Activity Index; WPAI, Work Productivity and Activity Impairment questionnaire.
