# Supplementary material for: Ketogenic diet improves disease activity and cardiovascular risk in psoriatic arthritis: A proof of concept study
Source: PLoS One. 2025 Apr 22;20(4):e0321140. doi: 10.1371/journal.pone.0321140 (PMC12013891; doi:10.1371/journal.pone.0321140)
Supplement: S22 Table — (PDF) [file pone.0321140.s022.pdf]

Table S22. Analysis of the association between categorical variables at W0 and the modification of continuous laboratory variables during the study.

	Gender		Smoke ever		Higher education		Employed		bDMARDs		Axial involvement		Comorbidities		Metabolic syndrome		Cardiovascular comorbidities		W0 elevated IL-1β		W0 elevated IL-6		W0 elevated fecal calprotectin		W0 MDA		W0 PASS		W0 physical activity°	
	1	0	1	0	1	0	1	0	1	0	1	0	1	0	1	0	1	0	1	0	1	0	1	0	1	0	1	0	1	0
Laboratory variables																														
Δ Total cholesterol	-5 (-19;2)	-36 (-43;-23)	-12.5 (-24.5;-5.8)	-33 (-42.5;-5.5)	-19 (-40;-8)	-30 (-46;-3)	-23 (-40.3;-4)	-34 (-57.8;-1.3)	-19 (-35;-6.5)	-36 (-48;-1)	-28.5 (-41.5;-18.5)	-6.5 (-42;-0.3)	-30 (-42;-1.5)	-19 (-40;-8)	-33 (-42.5;-19)	-6.5 (-36.8;2.8)	-30 (-43;-1.5)	-19 (-40;-8)	-29 (-43;-14)	-27 (-41.5;-4.5)	-17.5 (-32.3;-7.3)	-19 (-40.5;0.5)	-43 (-48.5;-27.5)	-17 (-30;-1)	-27 (-40;-1)	-19 (-42;-6.5)	-31.5 (-40.8;-5.5)	-18 (-39.8;-5.8)	-23 (-34.5;-0.5)	-29.5 (-42.5;-8)
Δ HDL cholesterol	-3 (-4;3)	-7 (-14.5;1.5)	-6 (-11.8;-3.3)	-2 (-8.5;5)	-8 (-13;-1)	1 (-3.5;9.5)	-3.5 (-10;3.8)	-3.5 (-5.5;-2)	-7 (-11;0)	-1 (-4;2)	-5.5 (-9.3;2.3)	-3 (-7;2.5)	1 (-6.5;4.5)	-7 (-13;-3)	-3.5 (-7.8;5)	-3.5 (-12;0.5)	3 (-3.5;10.5)	-7 (-13;-1)	-6 (-11.3;-4)	-3 (-9.5;4.5)	-14.5 (-21.5;-10.8)	-3 (-7.5;2.5)	-3 (-7;0.5)	-4 (-9;3)	-1 (-3;6)	-7 (-12.5;-1.5)	-2 (-8.3;5)	-5.5 (-8.8;-0.3)	0 (-6;5)	-6 (-9.8;-3.3)
Δ LDL cholesterol	-6 (-12;-1)	-23 (-33.5;-8.5)	-2 (-7.5;11)	-17.5 (-33.8;-7.3)	-11 (-27.6;-3)	-8 (-40;-3.5)	-8.5 (-28.2;-2.5)	-24.5 (-45.5;6.3)	-8 (-17;-2)	-27.6 (-43;-6)	-11.5 (-31.8;-6.8)	-6.5 (-29.5;4)	-12 (-36.5;-4)	-9 (-27.6;-3)	-17.5 (-41.5;-8.8)	-4 (-23;18)	-8 (-36.5;-4)	-11 (-27.6;-3)	-13 (-26.5;-2.5)	-11 (-32.5;-6.5)	-5 (-13.7;5.3)	-11 (-32.5;-4.5)	-37 (-57;-19.8)	-7 (-11;-1)	-12 (-43;-7)	-8 (-25.3;7)	-17.5 (-41;-7.5)	-5.5 (-23.5;11)	-8.5 (-35;12.5)	-17.5 (-29.4;-3.8)
Δ Triglyceride	-49 (-54;3)	-21 (-28;-8.5)	-35.5 (-52.8;1.3)	-24.5 (-47;-7.3)	-19 (-41;3)	-49 (-62;-24.5)	-20.5 (-50.3;4.5)	-38.5 (-54;-26.3)	-22 (-47.5;6)	-28 (-49;-11)	-28 (-60.8;-20.5)	-4 (-43;10.3)	-41 (-63.5;-17)	-19 (-22;3)	-49 (-65.3;-19.5)	-16.5 (-28;7.5)	-49 (-66.5;-34.5)	-19 (-28;3)	-6 (-29.3;11.3)	-28 (-53.5;-8.5)	-1 (-13.8;16.3)	-28 (-51.5;-12.5)	-21 (-59;-8.5)	-28 (-49;3)	-41 (-49;-6)	-21 (-41;-1)	-31.5 (-49;0.8)	-24.5 (-47.5;-13)	-25 (-47;-9.3)	-24.5 (-52.8;-0.5)
Δ AST	-1 (-4;-1)	-2 (-6;1.5)	-2 (-3.8;-1)	-1.5 (-6.3;2.5)	-1 (-4;3)	-3 (-6;-2)	-1 (-4.8;2.3)	-3 (-5;-1.8)	-1 (-2.5;5)	-4 (-8;-1)	-1 (-2;1.3)	-5.5 (-12.5;-1.5)	-1 (-3.5;0.5)	-2 (-12;2)	-1 (-2;0.8)	-3.5 (-7.8;1.3)	-2 (-3.5;0)	-1 (-8;2)	-1.5 (-2.3;2.5)	-1 (-5.5;1.5)	-7.5 (-12.5;-1.8)	-1 (-4;1.5)	-2 (-5.5;-0.5)	-1 (-4;3)	0 (-4;2)	-2 (-6;-1)	0.5 (-3.3;2.8)	-2.5 (-7;-1.3)	-1 (-6.3;1.8)	-1.5 (-3.8;-1)
Δ ALT	-1 (-14;7)	2 (-4.5;8.5)	0.5 (-10.8;5.8)	-0.5 (-8.3;9.8)	2 (-3;10)	-6 (-20.5;3)	0.5 (-6.8;8.3)	-3.5 (-17.3;7.8)	2 (-4.5;7.5)	-3 (-19;9)	4.5 (-3;8.3)	-11.5 (-19.5;1.8)	-3 (-7.5;4.5)	7 (-19;10)	2 (-5.3;8.5)	-2 (-16.5;7.8)	-3 (-7.5;3)	2 (-19;10)	2 (1.3;3.3)	-3 (-11.5;9.5)	3.5 (-6;8.5)	-3 (-11.5;7)	7 (-8.5;9.5)	-1 (-9;7)	-3 (-14;9)	2 (-4.5;7)	-0.5 (-12.8;8.8)	0.5 (-5.3;7)	-7.5 (-17.8;5.3)	4.5 (-0.3;9.3)
Δ GGT	-18 (-22;-11)	-7 (-12.5;-4.5)	-10 (-15.5;-4.5)	-12.5 (-22;-6.3)	-11 (-22;-7)	-8 (-41;-4)	-10 (-22;-6)	-14 (-43;-8.8)	-9 (-22;-5)	-14 (-18;-6)	-8.5 (-14;-6)	-17.5 (-22;-11.3)	-9 (-19.5;-6.5)	-14 (-22;-3)	-7.5 (-15.5;-6)	-16 (-22;-9.5)	-9 (-19.5;-7)	-11 (-22;-6)	-4.5 (-11.5;-1.8)	-11 (-20;-7)	-7 (-11.8;-2.5)	-17 (-22.5;-7.5)	-11 (-15.5;-6)	-11 (-23;-7)	-17 (-22;-11)	-8 (-18.5;-2.5)	-14 (-21;-8)	-8.5 (-20.8;-2.3)	-9.5 (-17.8;-6)	-12.5 (-22.8;-7.5)
Δ TSH	0.1 (0.1;0.6)	0.3 (0;0.8)	0.1 (-0.1;0.4)	0.2 (0.1;0.9)	0.1 (0.1;0.9)	0.1 (-0.1;0.4)	0.1 (0;0.8)	0.2 (0;0.6)	0.4 (0.1;0.8)	0.1 (-0.2;0.3)	0.2 (0.1;1)	0.1 (-0.2;0.6)	0.6 (0.1;1.2)	0.1 (-0.3;0.3)	0.3 (0;1.3)	0.1 (0.1;0.4)	0.1 (0;0.7)	0.1 (0.1;0.7)	0.4 (0.2;0.9)	0.1 (-0.1;0.8)	0.1 (-0.4;0.6)	0.1 (0.1;0.9)	0.1 (-0.1;0.9)	0.1 (0.1;0.7)	0.1 (-0.2;0.9)	0.3 (0.1;0.6)	0.1 (-0.2;1.3)	0.2 (0.1;0.5)	0.3 (0;0.8)	0.1 (0.1;0.5)
Δ Creatinine	0 (-0.1;0.1)	0.1 (0;0.1)	0 (0;0.1)	0 (0;0.1)	0 (-0.1;0.1)	0.1 (0;0.1)	0 (-0.1;0.1)	0.1 (0.1;0.1)	0 (-0.1;0.1)	0.1 (0;0.1)	0 (0;0.1)	0 (0;0.1)	0.1 (0;0.1)	0 (0;0)	0 (0;0.1)	0 (-0.1;0.1)	0 (-0.1;0.1)	0 (0;0.1)	0 (0;0.1)	0 (-0.1;0.1)	0.1 (0.1;0.1)	0 (-0.1;0.1)	0.1 (0;0.1)	0 (-0.1;0.1)	0 (0;0.1)	0 (0;0.1)	0 (0;0.1)	0 (0;0.1)	0 (0;0.1)	0 (-0.1;0.1)
Δ Uricemia	0 (0;0)	0 (0;0.1)	0 (0;0.1)	0 (-0.1;0)	0 (0;0.1)	0 (-0.1;0)	0 (0;0)	0 (0;0.1)	0 (0;0)	0 (-0.1;0)	0 (0;0)	0 (0;0.1)	0 (0;0)	0 (0;0.2)	0 (-0.1;0)	0 (0;0.1)	0 (0;0)	0 (0;0.1)	0 (0;0)	0 (-0.1;0.1)	0.2 (0.1;0.2)	0 (-0.1;0)	0 (-0.1;0.1)	0 (0;0)	0 (-0.1;0)	0 (0;0.1)	0 (-0.1;0.1)	0 (0;0)	0 (-0.1;0)	0 (0;0.1)
Δ Blood glucose	-9 (-16;2)	-7 (-13.5;4.5)	-15 (-17.5;-8.8)	-3.5 (-9;5.8)	-7 (-14;4)	-7 (-18;-1.5)	-6 (-16.3;4.8)	-8 (-19;-4.8)	-9 (-16.5;0)	-5 (-9;7)	-4.5 (-9;4.8)	-16.5 (-25.8;-3.3)	-7 (-12.5;3)	-7 (-17;2)	-3.5 (-12.8;5.8)	-8 (-16.8;-0.3)	-9 (-18;3)	-7 (-14;2)	-8 (-14.5;-1)	-7 (-17.5;5.5)	-17 (-34;-13.8)	-5 (-11.5;3)	2 (-29;8.5)	-7 (-16;-2)	-5 (-9;7)	-9 (-17;0)	-3.5 (-8.5;5.8)	-11.5 (-17.5;-0.3)	-2.5 (-15.3;9.3)	-9 (-15.5;-2.8)
Δ Insulinemia	-8.6 (-12.5;-5.1)	-4.6 (-7.9;-1.2)	-6.4 (-11.5;-3.4)	-5.5 (-8.9;-3.9)	-5.4 (-10.2;-4.2)	-7.8 (-8.6;-3.4)	-5.5 (-10;-4.1)	-5.5 (-8.4;-2.9)	-7.9 (-12.7;-4.8)	-4.6 (-7.8;-3.1)	-7.9 (-9.5;-1.8)	-4.9 (-8.4;-4.1)	-7.9 (-9.7;-5.5)	-4.2 (-5.1;-2.4)	-5.8 (-10;-2.6)	-5.5 (-8.4;-4.7)	-7.9 (-8.9;-5.5)	-4.6 (-10.2;-2.4)	-12.7 (-13.9;-10)	-5.1 (-8.3;-3.4)	-4.4 (-7.8;-3.2)	-5.6 (-9.4;-3.4)	-4.6 (-7.2;-2.8)	-7.8 (-12.5;-4.2)	-5.1 (-9.2;-3.1)	-7.8 (-10.6;-4.4)	-5.3 (-10;-3.2)	-6.7 (-8.4;-4.3)	-4.7 (-7.2;-0.8)	-9.4 (-12.7;-4.9)
Δ Azotemia	-0.9 (-1.6;1.3)	1.2 (-0.6;1.5)	0.9 (-0.5;1.3)	0 (-1.3;1.6)	0.6 (-1.2;1.9)	0.5 (-0.9;1.4)	0 (-1.3;1.5)	1.4 (0.8;1.5)	0.6 (-1.1;1.5)	0.5 (-0.8;1.4)	1.4 (0.6;1.9)	-1.3 (-1.6;-0.9)	1.3 (-0.7;2)	-0.7 (-1.4;1.2)	1.4 (0.8;1.8)	-0.9 (-1.5;-0.6)	0.5 (-0.7;1.5)	0.6 (-1.2;1.4)	1 (0.2;1.6)	0.5 (-1.3;1.5)	-0.8 (-1;-0.2)	1.3 (-1.1;1.8)	1.3 (-0.1;1.7)	-0.5 (-1.2;1.4)	0.5 (-1.6;1.3)	0.6 (-0.8;1.5)	0.9 (-1.6;1.8)	0 (-0.8;1.4)	0.9 (-1.1;1.4)	0 (-0.9;1.8)
Δ Total proteins	-0.6 (-0.7;-0.2)	-0.9 (-1.2;0)	-0.6 (-1.4;-0.3)	-0.7 (-1;0.2)	-0.6 (-0.9;0.3)	-0.7 (-1.3;-0.3)	-0.8 (-1.3;-0.1)	-0.3 (-0.4;0)	-0.9 (-1.4;-0.3)	-0.3 (-0.7;0.3)	-0.7 (-1.1;-0.3)	-0.5 (-0.9;0.8)	-0.9 (-1.4;0)	-0.4 (-0.7;-0.2)	-0.5 (-0.8;-0.3)	-0.8 (-1.4;0.8)	-1.2 (-1.5;-0.7)	-0.4 (-0.7;0.3)	-0.6 (-1;-0.4)	-0.7 (-1.2;0)	-1.2 (-2.1;-0.5)	-0.4 (-0.8;0)	-0.3 (-0.8;0)	-0.7 (-1.2;-0.2)	-0.6 (-0.7;0.3)	-0.9 (-1.5;-0.3)	-0.7 (-0.8;0.2)	-0.8 (-1.5;-0.3)	0 (-0.7;0.8)	-0.8 (-1.4;-0.6)
Δ Albumin	-0.2 (-0.4;0.1)	0.1 (0;0.2)	0 (-0.2;0.1)	0.1 (-0.1;0.2)	0 (-0.2;0.1)	0.1 (-0.2;0.1)	0 (-0.1;0.1)	0 (-0.3;0.2)	0 (-0.1;0.1)	0.1 (-0.2;0.2)	0.1 (0;0.2)	-0.2 (-0.3;0.1)	0 (-0.3;0.2)	0.1 (-0.1;0.1)	0.1 (0;0.1)	0 (-0.2;0.1)	-0.1 (-0.3;0.1)	0.1 (-0.1;0.2)	0.1 (-0.1;0.2)	0 (-0.2;0.1)	-0.1 (-0.2;0)	0.1 (-0.1;0.2)	0.1 (-0.2;0.2)	0 (-0.1;0.1)	-0.1 (-0.2;0.1)	0.1 (0;0.2)	0 (-0.2;0.1)	0 (0;0.2)	0.1 (-0.1;0.1)	0 (-0.2;0.1)
Δ α1-globulin	-6 (-9;0)	2 (0;5)	-1 (-5;0)	1.5 (-1.8;4)	0 (-2;3)	2 (-4;4)	0 (-2;3.3)	-1 (-8.5;4.5)	0 (-5;0.5)	3 (-1;4)	0.5 (-0.5;4.3)	-3.5 (-8.3;3.3)	0 (-7;3)	0 (-1;4)	1 (-2;4.8)	0 (-6.3;2.5)	-2 (-7;0.5)	3 (-1;5)	1.5 (-5.5;5.3)	0 (-4;2.5)	-0.5 (-4.8;1.3)	0 (-4;4)	2 (-3.5;4.5)	0 (-2;4)	0 (-8;3)	0 (-1.5;4.5)	1 (-7.5;3.8)	0 (-1.8;3.3)	1 (-1.5;3.8)	-0.5 (-7.3;3.3)
Δ α2-globulin	0.7 (-1.4;0.9)	0 (-1.4;1.6)	0.3 (-0.7;0.9)	0.3 (-1.4;1.3)	-0.2 (-1.4;1.3)	0.7 (-0.5;0.8)	0.8 (-1;1.4)	-1.3 (-1.6;-0.6)	-0.2 (-1.5;1.2)	0.7 (-1.1;1.3)	-0.6 (-1.5;1.2)	0.8 (0.3;1.3)	0 (-1.3;1.1)	0.7 (-1.4;1.3)	0.3 (-1.5;0.9)	0.4 (-1.1;1.3)	0.7 (-0.1;1.1)	-0.9 (-1.4;1.3)	-1.6 (-1.7;-0.9)	0.7 (-0.5;1.4)	1.2 (0.5;1.6)	-0.2 (-1.5;1.2)	0.7 (-0.7;1.4)	-0.2 (-1.4;1)	0.7 (0;1.3)	-0.9 (-1.6;1)	0.7 (-1;1.3)	-0.5 (-1.4;1)	1.2 (0.2;1.5)	-1.4 (-1.6;0.5)
Δ β1-globulin	-0.2 (-0.3;0.4)	-0.1 (-0.3;0.2)	0 (-0.3;0.3)	-0.1 (-0.4;0.3)	-0.1 (-0.3;0.5)	-0.1 (-0.4;0.2)	-0.1 (-0.3;0.2)	0 (-0.4;0.4)	-0.1 (-0.4;0.2)	-0.1 (-0.3;0.4)	-0.2 (-0.4;0)	0.3 (-0.3;0.5)	-0.1 (-0.3;0.2)	-0.1 (-0.4;0.4)	0 (-0.2;0.3)	-0.3 (-0.4;0.4)	-0.1 (-0.3;0.2)	-0.1 (-0.4;0.4)	-0.4 (-0.5;-0.2)	0.1 (-0.2;0.4)	0.4 (0.1;0.6)	-0.2 (-0.4;0.2)	-0.1 (-0.3;0.4)	-0.1 (-0.4;0.2)	0.3 (-0.2;0.5)	-0.2 (-0.4;0.1)	0.2 (-0.2;0.5)	-0.3 (-0.4;0.1)	0 (-0.2;0.3)	-0.3 (-0.5;0.3)
Δ β2-globulin	-1 (-4;1)	0 (-3.5;3.5)	-0.5 (-3.3;0.8)	-0.5 (-3.8;3.8)	-1 (-4;4)	0 (-4;1)	-1 (-4.8;1.8)	2 (0.8;5)	-1 (-5.5;2)	1 (-1;3)	-1 (-4;0.8)	1 (-2.5;4)	-1 (-5;2.5)	0 (-4;3)	0 (-3.8;2.5)	-0.5 (-5.5;3)	-1 (-7.5;0)	0 (-3;4)	-0.5 (-4.8;3.8)	-1 (-3.5;2.5)	2 (-1.8;4.3)	-1 (-4;2)	1 (-2;3.5)	-1 (-4;1)	0 (-1;1)	-1 (-5.5;3.5)	0.5 (-1;3.3)	-2.5 (-6.3;2.3)	-0.5 (-3.8;0.8)	0 (-3.3;3.8)
Δ γ-globulin	0.4 (0.2;0.9)	0.2 (-0.2;0.5)	0.4 (0.3;0.6)	0.2 (0;0.5)	0.3 (0.1;0.7)	0.3 (0.2;0.4)	0.4 (0;0.6)	0.2 (0.2;0.2)	0.4 (0.1;0.7)	0.2 (0.2;0.3)	0.2 (0;0.5)	0.4 (0.2;0.6)	0.3 (0.2;0.6)	0.2 (0.1;0.5)	0.2 (0.1;0.4)	0.4 (0.1;0.8)	0.4 (0.1;0.7)	0.2 (0.1;0.5)	0.3 (0.2;0.5)	0.3 (0;0.6)	0.4 (0.2;0.5)	0.3 (0.1;0.6)	0.2 (0;0.3)	0.4 (0.1;0.6)	0.3 (0.2;0.6)	0.2 (-0.1;0.5)	0.4 (0.2;0.7)	0.2 (-0.3;0.4)	0.4 (0.2;0.6)	0.2 (0.1;0.5)
Δ WBC	-14 (-21;-6)	-30 (-48;-25)	-14 (-19.8;-7.5)	-29.5 (-49;-16.3)	-30 (-49;-12)	-23 (-28;-18.5)	-28.5 (-49;-10.8)	-19.5 (-24.5;-15.5)	-21 (-42.5;-9)	-27 (-49;-16)	-28 (-40.8;-14)	-18.5 (-49;-10.8)	-27 (-38;-15)	-23 (-49;-12)	-19.5 (-29.3;-14)	-34 (-49;-14.3)	-21 (-36.5;-11)	-29 (-49;-14)	-22 (-24.8;-13.5)	-27 (-49;-13)	-30 (-44;-18.8)	-23 (-47.5;-10.5)	-27 (-54;-19.5)	-21 (-39;-7)	-27 (-49;-14)	-23 (-38;-13)	-28.5 (-46.5;-14.5)	-22 (-41.8;-12.5)	-28 (-44.3;-14.5)	-22 (-44.3;-8.8)
Δ RBC	-0.2 (-0.2;0)	-0.3 (-0.6;0.2)	-0.2 (-1;0.3)	-0.2 (-0.4;0.1)	-0.2 (-0.7;0.3)	-0.2 (-0.4;0.1)	-0.3 (-0.8;0.2)	0.1 (0;0.1)	-0.3 (-0.8;0)	0 (-0.2;0.1)	-0.3 (-0.5;0.1)	-0.2 (-0.5;0.1)	-0.3 (-0.8;-0.1)	0.1 (-0.2;0.4)	-0.1 (-0.3;0.2)	-0.3 (-1.1;0)	-0.5 (-1;-0.2)	0.1 (-0.2;0.3)	-0.3 (-0.8;0.2)	-0.2 (-0.4;0.1)	-0.8 (-1.5;-0.2)	-0.1 (-0.3;0.2)	0 (-0.3;0.2)	-0.2 (-0.7;0.1)	-0.2 (-0.2;0)	-0.3 (-1;0.2)	-0.2 (-0.4;0)	-0.1 (-1.1;0.2)	0.1 (-0.3;0.3)	-0.5 (-1.1;-0.2)
Δ Hb	-0.3 (-0.4;-0.1)	-0.3 (-0.4;-0.1)	-0.3 (-0.3;-0.1)	-0.3 (-0.4;0)	-0.2 (-0.4;0)	-0.4 (-0.4;-0.2)	-0.3 (-0.4;-0.1)	-0.3 (-0.3;0)	-0.3 (-0.4;0)	-0.3 (-0.4;-0.2)	-0.3 (-0.4;-0.2)	-0.2 (-0.4;0.1)	-0.2 (-0.3;0)	-0.4 (-0.4;-0.3)	-0.3 (-0.4;-0.2)	-0.1 (-0.4;0.1)	-0.3 (-0.7;-0.1)	-0.3 (-0.4;0)	-0.2 (-0.3;-0.1)	-0.3 (-0.4;-0.2)	-0.2 (-0.6;0)	-0.3 (-0.4;-0.1)	-0.3 (-0.4;-0.2)	-0.3 (-0.4;0)	-0.3 (-0.4;-0.2)	-0.3 (-0.7;0)	-0.3 (-0.3;-0.1)	-0.3 (-0.8;-0.1)	-0.2 (-0.3;0.1)	-0.3 (-0.4;-0.2)
Δ MCV	-0.1 (-0.1;0)	-0.1 (-0.2;0)	-0.1 (-0.1;0)	-0.1 (-0.1;0)	-0.1 (-0.1;0)	-0.1 (-0.1;0)	-0.1 (-0.2;0)	0 (0;0)	-0.1 (-0.2;-0.1)	0 (-0.1;0)	-0.1 (-0.2;0)	-0.1 (-0.1;0)	-0.1 (-0.2;0)	-0.1 (-0.1;0)	-0.1 (-0.1;0)	-0.1 (-0.1;0)	-0.2 (-0.2;-0.1)	-0.1 (-0.1;0)	-0.1 (-0.1;0)	-0.1 (-0.2;0)	-0.1 (-0.1;0)	-0.1 (-0.1;0)	0 (-0.1;0)	-0.1 (-0.2;0)	-0.1 (-0.1;0)	-0.1 (-0.1;0)	-0.1 (-0.1;0)	-0.1 (-0.1;0)	0 (-0.1;0)	-0.1 (-0.1;-0.1)
Δ MCH	0 (0;0)	-0.1 (-0.1;0)	-0.1 (-0.1;0)	0 (-0.1;0)	0 (-0.1;0)	-0.1 (-0.1;0)	0 (-0.1;0)	0 (0;0)	0 (-0.1;0)	0 (-0.1;0)	-0.1 (-0.1;0)	0 (0;0.1)	-0.1 (-0.1;0)	0 (0;0)	0 (-0.1;0)	0 (-0.1;0.1)	-0.1 (-0.1;0)	0 (-0.1;0)	-0.1 (-0.1;0)	0 (-0.1;0)	0 (-0.1;0.1)	0 (-0.1;0)	0 (-0.1;0)	0 (-0.1;0)	0 (-0.1;0)	0 (-0.1;0)	0 (-0.1;0)	0 (-0.1;0)	0 (-0.1;0)	0 (-0.1;0)
Δ MCHC	0 (0;0)	0 (0;0)	0 (0;0)	0 (0;0)	0 (0;0)	0 (0;0)	0 (0;0)	0 (0;0)	0 (0;0)	0 (0;0)	0 (0;0)	0 (0;0)	0 (0;0)	0 (0;0)	0 (0;0)	0 (0;0)	0 (0;0)	0 (0;0)	0 (0;0)	0 (0;0)	0 (0;0)	0 (0;0)	0 (0;0)	0 (0;0)	0 (0;0)	0 (0;0)	0 (0;0)	0 (0;0)	0 (0;0)	0 (0;0)
Δ RDW	0.4 (0.2;0.9)	0.2 (-0.2;0.5)	0.4 (0.3;0.6)	0.2 (0;0.5)	0.3 (0.1;0.7)	0.3 (0.2;0.4)	0.4 (0;0.6)	0.2 (0.2;0.2)	0.4 (0.1;0.7)	0.2 (0.2;0.3)	0.2 (0;0.5)	0.4 (0.2;0.6)	0.3 (0.2;0.6)	0.2 (0.1;0.5)	0.2 (0.1;0.4)	0.4 (0.1;0.8)	0.4 (0.1;0.7)	0.2 (0.1;0.5)	0.3 (0.2;0.5)	0.3 (0;0.6)	0.4 (0.2;0.5)	0.3 (0.1;0.6)	0.2 (0;0.3)	0.4 (0.1;0.6)	0.3 (0.2;0.6)	0.2 (-0.1;0.5)	0.4 (0.2;0.7)	0.2 (-0.3;0.4)	0.4 (0.2;0.6)	0.2 (0.1;0.5)
Δ Platelet	-14 (-21;-6)	-30 (-48;-25)	-14 (-19.8;-7.5)	-29.5 (-49;-16.3)	-30 (-49;-12)	-23 (-28;-18.5)	-28.5 (-49;-10.8)	-19.5 (-24.5;-15.5)	-21 (-42.5;-9)	-27 (-49;-16)	-28 (-40.8;-14)	-18.5 (-49;-10.8)	-27 (-38;-15)	-23 (-49;-12)	-19.5 (-29.3;-14)	-34 (-49;-14.3)	-21 (-36.5;-11)	-29 (-49;-14)	-22 (-24.8;-13.5)	-27 (-49;-13)	-30 (-44;-18.8)	-23 (-47.5;-10.5)	-27 (-54;-19.5)	-21 (-39;-7)	-27 (-49;-14)	-23 (-38;-13)	-28.5 (-46.5;-14.5)	-22 (-41.8;-12.5)	-28 (-44.3;-14.5)	-22 (-44.3;-8.8)
Δ Neutrophils	-0.2 (-0.2;0)	-0.3 (-0.6;0.2)	-0.2 (-1;0.3)	-0.2 (-0.4;0.1)	-0.2 (-0.7;0.3)	-0.2 (-0.4;0.1)	-0.3 (-0.8;0.2)	0.1 (0;0.1)	-0.3 (-0.8;0)	0 (-0.2;0.1)	-0.3 (-0.5;0.1)	-0.2 (-0.5;0.1)	-0.3 (-0.8;-0.1)	0.1 (-0.2;0.4)	-0.1 (-0.3;0.2)	-0.3 (-1.1;0)	-0.5 (-1;-0.2)	0.1 (-0.2;0.3)	-0.3 (-0.8;0.2)	-0.2 (-0.4;0.1)	-0.8 (-1.5;-0.2)	-0.1 (-0.3;0.2)	0 (-0.3;0.2)	-0.2 (-0.7;0.1)	-0.2 (-0.2;0)	-0.3 (-1;0.2)	-0.2 (-0.4;0)	-0.1 (-1.1;0.2)	0.1 (-0.3;0.3)	-0.5 (-1.1;-0.2)
Δ Lymphocytes	-0.3 (-0.4;-0.1)	-0.3 (-0.4;-0.1)	-0.3 (-0.3;-0.1)	-0.3 (-0.4;0)	-0.2 (-0.4;0)	-0.4 (-0.4;-0.2)	-0.3 (-0.4;-0.1)	-0.3 (-0.3;0)	-0.3 (-0.4;0)	-0.3 (-0.4;-0.2)	-0.3 (-0.4;-0.2)	-0.2 (-0.4;0.1)	-0.2 (-0.3;0)	-0.4 (-0.4;-0.3)	-0.3 (-0.4;-0.2)	-0.1 (-0.4;0.1)	-0.3 (-0.7;-0.1)	-0.3 (-0.4;0)	-0.2 (-0.3;-0.1)	-0.3 (-0.4;-0.2)	-0.2 (-0.6;0)	-0.3 (-0.4;-0.1)	-0.3 (-0.4;-0.2)	-0.3 (-0.4;0)	-0.3 (-0.4;-0.2)	-0.3 (-0.7;0)	-0.3 (-0.3;-0.1)	-0.3 (-0.8;-0.1)	-0.2 (-0.3;0.1)	-0.3 (-0.4;-0.2)
Δ Monocytes	-0.1 (-0.1;0)	-0.1 (-0.2;0)	-0.1 (-0.1;0)	-0.1 (-0.1;0)	-0.1 (-0.1;0)	-0.1 (-0.1;0)	-0.1 (-0.2;0)	0 (0;0)	-0.1 (-0.2;-0.1)	0 (-0.1;0)	-0.1 (-0.2;0)	-0.1 (-0.1;0)	-0.1 (-0.2;0)	-0.1 (-0.1;0)	-0.1 (-0.1;0)	-0.1 (-0.1;0)	-0.2 (-0.2;-0.1)	-0.1 (-0.1;0)	-0.1 (-0.1;0)	-0.1 (-0.2;0)	-0.1 (-0.1;0)	-0.1 (-0.1;0)	0 (-0.1;0)	-0.1 (-0.2;0)	-0.1 (-0.1;0)	-0.1 (-0.1;0)	-0.1 (-0.1;0)	-0.1 (-0.1;0)	0 (-0.1;0)	-0.1 (-0.1;-0.1)
Δ Eosinophilic	0 (0;0)	-0.1 (-0.1;0)	-0.1 (-0.1;0)	0 (-0.1;0)	0 (-0.1;0)	-0.1 (-0.1;0)	0 (-0.1;0)	0 (0;0)	0 (-0.1;0)	0 (-0.1;0)	-0.1 (-0.1;0)	0 (0;0.1)	-0.1 (-0.1;0)	0 (0;0)	0 (-0.1;0)	0 (-0.1;0.1)	-0.1 (-0.1;0)	0 (-0.1;0)	-0.1 (-0.1;0)	0 (-0.1;0)	0 (-0.1;0.1)	0 (-0.1;0)	0 (-0.1;0)	0 (-0.1;0)	0 (-0.1;0)	0 (-0.1;0)	0 (-0.1;0)	0 (-0.1;0)	0 (-0.1;0)	0 (-0.1;0)

Δ Basophil	0 (0;0)	0 (0;0)	0 (0;0)	0 (0;0)	0 (0;0)	0 (0;0)	0 (0;0)	0 (0;0)	0 (0;0)	0 (0;0)	0 (0;0)	0 (0;0)	0 (0;0)	0 (0;0)	0 (0;0)	0 (0;0)	0 (0;0)	0 (0;0)	0 (0;0)	0 (0;0)	0 (0;0)	0 (0;0)	0 (0;0)	0 (0;0)	0 (0;0)	0 (0;0)	0 (0;0)	0 (0;0)	0 (0;0)	0 (0;0)
Urine test																														
Δ pH	0 (0;0.5)	0 (-0.3;0.5)	0 (-0.4;0)	0.3 (0;0.5)	0 (0;0.5)	0 (-0.3;0.3)	0.3 (-0.1;0.5)	0 (0;0)	0 (-0.5;0.5)	0 (0;0.5)	0 (0;0.5)	0.3 (-0.1;0.5)	0 (0;0.5)	0 (0;0.5)	0 (0;0.4)	0.3 (0;0.5)	0 (-0.3;0.5)	0 (0;0.5)	0 (-0.5;0.1)	0 (0;0.5)	0 (-0.9;0.5)	0 (0;0.5)	0 (0;0.5)	0 (-0.5;0.5)	0.5 (0;0.5)	0 (-0.5;0.3)	0.5 (0;0.5)	0 (-0.5;0)	0 (-0.4;0.5)	0 (0;0.5)
Δ Protein	0 (0;0)	0 (0;0)	0 (0;0)	0 (0;0)	0 (0;0)	0 (0;0)	0 (0;0)	0 (0;0)	0 (0;0)	0 (0;0)	0 (0;0)	0 (0;0)	0 (0;0)	0 (0;0)	0 (0;0)	0 (0;0)	0 (0;0)	0 (0;0)	0 (0;0)	0 (0;0)	0 (0;0)	0 (0;0)	0 (0;0)	0 (0;0)	0 (0;0)	0 (0;0)	0 (0;0)	0 (0;0)	0 (0;0)	0 (0;0)
Δ Hb	0 (0;0)	0 (0;0)	0 (0;0)	0 (0;0)	0 (0;0)	0 (0;0)	0 (0;0)	0 (0;0)	0 (0;0)	0 (0;0)	0 (0;0)	0 (0;0)	0 (0;0)	0 (0;0)	0 (0;0)	0 (0;0)	0 (0;0)	0 (0;0)	0 (0;0)	0 (0;0)	0 (0;0)	0 (0;0)	0 (0;0)	0 (0;0)	0 (0;0)	0 (0;0)	0 (0;0)	0 (0;0)	0 (0;0)	0 (0;0)
Δ Ketones	0 (0;0.1)	0 (0;0)	0.1 (0;0.1)	0 (0;0)	0 (0;0.1)	0 (0;0)	0 (0;0)	0.1 (0;0.1)	0 (0;0)	0 (0;0.1)	0 (0;0)	0 (0;0.1)	0 (0;0.1)	0 (0;0)	0 (0;0)	0 (0;0.1)	0 (0;0.1)	0 (0;0)	0 (0;0)	0 (0;0.1)	0.1 (0;0.2)	0 (0;0)	0 (0;0.1)	0 (0;0)	0 (0;0)	0 (0;0.1)	0 (0;0)	0 (0;0.1)	0 (0;0)	0 (0;0.1)
Δ Urobilinogen	0 (0;0)	0 (0;0)	0 (0;0)	0 (0;0)	0 (0;0)	0 (0;0)	0 (0;0)	0 (0;0)	0 (0;0)	0 (0;0)	0 (0;0)	0 (0;0)	0 (0;0)	0 (0;0)	0 (0;0)	0 (0;0)	0 (0;0)	0 (0;0)	0 (-16.2;0)	0 (0;0)	0 (-16.2;0)	0 (0;0)	0 (0;0)	0 (0;0)	0 (0;0)	0 (0;0)	0 (0;0)	0 (0;0)	0 (0;0)	0 (0;0)
Δ Specific weight	-1 (-6;1)	-4 (-8;5.5)	-6 (-9.8;0.8)	-2.5 (-5.8;4)	-4 (-7;2)	-3 (-5.5;2)	-3.5 (-7.5;1.3)	-0.5 (-6.3;5.8)	-4 (-8;-1.5)	1 (-6;6)	-4.5 (-7.5;2.8)	-0.5 (-4;2)	-4 (-6.5;-0.5)	0 (-9;5)	-2.5 (-5.5;5)	-4 (-7;0.8)	-4 (-6;-0.5)	-1 (-7;5)	-3.5 (-5.8;-0.3)	-1 (-6.5;3.5)	-6 (-9.3;-0.8)	-1 (-5.5;3.5)	5 (-2.5;6)	-4 (-7;-1)	-1 (-7;1)	-4 (-6;3.5)	-2.5 (-6.8;0.8)	-3.5 (-6.5;4.3)	-5.5 (-8.5;4)	-2 (-4.8;1.5)
Intestinal permeability test																														
Δ Diuresis 6h	90 (5;90)	-60 (-115;105)	17.5 (1.3;127.5)	20 (-67.5;90)	50 (-60;160)	5 (-80;60)	40 (-62.5;170)	-27.5 (-70;26.3)	0 (-65;70)	90 (-60;220)	25 (-62.5;170)	17.5 (-77.5;90)	30 (-30;90)	-10 (-100;160)	47.5 (-55;172.5)	15 (-60;80)	30 (2.5;70)	-10 (-70;160)	-20 (-77.5;72.5)	50 (-35;125)	-50 (-192.5;62.5)	5 (-60;90)	90 (-47.5;255)	0 (-60;90)	90 (90;220)	-60 (-115;15)	90 (26.3;205)	-35 (-122.5;22.5)	47.5 (-47.5;205)	15 (-67.5;80)
Δ Lactulose	0.1 (0;0.2)	0 (-0.1;0)	0 (-0.1;0.1)	0 (0;0.2)	0 (-0.1;0.1)	0 (0;0.2)	0 (-0.1;0.1)	0.1 (0;0.1)	0 (-0.1;0)	0 (0;0.2)	0 (0;0)	0.1 (-0.1;0.2)	0 (0;0.1)	0 (-0.1;0.1)	0 (0;0.2)	0 (-0.1;0)	0 (0;0.1)	0 (-0.1;0.1)	0 (0;0.1)	0 (-0.1;0.2)	-0.1 (-0.1;0)	0 (0;0.2)	0 (0;0.2)	0 (0;0.1)	0.2 (0;0.2)	0 (-0.1;0)	0.1 (0;0.2)	0 (-0.1;0)	0 (-0.1;0.1)	0 (0;0.1)
Δ Mannitol	-2.7 (-8.2;-1.3)	-4 (-8.2;6)	-7.3 (-8.1;-4.6)	-1.6 (-7.7;5.3)	-2.7 (-6.7;5.1)	-8.2 (-12.1;-0.2)	-3.3 (-8.1;0.5)	-3.8 (-10.3;1.7)	-5.1 (-8.2;-1.7)	-1.1 (-8.2;5.1)	-0.4 (-5.5;5.7)	-8 (-16.2;-2.5)	-6.7 (-12.1;5.2)	-2.1 (-4;-1.1)	-0.2 (-4.2;6.3)	-7.3 (-16.3;-3)	-8.2 (-12.1;-0.7)	-2.1 (-5.1;0.6)	-3.2 (-7.7;-0.8)	-2.7 (-8;5.2)	-11.7 (-16.5;-6.9)	-2.1 (-7.5;2.9)	0.6 (-5.5;5.9)	-5.1 (-8.6;-1.3)	-2.1 (-4;5.1)	-6.7 (-12.1;-0.4)	-2.4 (-4.8;3.6)	-7.3 (-13.8;0.1)	-5.9 (-8.5;4.3)	-1.7 (-6.3;0.2)

Δ refers to difference between week 0 and week 9.

Significant associations are indicated by green cells. Significance refers to the Kruskal-Wallis test.

¤ Computed from 19 subjects.

§ 10 year risk of cardiovascular events according to the Progetto estimator. SCORE2-OP (Older People) estimator was used for subjects >70 years. Values were adjusted for subjects with inflammatory arthritis. Probability is expressed as percentage of risk.

^ 10 year risk of cardiovascular events according to the ESC (European Society of Cardiology), SCORE2 (Systematic Coronary Risk Evaluation 2) estimator. Values were adjusted for subjects with inflammatory arthritis. Probability is expressed as percentage of risk.

The subsequent baseline variables were excluded from the analysis of the study group due to inadequate case number: elevated IL-1α, fibromyalgia, uveitis, inflammatory bowel disease, HLA-B27.

W0, week 0; IL, interleukin; MDA, Minimal Disease Activity; PASS, Patient Acceptable Symptom State; BMI, Body Mass Index; hsCRP, High Sensitivity C Reactive Protein; ESR, Erythrocyte Sedimentation Rate; TNFα, Tumor Necrosis Factor alpha; CUORE, cardiovascular unique offer reengineered; SCORE2, systematic coronary risk evaluation; SBP, systolic blood pressure; DBP, diastolic blood pressure; PREDIMED, PREvención con DIeta MEDiterránea; TJC, tender joint count; SJC, swollen joint count; DAPSA, disease activity index in psoriatic arthritis; HAQ, Health Assessment Questionnaire; DAS28-CRP, disease activity score on 28 joints with C reactive protein; CDAI, clinical disease activity index; SDAI, Simple Disease Activity Index; BSA, Body Surface Area; PASI, Psoriasis Area Severity Index; LEI, Leeds Enthesitis Index; SPARCC, Spondylarthritis Research Consortium of Canada; VAS, Visual Analogue Scale; PtGA, patient global assessment; PGA, Physician Global Assessment; WPAI, Work Productivity and activity Impairment questionnaire; BASDAI, Bath Ankylosing Spondylitis Disease Activity Index; ASDAS-CRP, Ankylosing Spondylitis Disease Activity Score – C Reactive Protein; HDL, High Density Lipoprotein; LDL, Low Density Lipoprotein; ALT, alanine aminotransferase; AST, aspartate aminotransferase; GGT, gamma glutamyl transpeptidase; TSH, thyroid-stimulating hormone; WBC, white blood cells; RBC, red blood cells; Hb; hemoglobin; MCV, mean corpuscular volume; MCH, mean corpuscular hemoglobin; MCHC, mean corpuscular hemoglobin concentration; RDW, red cell distribution width.
