# Supplementary material for: Ketogenic diet improves disease activity and cardiovascular risk in psoriatic arthritis: A proof of concept study
Source: PLoS One. 2025 Apr 22;20(4):e0321140. doi: 10.1371/journal.pone.0321140 (PMC12013891; doi:10.1371/journal.pone.0321140)
Supplement: S23 Table — (PDF) [file pone.0321140.s023.pdf]

Table S23. Analysis of the association between categorical variables at W0 and the modification of nutritional questionnaires during the study.

	**Gender**		**Smoke ever**		**Higher education**		**Employed**		**bDMARDs**		**Axial involvement**		**Comorbidities**		**Metabolic syndrome**		**Cardiovascular comorbidities**		**W0 elevated IL-1β**		**W0 elevated IL-6**		**W0 elevated fecal calprotectin**		**W0 MDA**		**W0 PASS**		**W0 physical activity°**	
	**1**	**0**	**1**	**0**	**1**	**0**	**1**	**0**	**1**	**0**	**1**	**0**	**1**	**0**	**1**	**0**	**1**	**0**	**1**	**0**	**1**	**0**	**1**	**0**	**1**	**0**	**1**	**0**	**1**	**0**
Food Frequency Questionnaire																														
Δ Cereals and by-products	-1 (-1;-0.4)	-0.4 (-1;-0.3)	-0.6 (-0.9;-0.4)	-0.7 (-1;-0.3)	-0.7 (-1;-0.4)	-0.3 (-1;-0.2)	-0.9 (-1;-0.4)	-0.3 (-0.5;-0.2)	-0.6 (-1;-0.4)	-1 (-1;-0.3)	-0.4 (-0.8;-0.3)	-1 (-1.5;-0.9)	-0.4 (-1;-0.4)	-1 (-1;-0.3)	-0.4 (-1;-0.3)	-0.9 (-1;-0.5)	-0.4 (-0.9;-0.4)	-1 (-1;-0.3)	-0.6 (-1.1;-0.3)	-0.7 (-1;-0.4)	-1 (-1.5;-0.9)	-0.4 (-1;-0.3)	-1 (-2;-0.4)	-0.6 (-1;-0.3)	-1 (-1;-0.4)	-0.4 (-1;-0.3)	-1 (-1;-0.5)	-0.4 (-0.9;-0.3)	-0.4 (-0.9;-0.3)	-1 (-1;-0.5)
Δ Bakery products	-0.3 (-0.9;0)	-1 (-1;-0.6)	-0.8 (-0.9;-0.4)	-0.9 (-1;0)	-0.4 (-0.9;-0.1)	-1 (-1;-0.9)	-0.8 (-1;-0.1)	-0.9 (-0.9;-0.6)	-0.8 (-1;-0.2)	-0.9 (-1;0)	-0.9 (-1;-0.4)	-0.5 (-1;-0.1)	-0.9 (-1;-0.9)	-0.3 (-0.8;-0.1)	-0.9 (-1;-0.3)	-0.8 (-0.9;-0.1)	-0.9 (-1;-0.9)	-0.4 (-1;-0.1)	-1 (-1;-0.8)	-0.8 (-1;-0.1)	-0.9 (-1;-0.7)	-0.9 (-1;-0.1)	-1 (-1;-0.4)	-0.8 (-0.9;-0.1)	-0.8 (-0.9;0)	-0.9 (-1;-0.4)	-0.8 (-1;0)	-0.9 (-1;-0.3)	-0.9 (-1;-0.5)	-0.6 (-1;0)
Δ Fresh meat	0.1 (0;0.7)	-0.1 (-0.3;0.1)	0.1 (-0.1;0.6)	0 (-0.2;0.4)	0 (-0.2;0.4)	0.1 (-0.6;0.5)	0 (-0.2;0.5)	0.1 (0.1;0.3)	0 (-0.2;0.8)	0.1 (-0.2;0.2)	-0.1 (-0.2;0.3)	0.1 (-0.1;0.5)	0.1 (-0.2;0.4)	0 (-0.2;0.4)	-0.1 (-0.4;0.1)	0.1 (-0.1;0.7)	0.1 (-0.4;0.8)	0 (-0.2;0.2)	0.4 (0;0.9)	0 (-0.3;0.3)	-0.1 (-0.2;0.2)	0.1 (-0.1;0.6)	0.1 (-0.1;0.3)	0 (-0.2;0.7)	0 (-0.4;0.2)	0 (-0.1;0.8)	-0.1 (-0.4;0.1)	0.1 (-0.1;0.8)	0.1 (-0.4;0.4)	0 (-0.1;0.5)
Δ Processed meat	0 (-0.4;0)	0.1 (-0.1;0.3)	-0.2 (-0.4;0)	0.1 (0;0.4)	0 (-0.4;0.1)	0.1 (0;0.3)	0 (-0.2;0.2)	0.1 (-0.2;0.3)	0 (-0.2;0.6)	0 (0;0.1)	0.1 (-0.2;0.2)	0 (-0.2;0.2)	0.1 (-0.1;0.6)	0 (-0.4;0)	0.1 (0;0.8)	-0.1 (-0.2;0)	0.1 (-0.1;0.6)	0 (-0.4;0.1)	-0.1 (-0.3;0.4)	0 (-0.1;0.3)	-0.2 (-0.3;-0.1)	0 (-0.1;0.6)	0 (-0.4;0.1)	0 (-0.1;0.4)	0 (-0.4;0.1)	0 (-0.1;0.3)	0.1 (-0.3;0.7)	0 (-0.1;0.1)	0.1 (-0.1;0.8)	0 (-0.2;0)
Δ Fish and other fishery products	0.1 (0;0.4)	0.1 (0;0.3)	0.1 (-0.1;0.3)	0.1 (0;0.4)	0.1 (0;0.4)	0 (0;0.3)	0.1 (0;0.4)	0.1 (0;0.2)	0.1 (0;0.4)	0.2 (0;0.3)	0.1 (0;0.4)	0.2 (0;0.3)	0.1 (0;0.4)	0.1 (0;0.3)	0.2 (0.1;0.4)	0.1 (0;0.2)	0.1 (0;0.4)	0.1 (0;0.3)	0 (0;0.1)	0.2 (0.1;0.4)	0 (-0.1;0.2)	0.1 (0;0.4)	0.2 (0.1;0.3)	0.1 (0;0.4)	0.2 (0;0.4)	0.1 (0;0.3)	0.2 (0;0.4)	0.1 (0;0.2)	0.1 (0;0.3)	0.2 (0.1;0.4)
Δ Milk and yoghurt	0.7 (0;1)	0 (-0.3;0.9)	1 (-0.5;1.5)	0 (-0.2;0.8)	0.3 (0;0.9)	0 (-0.6;1)	0.5 (0;1)	-0.6 (-1;0.3)	0.7 (-0.2;1)	0 (-0.3;1)	0.1 (-0.5;0.9)	0.4 (0;1)	0 (-0.7;0.9)	0.7 (0;1)	0.9 (0.1;1)	0 (-0.8;0.5)	0 (-0.2;1.3)	0.3 (-0.3;0.9)	0.3 (-0.5;0.9)	0.3 (0;1)	-0.5 (-1;0.3)	0.3 (-0.1;1)	0 (-0.1;0.6)	0.7 (-0.3;1)	0.3 (0;1)	0 (-0.3;1)	0.5 (0;1)	0 (-0.3;1)	0.1 (0;1)	0.4 (-0.3;1)
Δ Dairy products	-0.4 (-0.6;0)	-0.1 (-0.3;-0.1)	-0.2 (-0.4;0)	-0.3 (-0.5;0)	-0.3 (-0.6;0)	-0.1 (-0.3;-0.1)	-0.1 (-0.4;0)	-0.4 (-0.5;-0.3)	-0.1 (-0.4;0)	-0.3 (-0.4;-0.1)	-0.2 (-0.4;0)	-0.3 (-0.6;-0.1)	-0.1 (-0.4;0)	-0.3 (-0.4;-0.1)	-0.2 (-0.3;0)	-0.3 (-0.5;0)	-0.1 (-0.2;0)	-0.3 (-0.4;-0.1)	-0.3 (-0.3;-0.2)	-0.1 (-0.5;0)	-0.2 (-0.3;-0.1)	-0.3 (-0.5;0)	-0.3 (-0.5;-0.2)	-0.1 (-0.4;0)	-0.4 (-0.6;-0.1)	-0.1 (-0.3;0)	-0.4 (-0.6;-0.2)	-0.1 (-0.3;0)	-0.2 (-0.4;-0.1)	-0.3 (-0.4;0)
Δ Fresh fruit	-1 (-1;-0.7)	-1 (-1.8;-0.9)	-0.9 (-1.8;-0.5)	-1 (-1.5;-0.9)	-1 (-1.5;-0.7)	-1 (-1.5;-1)	-1 (-1.6;-0.6)	-1 (-1.4;-1)	-1 (-1.8;-0.6)	-1 (-1.5;-1)	-1 (-1.6;-0.7)	-1 (-1.3;-0.7)	-1 (-1.8;-0.9)	-1 (-1;-0.4)	-1 (-1.4;-0.9)	-1 (-2;-0.7)	-1 (-1.5;-0.9)	-1 (-1.5;-0.7)	-1.3 (-1.6;-0.9)	-1 (-1.3;-0.7)	-1.4 (-2.3;-0.6)	-1 (-1.5;-0.8)	-1 (-1.3;-1)	-1 (-2;-0.4)	-1 (-1;-0.7)	-1.5 (-2;-0.9)	-1 (-1;-0.8)	-1.5 (-2;-0.8)	-1 (-1.4;-0.8)	-1 (-1.9;-0.5)
Δ Nuts	0.6 (0;1.9)	0.9 (0.6;1)	1.4 (0.7;1.9)	0.7 (0;1)	0.9 (0;1)	0.6 (0.4;1.2)	0.9 (0.5;1.2)	0.3 (-0.1;0.9)	1 (0.7;1.9)	0.6 (0;0.8)	0.8 (0.4;1)	0.8 (0.1;1.9)	0.8 (0.3;1)	0.9 (0.2;1.9)	0.8 (0.6;1.6)	0.8 (0;1)	1 (0.6;1.4)	0.8 (0;1)	0.7 (0.6;1.1)	0.9 (0.1;1.4)	0.8 (0.4;1.3)	0.9 (0.1;1.4)	0.6 (0.3;0.9)	0.9 (0.2;1.9)	0.2 (0;0.8)	1 (0.7;1.4)	0.4 (0;0.8)	1 (0.7;1.6)	0.7 (0;1.6)	0.9 (0.6;1)
Δ Vegetables	1 (-0.9;1.4)	0 (-0.4;0.5)	0.7 (0.1;1.3)	0 (-1;1)	0 (-0.9;1)	1 (0.2;1.4)	0.2 (-0.3;1.1)	-0.4 (-0.9;0.4)	0 (-0.4;1)	0 (-0.9;1.4)	0 (-0.5;1)	0.7 (-0.3;1.4)	0 (-0.9;1)	0 (0;1.4)	0.5 (0;1.4)	-0.1 (-0.9;0.9)	1 (0.1;1.2)	0 (-0.9;1)	0.2 (-0.3;0.8)	0 (-0.4;1.2)	0.2 (-0.1;0.7)	0 (-0.9;1)	0 (-0.4;1.4)	0 (-0.4;1)	0 (-0.9;1.4)	0 (-0.1;1)	-0.2 (-1;1.3)	0.2 (0;1)	0 (-0.9;0.8)	0.7 (-0.2;1.3)
Δ Legumes	-0.3 (-0.6;-0.1)	-0.1 (-0.5;-0.1)	-0.2 (-0.4;-0.1)	-0.3 (-0.6;-0.1)	-0.1 (-0.3;-0.1)	-0.6 (-0.7;-0.4)	-0.2 (-0.5;-0.1)	-0.4 (-0.6;-0.2)	-0.1 (-0.4;-0.1)	-0.4 (-0.6;-0.3)	-0.2 (-0.5;-0.1)	-0.3 (-0.6;-0.1)	-0.3 (-0.6;-0.1)	-0.3 (-0.4;-0.1)	-0.4 (-0.5;-0.1)	-0.2 (-0.5;-0.1)	-0.1 (-0.6;-0.1)	-0.3 (-0.4;-0.1)	-0.5 (-0.6;-0.3)	-0.1 (-0.4;-0.1)	-0.2 (-0.4;-0.1)	-0.3 (-0.5;-0.1)	-0.3 (-0.5;-0.2)	-0.1 (-0.6;-0.1)	-0.3 (-0.6;-0.1)	-0.1 (-0.5;-0.1)	-0.3 (-0.5;-0.1)	-0.2 (-0.5;-0.1)	-0.3 (-0.6;-0.1)	-0.2 (-0.5;-0.1)
Δ Eggs	0.1 (0;0.1)	0.1 (-0.1;0.2)	0 (-0.1;0.1)	0.1 (0;0.2)	0.1 (0;0.2)	0.1 (0;0.1)	0.1 (0;0.2)	0.1 (0.1;0.2)	0.1 (0;0.2)	0 (0;0.1)	0.1 (0;0.3)	0 (0;0.1)	0.1 (0;0.1)	0 (0;0.2)	0.1 (0;0.3)	0.1 (0;0.1)	0.1 (0;0.1)	0.1 (0;0.2)	0.1 (0.1;0.6)	0 (0;0.2)	0 (-0.2;0.1)	0.1 (0;0.2)	0 (0;0.1)	0.1 (0;0.1)	0 (0;0.1)	0.1 (0.1;0.2)	0 (0;0.1)	0.1 (0;0.3)	0 (-0.1;0.1)	0.1 (0;0.2)
Δ Sweets	-0.4 (-0.5;-0.1)	-0.4 (-0.8;-0.1)	-0.4 (-0.5;-0.3)	-0.4 (-0.8;-0.1)	-0.4 (-0.6;-0.1)	-0.4 (-0.7;-0.1)	-0.4 (-0.6;-0.1)	-0.5 (-0.7;-0.4)	-0.4 (-0.6;-0.1)	-0.4 (-0.6;-0.1)	-0.4 (-0.7;-0.1)	-0.4 (-0.6;-0.3)	-0.5 (-0.9;-0.1)	-0.3 (-0.4;-0.1)	-0.4 (-0.6;-0.1)	-0.4 (-0.6;-0.2)	-0.4 (-0.7;0)	-0.4 (-0.6;-0.1)	-0.4 (-0.8;-0.2)	-0.4 (-0.6;-0.1)	-0.4 (-0.5;-0.4)	-0.3 (-0.6;-0.1)	-0.4 (-0.5;-0.2)	-0.4 (-0.7;-0.1)	-0.4 (-0.6;-0.1)	-0.3 (-0.6;-0.1)	-0.5 (-0.8;-0.2)	-0.3 (-0.5;-0.1)	-0.6 (-0.8;-0.4)	-0.2 (-0.4;-0.1)
Δ Soda	0 (-0.1;0)	-0.1 (-0.1;0)	0 (-0.3;0)	0 (-0.1;0)	-0.1 (-0.1;0)	0 (-0.1;0)	-0.1 (-0.1;0)	0 (0;0)	-0.1 (-0.1;0)	0 (-0.1;0)	0 (-0.1;0)	-0.1 (-0.2;0)	0 (-0.1;0)	-0.1 (-0.1;-0.1)	0 (-0.1;0)	-0.1 (-0.3;0)	0 (-0.1;0)	-0.1 (-0.1;0)	-0.1 (-0.2;0)	-0.1 (-0.1;0)	-0.4 (-0.4;-0.2)	0 (-0.1;0)	0 (-0.1;0)	-0.1 (-0.1;0)	-0.1 (-0.1;0)	0 (-0.1;0)	-0.1 (-0.1;0)	0 (-0.1;0)	0 (-0.1;0)	0 (-0.1;0)
Δ Alcoholic beverages	-0.1 (-1;0)	0 (-0.2;0)	-0.2 (-0.6;0)	0 (-0.4;0)	-0.1 (-0.7;0)	0 (-0.1;0)	0 (-0.5;0)	-0.1 (-0.6;0.1)	-0.1 (-0.6;0)	0 (-0.1;0)	0 (-0.3;0)	-0.1 (-0.8;0)	0 (-0.1;0)	-0.3 (-1;-0.1)	0 (-0.8;0)	-0.1 (-0.4;0)	0 (-0.1;0)	-0.1 (-1;0)	0 (-0.5;0)	-0.1 (-0.6;0)	-0.1 (-0.4;0)	-0.1 (-0.7;0)	0 (-0.1;0)	-0.1 (-0.7;0)	-0.1 (-1;0)	0 (-0.2;0)	-0.1 (-0.8;0)	0 (-0.3;0)	0 (-0.1;0)	-0.2 (-1;0)
Δ PREDIMED score	1 (-1;2)	0 (-2.5;2.5)	-0.5 (-2.5;1.5)	1 (-1.8;2)	2 (-1;2)	0 (-3;1)	1 (-1.3;2.3)	-1.5 (-3;0.5)	2 (-2.5;2.5)	0 (-2;1)	0.5 (-2.3;2)	0 (-1.5;2.3)	0 (-2.5;2)	1 (-1;2)	1 (-2.8;2)	0 (-1;2)	0 (-3.5;1.5)	1 (-1;2)	2.5 (0.8;3)	0 (-2.5;2)	1 (-2;3)	0 (-2.5;2)	-1 (-2.5;1.5)	1 (-1;2)	-1 (-3;1)	2 (-0.5;3)	0 (-2.8;1.8)	1 (-0.8;2.8)	-1.5 (-3.8;-0.3)	2 (1.3;2.8)

Gender “1” refers to male, “0” refers to female; for the other variables “1” refers to “yes”, “0” refers to “no”.
Δ refers to difference between week 0 and week 9.
Data are reported as median and interquartile range.
Significant associations are indicated by green cells. Significance refers to the Kruskal-Wallis test.
° Weekly, according to the Food Frequency Questionnaire.
W0, week 0; bDMARDs, biological disease-modifying antirheumatic drugs; IL, interleukin; MDA, Minimal Disease Activity; PASS, Patient Acceptable Symptom State; PREDIMED, PREvención con DIeta MEDiterránea.
