# Supplementary material for: Ketogenic diet improves disease activity and cardiovascular risk in psoriatic arthritis: A proof of concept study
Source: PLoS One. 2025 Apr 22;20(4):e0321140. doi: 10.1371/journal.pone.0321140 (PMC12013891; doi:10.1371/journal.pone.0321140)
Supplement: S24 Table — (PDF) [file pone.0321140.s024.pdf]

Table S24. Analysis of the association between categorical variables at W0 and the modification of cardiovascular parameters during the study.

	Gender		Smoke ever		Higher education		Employed		bDMARDs		Axial involvement		Comorbidities		Metabolic syndrome	
	1	0	1	0	1	0	1	0	1	0	1	0	1	0	1	0
CUORE§	-0.9 (-2.1;-0.3)	0 (-0.7;0.2)	-1.2 (-3.8;-0.1)	-0.3 (-0.9;0.3)	0 (-0.9;0.3)	-0.7 (-2.2;-0.6)	-0.4 (-1.3;0)	-0.2 (-8.5;0.7)	-0.9 (-1.9;-0.1)	0.1 (-0.7;0.4)	-0.3 (-1;0.3)	-0.7 (-1.4;0)	-0.7 (-1.9;0.3)	-0.2 (-0.7;0)	-0.6 (-1.9;0.2)	-0.2 (-1.1;0.1)
SCORE ¤^	-0.8 (-2.6;-0.3)	-0.1 (-0.5;0.2)	-0.7 (-2.5;-0.2)	-0.3 (-0.8;0.4)	-0.1 (-0.8;0.2)	-1.1 (-1.2;-0.3)	-0.3 (-1.1;0)	0.1 (-2;1.1)	-0.3 (-1.2;-0.1)	-0.3 (-0.8;0.5)	-0.3 (-0.8;0.2)	-0.5 (-1.4;-0.1)	-0.8 (-1.2;0.1)	-0.1 (-0.3;0.2)	-0.5 (-1.3;0)	-0.2 (-0.6;0.1)
SBP	0 (-10;5)	0 (-5;0)	-15 (-27.5;-2.5)	0 (0;3.8)	0 (-5;0)	0 (-7.5;0)	0 (-6.3;1.3)	0 (-7.5;0)	0 (-15;0)	0 (0;10)	0 (-1.3;0)	-2.5 (-15;6.3)	0 (-7.5;0)	0 (-5;0)	0 (-3.8;0)	0 (-8.8;0)
DBP	0 (0;5)	0 (0;2.5)	0 (-7.5;0)	0 (0;5)	0 (0;0)	0 (-5;5)	0 (0;5)	0 (-3.8;1.3)	0 (0;0)	0 (0;5)	0 (0;1.3)	0 (-2.5;5)	0 (-5;5)	0 (0;0)	0 (0;3.8)	0 (0;3.8)

	Cardiovascular comorbidities		W0 elevated IL-1β		W0 elevated IL-6		W0 elevated fecal calprotectin		W0 MDA		W0 PASS		W0 physical activity°	
	1	0	1	0	1	0	1	0	1	0	1	0	1	0
CUORE§	-1.7 (-2.2;-1)	0 (-0.4;0.3)	-1.4 (-2.7;-0.5)	-0.3 (-1.1;0.1)	0 (-0.5;0)	-0.4 (-1.5;0.3)	0.1 (-0.7;0.6)	-0.4 (-1.7;0)	-0.4 (-0.9;0.4)	-0.3 (-1.9;0.1)	-0.2 (-0.9;0.4)	-0.5 (-2;-0.1)	-0.1 (-1.1;0.2)	-0.6 (-1.5;0)
SCORE ¤^	-1.1 (-2;-0.7)	0 (-0.3;0.5)	-0.7 (-1.5;-0.1)	-0.3 (-0.9;0.1)	-0.1 (-0.4;0)	-0.3 (-1.1;0.3)	-0.3 (-0.9;0.9)	-0.3 (-1.1;0)	-0.8 (-1.1;0)	-0.3 (-0.7;0.2)	-0.5 (-1;0.3)	-0.3 (-0.9;0.1)	-0.4 (-1.3;0.1)	-0.3 (-0.6;0)
SBP	-5 (-20;0)	0 (0;0)	-5 (-12.5;0)	0 (-5;2.5)	-7.5 (-15;-3.8)	0 (-2.5;2.5)	0 (-2.5;7.5)	0 (-10;0)	0 (0;10)	-5 (-15;0)	0 (0;8.8)	-5 (-17.5;0)	0 (-3.8;3.8)	0 (-8.8;0)
DBP	0 (-10;2.5)	0 (0;5)	0 (-2.5;0)	0 (0;5)	0 (-2.5;0)	0 (0;5)	0 (0;2.5)	0 (0;5)	0 (0;5)	0 (0;0)	0 (0;5)	0 (0;0)	2.5 (0;5)	0 (0;0)

Gender “1” refers to male, “0” refers to female; for the other variables “1” refers to “yes”, “0” refers to “no”.

Δ refers to difference between week 0 and week 9.

Data are reported as median and interquartile range.

Significant associations are indicated by green cells. Significance refers to the Kruskal-Wallis test.

° Weekly, according to the Food Frequency Questionnaire.

¤ Computed from 19 subjects.

§ 10 year risk of cardiovascular events according to the Progetto CUORE estimator. SCORE2-OP (Older People) estimator was used for subjects >70 years. Values were adjusted for subjects with inflammatory arthritis. Probability is expressed as percentage of risk.

^ 10 year risk of cardiovascular events according to the ESC (European Society of Cardiology), SCORE2 (Systematic Coronary Risk Evaluation 2) estimator. Values were adjusted for subjects with inflammatory arthritis. Probability is expressed as percentage of risk.

W0, week 0; bDMARDs, biological disease-modifying antirheumatic drugs; IL, interleukin; MDA, Minimal Disease Activity; PASS, Patient Acceptable Symptom State; CUORE, cardiovascular unique offer reengineered; SCORE2, systematic coronary risk evaluation; SBP, systolic blood pressure; DBP, diastolic blood pressure
