# Supplementary material for: Ketogenic diet improves disease activity and cardiovascular risk in psoriatic arthritis: A proof of concept study
Source: PLoS One. 2025 Apr 22;20(4):e0321140. doi: 10.1371/journal.pone.0321140 (PMC12013891; doi:10.1371/journal.pone.0321140)
Supplement: S25 Table — (PDF) [file pone.0321140.s025.pdf]

Table S25a. Association between continuous variables at W0 and the modification of continuous variables during the study.

	W0 weight		W0 BMI		W0 Abdominal circumference		W0 hsCRP		W0 ESR		W0 TNF-α		W0 Insulinemia		W0 PREDIMED		W0 SCORE2*§		W0 CUORE*^		W0 SBP		W0 DBP	
	Spearman's r_s	p*	Spearman's r_s	p*	Spearman's r_s	p*	Spearman's r_s	p*	Spearman's r_s	p*	Spearman's r_s	p*	Spearman's r_s	p*	Spearman's r_s	p*	Spearman's r_s	p*	Spearman's r_s	p*	Spearman's r_s	p*	Spearman's r_s	p*
Anthropometric measurements																								
Δ Weight	-0.445	0.049	-0.248	0.292	-0.235	0.319	-0.236	0.317	-0.086	0.719	0.458	0.042	-0.261	0.266	0.076	0.749	0.182	0.456	-0.078	0.751	-0.083	0.729	-0.086	0.718
Δ BMI	-0.289	0.217	-0.268	0.254	-0.213	0.367	-0.229	0.332	-0.096	0.688	0.469	0.037	-0.243	0.302	0.012	0.959	0.271	0.261	-0.103	0.676	-0.085	0.722	-0.086	0.718
Δ Abdominal circumference	-0.358	0.121	-0.095	0.690	-0.154	0.516	-0.156	0.513	-0.207	0.381	0.244	0.299	-0.202	0.392	0.075	0.752	0.348	0.145	0.297	0.217	0.218	0.357	0.358	0.122
Clinical measures																								
Δ TJC (0-68)	-0.625	0.003	-0.569	0.009	-0.451	0.046	-0.593	0.006	-0.314	0.177	0.208	0.380	-0.318	0.171	-0.020	0.933	-0.240	0.322	0.311	0.195	0.181	0.444	0.210	0.375
Δ SJC (0-68)	-0.159	0.503	-0.037	0.876	-0.043	0.856	-0.596	0.006	-0.314	0.177	0.237	0.314	-0.244	0.300	0.193	0.415	-0.230	0.343	0.087	0.723	-0.279	0.233	-0.203	0.392
Δ TJC (0-28)	-0.498	0.026	-0.559	0.010	-0.493	0.027	-0.207	0.380	-0.403	0.078	0.182	0.442	-0.508	0.022	0.081	0.735	0.088	0.719	0.037	0.879	-0.056	0.814	0.061	0.799
Δ SJC (0-28)	-0.217	0.358	-0.272	0.246	-0.126	0.596	-0.533	0.016	-0.504	0.023	0.162	0.494	-0.215	0.362	0.241	0.306	-0.005	0.984	0.140	0.567	-0.244	0.301	-0.083	0.727
Δ DAPSA	-0.170	0.474	-0.433	0.056	-0.228	0.333	-0.245	0.299	-0.291	0.214	0.358	0.121	-0.129	0.589	-0.150	0.527	0.126	0.609	0.032	0.898	0.189	0.425	0.255	0.277
Δ HAQ	-0.117	0.624	-0.382	0.097	-0.152	0.524	-0.147	0.537	-0.191	0.420	0.345	0.136	0.012	0.960	-0.215	0.362	0.041	0.868	-0.080	0.745	-0.025	0.915	0.027	0.911
Δ DAS28-CRP	0.002	0.995	-0.111	0.640	0.013	0.957	-0.304	0.192	-0.288	0.219	0.330	0.155	0.015	0.950	-0.139	0.558	0.094	0.702	0.080	0.745	0.147	0.536	0.173	0.466
Δ CDAI	-0.183	0.440	-0.393	0.087	-0.206	0.384	-0.155	0.515	-0.330	0.155	0.314	0.178	-0.101	0.671	-0.185	0.435	0.118	0.630	-0.055	0.825	0.144	0.545	0.210	0.375
Δ SDAI	-0.203	0.391	-0.397	0.083	-0.214	0.365	-0.173	0.466	-0.338	0.145	0.314	0.178	-0.118	0.620	-0.172	0.467	0.125	0.611	-0.030	0.903	0.171	0.472	0.231	0.327
Δ BSA	-0.207	0.381	-0.469	0.037	-0.345	0.136	-0.168	0.479	-0.137	0.565	-0.325	0.163	-0.276	0.239	0.027	0.911	0.228	0.348	0.155	0.525	-0.053	0.825	0.026	0.913
Δ PASI	-0.068	0.775	-0.141	0.554	-0.095	0.692	-0.147	0.536	-0.035	0.885	-0.166	0.483	-0.153	0.520	-0.230	0.328	0.197	0.419	0.173	0.478	-0.094	0.692	-0.143	0.548
Δ LEI	0.452	0.046	0.098	0.680	0.228	0.333	0.111	0.640	-0.118	0.619	0.074	0.755	0.054	0.820	-0.443	0.051	-0.146	0.551	-0.267	0.269	-0.134	0.574	-0.101	0.670
Δ SPARCC	0.508	0.022	0.079	0.741	0.226	0.338	0.100	0.674	-0.123	0.605	-0.171	0.471	0.124	0.603	-0.397	0.083	0.010	0.967	-0.143	0.558	0.005	0.985	0.066	0.782
Δ VAS pain	0.005	0.985	-0.209	0.375	-0.049	0.837	-0.054	0.820	-0.251	0.285	0.253	0.282	0.073	0.759	-0.174	0.463	-0.123	0.615	-0.198	0.417	0.041	0.864	0.097	0.684
Δ PGA	-0.023	0.922	-0.194	0.414	-0.041	0.863	-0.020	0.934	-0.264	0.261	0.233	0.323	0.080	0.737	-0.217	0.358	-0.127	0.605	-0.217	0.373	0.007	0.977	0.069	0.773
Δ PtGA	-0.062	0.795	-0.249	0.289	-0.083	0.728	-0.069	0.774	-0.226	0.339	0.289	0.217	0.042	0.859	-0.105	0.659	-0.174	0.477	-0.258	0.287	-0.020	0.933	0.020	0.932
Δ WPAI lost work hours	0.617	0.025	0.154	0.615	0.550	0.051	-0.074	0.810	-0.114	0.710	0.050	0.870	0.510	0.075	-0.304	0.312	0.033	0.918	-0.206	0.520	-0.449	0.124	-0.257	0.397
Δ WPAI impact	-0.162	0.533	-0.330	0.196	-0.136	0.603	-0.118	0.653	-0.293	0.253	0.306	0.232	-0.189	0.467	0.157	0.547	0.127	0.639	-0.260	0.332	-0.206	0.427	-0.109	0.678
Δ BASDAI	-0.043	0.857	-0.162	0.495	-0.170	0.474	-0.446	0.049	-0.303	0.194	0.201	0.397	0.037	0.876	-0.024	0.920	-0.219	0.368	0.217	0.371	0.093	0.697	0.065	0.784
Δ ASDAS-CRP	-0.011	0.965	-0.249	0.290	-0.116	0.628	-0.465	0.039	-0.280	0.232	0.351	0.130	0.102	0.669	-0.024	0.919	-0.247	0.307	-0.006	0.980	-0.073	0.761	-0.094	0.692
Inflammatory biomarkers																								
Δ hsCRP	0.199	0.400	-0.014	0.955	0.265	0.260	-0.298	0.203	-0.150	0.527	-0.001	0.997	0.081	0.733	-0.313	0.179	-0.197	0.419	-0.040	0.871	-0.225	0.341	-0.240	0.308
Δ ESR	-0.226	0.338	-0.218	0.355	-0.160	0.501	-0.235	0.318	0.186	0.432	0.144	0.545	-0.308	0.186	0.174	0.463	-0.184	0.450	-0.062	0.800	-0.186	0.432	-0.069	0.771
Δ TNFα	-0.205	0.399	-0.205	0.399	-0.317	0.186	0.143	0.560	0.239	0.325	-0.465	0.045	0.264	0.274	0.066	0.789	0.091	0.710	-0.006	0.980	0.315	0.190	0.183	0.454
Laboratory variables																								
Δ Total cholesterol	-0.083	0.729	-0.347	0.134	-0.116	0.625	-0.156	0.512	-0.143	0.547	-0.479	0.033	-0.011	0.964	-0.185	0.435	0.072	0.771	0.109	0.656	0.051	0.831	0.098	0.681
Δ HDL cholesterol	-0.315	0.177	-0.150	0.529	-0.054	0.823	-0.380	0.098	-0.062	0.796	0.008	0.975	-0.055	0.818	0.504	0.024	-0.096	0.695	0.250	0.302	0.094	0.693	0.188	0.427

Δ LDL cholesterol	0.092	0.701	-0.134	0.574	0.004	0.987	-0.072	0.761	-0.127	0.593	-0.566	0.009	0.096	0.689	-0.286	0.221	0.047	0.850	0.102	0.678	0.147	0.536	0.120	0.613
Δ Triglyceride	0.333	0.151	0.062	0.796	0.072	0.764	0.528	0.017	0.449	0.047	-0.118	0.620	0.079	0.740	-0.064	0.789	-0.004	0.989	-0.570	0.011	-0.289	0.216	-0.387	0.092
Δ AST	-0.032	0.892	-0.023	0.925	-0.118	0.621	0.202	0.392	-0.088	0.714	-0.150	0.529	-0.029	0.905	0.113	0.636	0.394	0.095	-0.099	0.685	-0.277	0.237	-0.288	0.218
Δ ALT	0.267	0.256	0.370	0.108	0.199	0.401	0.224	0.343	0.026	0.912	0.107	0.654	0.183	0.441	-0.125	0.599	0.070	0.776	-0.197	0.419	-0.318	0.171	-0.403	0.078
Δ GGT	-0.016	0.947	0.152	0.522	-0.068	0.775	0.253	0.282	0.422	0.064	0.208	0.378	-0.231	0.327	0.289	0.217	0.355	0.136	-0.091	0.711	-0.118	0.620	-0.359	0.120
Δ TSH	-0.258	0.272	-0.157	0.508	-0.175	0.461	0.365	0.113	0.168	0.478	-0.123	0.606	-0.238	0.313	0.287	0.220	0.509	0.026	-0.014	0.956	-0.065	0.786	-0.147	0.537
Δ Creatinine	-0.193	0.415	-0.123	0.604	0.035	0.883	-0.250	0.287	-0.169	0.477	0.168	0.480	0.143	0.547	-0.118	0.620	-0.004	0.989	-0.047	0.850	-0.003	0.991	-0.204	0.389
Δ Uricemia	0.251	0.286	0.053	0.823	0.081	0.733	0.136	0.567	-0.072	0.763	-0.322	0.167	0.177	0.454	-0.495	0.027	0.223	0.359	0.026	0.915	0.099	0.678	-0.003	0.990
Δ Blood glucose	-0.358	0.122	-0.454	0.044	-0.433	0.056	-0.014	0.955	0.153	0.519	0.345	0.137	-0.373	0.105	0.328	0.157	0.167	0.496	-0.284	0.239	-0.323	0.165	-0.242	0.305
Δ Insulinemia	-0.253	0.297	-0.249	0.304	-0.316	0.188	-0.177	0.468	0.108	0.660	0.444	0.057	-0.735	0.000	0.322	0.179	-0.053	0.830	-0.227	0.349	-0.262	0.278	-0.190	0.436
Δ Azotemia	-0.151	0.526	-0.014	0.955	0.034	0.886	-0.223	0.346	-0.276	0.239	0.411	0.072	-0.282	0.228	0.147	0.536	0.293	0.224	0.067	0.786	-0.206	0.384	-0.242	0.304
Δ Total proteins	-0.299	0.200	-0.307	0.188	-0.211	0.372	-0.172	0.469	-0.137	0.564	0.538	0.014	-0.094	0.693	-0.156	0.512	-0.302	0.209	-0.377	0.112	-0.209	0.376	-0.212	0.370
Δ Albumin	-0.099	0.677	-0.102	0.670	-0.094	0.695	0.275	0.241	0.242	0.305	0.071	0.766	0.568	0.009	0.010	0.967	-0.190	0.436	-0.109	0.657	0.275	0.240	0.269	0.251
Δ α1-globulin	-0.066	0.783	0.020	0.932	0.023	0.924	-0.277	0.237	-0.108	0.649	0.072	0.761	-0.451	0.046	0.318	0.172	-0.016	0.947	0.157	0.521	-0.015	0.949	0.028	0.906
Δ α2-globulin	0.026	0.912	-0.002	0.995	0.109	0.649	-0.446	0.049	-0.091	0.702	0.005	0.982	-0.473	0.035	0.085	0.722	0.142	0.561	0.329	0.169	-0.317	0.174	-0.239	0.311
Δ β1-globulin	0.471	0.036	0.555	0.011	0.604	0.005	0.185	0.434	-0.146	0.540	0.002	0.995	0.301	0.197	0.041	0.864	0.280	0.246	0.116	0.636	0.190	0.423	0.219	0.353
Δ β2-globulin	0.327	0.159	0.295	0.207	0.277	0.237	-0.052	0.829	-0.281	0.230	-0.235	0.319	-0.156	0.511	-0.263	0.262	-0.084	0.733	-0.323	0.177	-0.593	0.006	-0.672	0.001
Δ γ-globulin	0.056	0.815	-0.099	0.678	-0.166	0.484	0.194	0.412	-0.114	0.634	-0.362	0.117	-0.247	0.293	-0.168	0.478	0.173	0.478	-0.279	0.247	-0.130	0.584	-0.232	0.326
Δ WBC	-0.374	0.104	-0.502	0.024	-0.450	0.046	-0.048	0.840	0.051	0.830	-0.028	0.907	-0.426	0.061	0.163	0.492	0.296	0.219	-0.105	0.668	-0.042	0.861	-0.021	0.929
Δ RBC	-0.327	0.159	-0.212	0.369	-0.294	0.208	0.049	0.838	0.339	0.143	0.366	0.113	-0.276	0.239	0.288	0.219	-0.199	0.415	-0.451	0.052	-0.316	0.175	-0.281	0.229
Δ Hb	-0.302	0.195	-0.342	0.139	-0.334	0.151	-0.057	0.812	0.261	0.267	0.393	0.086	-0.374	0.104	0.211	0.372	-0.141	0.564	-0.512	0.025	-0.412	0.071	-0.372	0.106
Δ MCV	-0.136	0.567	-0.139	0.558	-0.124	0.601	-0.153	0.520	-0.032	0.892	-0.068	0.777	-0.174	0.464	0.145	0.541	-0.148	0.547	-0.121	0.621	0.051	0.831	-0.059	0.805
Δ MCH	-0.068	0.774	-0.302	0.195	-0.156	0.510	-0.161	0.497	-0.326	0.160	0.003	0.990	-0.167	0.480	-0.171	0.471	0.003	0.990	0.016	0.947	0.118	0.620	0.149	0.531
Δ MCHC	0.037	0.877	-0.290	0.214	-0.045	0.851	-0.129	0.587	-0.232	0.324	0.002	0.992	-0.024	0.921	-0.217	0.358	0.209	0.391	-0.050	0.840	-0.119	0.617	-0.034	0.888
Δ RDW	-0.016	0.947	-0.497	0.026	-0.165	0.486	-0.318	0.172	-0.277	0.238	-0.236	0.317	-0.082	0.731	0.156	0.513	0.173	0.480	0.223	0.358	0.120	0.615	0.145	0.541
Δ Platelet	0.132	0.580	-0.052	0.828	0.005	0.985	-0.164	0.489	-0.288	0.218	-0.282	0.228	-0.161	0.496	-0.211	0.372	0.598	0.007	0.598	0.007	0.281	0.230	0.409	0.073
Δ Neutrophils	-0.272	0.246	-0.260	0.268	-0.292	0.211	-0.097	0.685	0.032	0.895	0.043	0.858	-0.375	0.103	0.083	0.727	0.272	0.260	-0.098	0.689	-0.055	0.819	-0.114	0.634
Δ Lymphocytes	-0.379	0.099	-0.524	0.018	-0.459	0.042	0.217	0.359	0.270	0.250	-0.173	0.466	-0.090	0.705	0.040	0.867	0.271	0.263	-0.017	0.946	0.278	0.236	0.234	0.320
Δ Monocytes	-0.096	0.689	-0.211	0.373	-0.096	0.688	0.003	0.990	0.139	0.558	0.010	0.967	-0.340	0.142	0.147	0.536	0.313	0.193	-0.171	0.483	-0.128	0.592	-0.111	0.640
Δ Eosinophilic	0.075	0.755	-0.047	0.845	-0.133	0.575	0.283	0.226	0.060	0.803	-0.169	0.477	0.047	0.844	-0.133	0.577	-0.329	0.169	-0.224	0.357	0.242	0.303	0.325	0.162
Δ Basophil	-0.294	0.208	-0.371	0.107	-0.422	0.064	-0.050	0.835	-0.122	0.609	-0.227	0.335	-0.164	0.491	-0.062	0.795	-0.036	0.883	-0.235	0.333	-0.229	0.331	-0.349	0.132
Urine test																								
Δ pH	-0.102	0.678	-0.387	0.102	-0.305	0.204	0.051	0.835	-0.053	0.830	0.281	0.243	0.164	0.503	-0.025	0.921	-0.443	0.066	-0.296	0.233	0.036	0.882	0.256	0.289
Δ Protein	0.095	0.698	0.355	0.135	0.475	0.040	0.142	0.561	0.056	0.820	0.385	0.103	0.346	0.147	0.344	0.150	0.023	0.927	0.117	0.644	0.138	0.574	0.260	0.281
Δ Hb	-0.209	0.392	-0.528	0.020	-0.332	0.165	-0.259	0.285	-0.216	0.375	0.018	0.941	-0.473	0.041	0.052	0.833	0.146	0.563	0.001	0.996	-0.280	0.245	-0.282	0.243
Δ Ketones	0.261	0.280	0.132	0.589	0.243	0.315	-0.251	0.300	-0.104	0.672	-0.005	0.985	0.115	0.639	-0.140	0.568	-0.149	0.555	0.152	0.548	0.174	0.475	0.082	0.738
Δ Urobilinogen	-0.344	0.149	-0.344	0.149	-0.345	0.148	-0.324	0.176	-0.345	0.148	0.258	0.286	-0.344	0.149	0.110	0.655	-0.328	0.185	-0.351	0.154	-0.304	0.206	-0.197	0.420
Δ Specific weight	0.338	0.158	0.340	0.154	0.470	0.042	-0.030	0.904	0.090	0.713	0.569	0.011	-0.106	0.665	0.393	0.096	-0.115	0.648	-0.232	0.355	-0.369	0.120	-0.339	0.156
Intestinal permeability test																								
Δ Diuresis 6h	-0.336	0.148	-0.484	0.030	-0.400	0.081	-0.168	0.478	-0.002	0.992	0.332	0.153	-0.091	0.702	-0.027	0.910	0.092	0.709	0.224	0.357	0.292	0.212	0.381	0.097
Δ Lactulose	-0.120	0.613	-0.230	0.329	-0.079	0.740	-0.158	0.506	-0.139	0.560	0.085	0.722	-0.121	0.611	0.157	0.508	0.203	0.405	0.248	0.307	-0.062	0.794	0.170	0.474

Δ Mannitol	-0.298	0.202	-0.138	0.561	-0.325	0.163	0.052	0.827	0.084	0.724	0.532	0.016	-0.367	0.111	-0.026	0.913	0.004	0.989	-0.146	0.552	-0.269	0.251	-0.197	0.406
PREDIMED	0.485	0.030	0.540	0.014	0.511	0.021	0.590	0.006	0.187	0.430	0.017	0.944	0.513	0.021	-0.483	0.031	-0.05	0.838	-0.297	0.217	-0.039	0.871	-0.01	0.968
Cardiovascular parameters																								
SCORE2¤^	-0.061	0.805	-0.301	0.210	-0.067	0.785	0.004	0.987	-0.091	0.712	0.155	0.527	0.039	0.875	-0.303	0.207	-0.339	0.156	-0.561	0.013	-0.442	0.058	-0.457	0.049
CUORE§	-0.263	0.276	-0.517	0.023	-0.238	0.327	-0.061	0.804	0.043	0.862	0.284	0.238	-0.054	0.828	-0.126	0.606	-0.242	0.318	-0.604	0.006	-0.434	0.064	-0.472	0.042
SBP	-0.281	0.230	-0.553	0.011	-0.258	0.271	-0.235	0.318	-0.117	0.623	0.228	0.333	-0.175	0.460	0.180	0.448	-0.047	0.847	-0.331	0.166	-0.412	0.071	-0.290	0.214
DBP	-0.417	0.067	-0.480	0.032	-0.260	0.268	-0.259	0.270	-0.137	0.565	-0.006	0.979	-0.237	0.314	0.159	0.503	-0.187	0.443	-0.295	0.220	-0.300	0.198	-0.213	0.368

Δ refers to difference between week 0 and week 9.

* Significance refers to the Spearman correlation test, indicated by the coefficient $r_{s.}$

¤ Computed from 19 subjects.

§ 10 year risk of cardiovascular events according to the Progetto estimator. SCORE2-OP (Older People) estimator was used for subjects >70 years. Values were adjusted for subjects with inflammatory arthritis. Probability is expressed as percentage of risk.

^ 10 year risk of cardiovascular events according to the ESC (European Society of Cardiology), SCORE2 (Systematic Coronary Risk Evaluation 2) estimator. Values were adjusted for subjects with inflammatory arthritis. Probability is expressed as percentage of risk.

The subsequent baseline variables were excluded from the analysis of the study group due to inadequate case number: elevated IL-1α, fibromyalgia, uveitis, inflammatory bowel disease, HLA-B27.

W0, week 0; BMI, Body Mass Index; hsCRP, High Sensitivity C Reactive Protein; ESR, Erythrocyte Sedimentation Rate; TNFα, Tumor Necrosis Factor alpha; CUORE, cardiovascular unique offer reengineered; SCORE2, systematic coronary risk evaluation; SBP, systolic blood pressure; DBP, diastolic blood pressure; PREDIMED, PREvención con DIeta MEDiterránea; TJC, tender joint count; SJC, swollen joint count; DAPSA, disease activity index in psoriatic arthritis; HAQ, Health Assessment Questionnaire; DAS28-CRP, disease activity score on 28 joints with C reactive protein; CDAI, clinical disease activity index; SDAI, Simple Disease Activity Index; BSA, Body Surface Area; PASI, Psoriasis Area Severity Index; LEI, Leeds Enthesitis Index; SPARCC, Spondylarthritis Research Consortium of Canada; VAS, Visual Analogue Scale; PtGA, patient global assessment; PGA, Physician Global Assessment; WPAI, Work Productivity and activity Impairment questionnaire; BASDAI, Bath Ankylosing Spondylitis Disease Activity Index; ASDAS-CRP, Ankylosing Spondylitis Disease Activity Score – C Reactive Protein; HDL, High Density Lipoprotein; LDL, Low Density Lipoprotein; ALT, alanine aminotransferase; AST, aspartate aminotransferase; GGT, gamma glutamyl transpeptidase; TSH, thyroid-stimulating hormone; WBC, white blood cells; RBC, red blood cells; Hb; hemoglobin; MCV, mean corpuscular volume; MCH, mean corpuscular hemoglobin; MCHC, mean corpuscular hemoglobin concentration; RDW, red cell distribution width.

Table S25b. Association between continuous variables at W0 and the modification of continuous variables during the study.

	W0 Age		DD Arthritis		DD Psoriasis		W0 DAPSA		W0 DAS28-CRP		W0 PASI		W0 BSA		W0 BASDAI		W0 ASDAS-CRP		W0 LEI		W0 SPARCC		W0 VAS pain		W0 PGA		W0 PtGA		W0 WPAI lost work hours		W0 WPAI impact	
	Spearman's r_s	p*	Spearman's r_s	p*	Spearman's r_s	p*	Spearman's r_s	p*	Spearman's r_s	p*	Spearman's r_s	p*	Spearman's r_s	p*	Spearman's r_s	p*	Spearman's r_s	p*	Spearman's r_s	p*	Spearman's r_s	p*	Spearman's r_s	p*	Spearman's r_s	p*	Spearman's r_s	p*	Spearman's r_s	p*	Spearman's r_s	p*
Anthropometric measurements																																
Δ Weight	0.190	0.423	0.090	0.705	-0.220	0.350	-0.577	0.008	-0.529	0.016	-0.356	0.124	-0.369	0.109	-0.355	0.124	-0.282	0.228	0.019	0.937	0.243	0.302	-0.314	0.177	-0.354	0.126	-0.376	0.103	-0.367	0.162	-0.551	0.018
Δ BMI	0.139	0.560	0.081	0.734	-0.195	0.410	-0.669	0.001	-0.623	0.003	-0.330	0.156	-0.380	0.098	-0.435	0.055	-0.352	0.128	-0.132	0.579	0.018	0.939	-0.398	0.082	-0.424	0.063	-0.455	0.044	-0.436	0.092	-0.598	0.009
Δ Abdominal circumference	0.449	0.047	0.162	0.495	-0.176	0.458	-0.457	0.043	-0.450	0.046	-0.181	0.444	-0.113	0.637	-0.232	0.325	-0.165	0.486	0.281	0.229	0.261	0.266	-0.207	0.380	-0.248	0.291	-0.233	0.323	-0.162	0.549	-0.257	0.303
Clinical variables																																
Δ TJC (0-68)	0.205	0.386	0.614	0.004	-0.083	0.729	-0.334	0.150	-0.307	0.188	-0.311	0.181	-0.292	0.211	-0.067	0.779	-0.057	0.810	0.123	0.604	0.265	0.259	0.054	0.821	-0.028	0.908	-0.005	0.982	0.443	0.086	0.046	0.855
Δ SJC (0-68)	-0.215	0.362	0.005	0.982	-0.196	0.407	-0.143	0.547	-0.302	0.195	-0.284	0.225	-0.256	0.276	-0.112	0.639	-0.155	0.514	0.333	0.151	0.491	0.028	-0.110	0.645	-0.172	0.468	-0.134	0.574	-0.020	0.942	-0.286	0.249
Δ TJC (0-28)	0.104	0.662	0.466	0.038	-0.196	0.406	-0.388	0.091	-0.407	0.075	-0.380	0.099	-0.400	0.081	-0.152	0.522	-0.118	0.621	0.049	0.837	0.135	0.570	-0.111	0.641	-0.149	0.529	-0.144	0.545	0.241	0.368	-0.272	0.275
Δ SJC (0-28)	-0.211	0.371	0.080	0.739	-0.049	0.836	-0.296	0.205	-0.416	0.068	-0.336	0.147	-0.337	0.146	-0.262	0.265	-0.339	0.144	0.343	0.139	0.412	0.071	-0.283	0.226	-0.346	0.135	-0.315	0.176	-0.159	0.557	-0.570	0.014
Δ DAPSA	0.081	0.733	0.060	0.801	0.191	0.420	-0.871	0.000	-0.845	0.000	-0.217	0.359	-0.311	0.182	-0.860	0.000	-0.841	0.000	-0.462	0.040	-0.400	0.080	-0.790	0.000	-0.760	0.000	-0.800	0.000	-0.280	0.293	-0.666	0.003
Δ HAQ	0.058	0.808	0.085	0.721	0.064	0.790	-0.669	0.001	-0.619	0.004	-0.130	0.585	-0.244	0.300	-0.867	0.000	-0.846	0.000	-0.400	0.081	-0.272	0.247	-0.815	0.000	-0.818	0.000	-0.832	0.000	-0.300	0.258	-0.541	0.020
Δ DAS28-CRP	0.154	0.515	-0.262	0.265	0.313	0.179	-0.620	0.004	-0.758	0.000	-0.364	0.115	-0.383	0.096	-0.651	0.002	-0.678	0.001	-0.311	0.182	-0.187	0.430	-0.594	0.006	-0.535	0.015	-0.584	0.007	-0.168	0.533	-0.519	0.027
Δ CDAI	0.083	0.726	-0.042	0.860	0.171	0.471	-0.800	0.000	-0.803	0.000	-0.291	0.212	-0.371	0.107	-0.826	0.000	-0.803	0.000	-0.425	0.062	-0.338	0.145	-0.765	0.000	-0.719	0.000	-0.764	0.000	-0.221	0.411	-0.661	0.003
Δ SDAI	0.112	0.637	-0.039	0.870	0.191	0.420	-0.798	0.000	-0.803	0.000	-0.304	0.193	-0.374	0.104	-0.802	0.000	-0.779	0.000	-0.416	0.068	-0.332	0.153	-0.735	0.000	-0.687	0.001	-0.734	0.000	-0.164	0.544	-0.643	0.004
Δ BSA	-0.090	0.705	0.101	0.673	-0.081	0.734	-0.262	0.264	-0.253	0.282	-0.592	0.006	-0.682	0.001	-0.100	0.674	-0.155	0.514	0.093	0.696	-0.134	0.573	-0.002	0.992	-0.008	0.973	-0.098	0.682	-0.005	0.987	-0.372	0.129
Δ PASI	0.143	0.549	-0.058	0.810	0.020	0.934	-0.013	0.956	-0.162	0.496	-0.769	0.000	-0.731	0.000	0.225	0.341	0.168	0.478	-0.101	0.671	0.049	0.839	0.330	0.156	0.348	0.132	0.266	0.256	0.184	0.495	-0.076	0.765
Δ LEI	-0.337	0.146	-0.141	0.553	-0.084	0.726	-0.301	0.197	-0.343	0.139	0.165	0.488	0.050	0.835	-0.536	0.015	-0.527	0.017	-0.786	0.000	-0.590	0.006	-0.564	0.010	-0.471	0.036	-0.494	0.027	-0.314	0.237	-0.145	0.567
Δ SPARCC	-0.316	0.174	-0.204	0.388	-0.136	0.567	-0.321	0.168	-0.317	0.173	0.263	0.263	0.118	0.622	-0.556	0.011	-0.536	0.015	-0.630	0.003	-0.798	0.000	-0.571	0.009	-0.449	0.047	-0.501	0.024	-0.298	0.262	-0.099	0.696
Δ VAS pain	-0.122	0.610	-0.150	0.528	0.058	0.807	-0.618	0.004	-0.619	0.004	-0.090	0.707	-0.223	0.345	-0.906	0.000	-0.883	0.000	-0.381	0.097	-0.345	0.136	-0.876	0.000	-0.823	0.000	-0.850	0.000	-0.190	0.480	-0.526	0.025
Δ PGA	-0.072	0.764	-0.212	0.370	0.090	0.705	-0.557	0.011	-0.583	0.007	-0.133	0.575	-0.234	0.321	-0.863	0.000	-0.845	0.000	-0.362	0.117	-0.289	0.217	-0.834	0.000	-0.776	0.000	-0.804	0.000	-0.137	0.614	-0.484	0.042
Δ PtGA	-0.106	0.658	-0.157	0.508	0.062	0.794	-0.592	0.006	-0.602	0.005	-0.163	0.491	-0.305	0.191	-0.870	0.000	-0.854	0.000	-0.348	0.132	-0.296	0.205	-0.839	0.000	-0.778	0.000	-0.825	0.000	-0.132	0.625	-0.520	0.027
Δ WPAI lost work hours	-0.407	0.167	-0.148	0.630	-0.322	0.283	-0.215	0.481	-0.235	0.440	0.375	0.207	0.197	0.520	-0.505	0.078	-0.540	0.057	-0.207	0.497	-0.260	0.391	-0.665	0.013	-0.602	0.029	-0.631	0.021	-0.903	0.000	-0.331	0.270
Δ WPAI impact	-0.107	0.684	-0.166	0.524	-0.006	0.981	-0.635	0.006	-0.726	0.001	-0.310	0.225	-0.449	0.071	-0.742	0.001	-0.753	0.000	-0.298	0.245	-0.198	0.445	-0.723	0.001	-0.644	0.005	-0.724	0.001	-0.195	0.486	-0.750	0.001
Δ BASDAI	-0.210	0.373	0.175	0.461	-0.292	0.211	-0.380	0.099	-0.519	0.019	-0.089	0.709	-0.209	0.376	-0.591	0.006	-0.631	0.003	0.009	0.970	-0.057	0.811	-0.568	0.009	-0.542	0.014	-0.570	0.009	-0.169	0.532	-0.237	0.343
Δ ASDAS-CRP	-0.268	0.253	0.000	1.000	0.031	0.898	-0.433	0.057	-0.615	0.004	-0.161	0.499	-0.256	0.277	-0.643	0.002	-0.715	0.000	-0.182	0.443	-0.148	0.534	-0.641	0.002	-0.593	0.006	-0.639	0.002	-0.336	0.204	-0.473	0.048
Inflammatory biomarkers																																
Δ hsCRP	-0.015	0.951	-0.283	0.227	0.182	0.442	-0.023	0.925	-0.064	0.789	0.084	0.726	0.118	0.621	-0.185	0.436	-0.236	0.318	-0.449	0.047	-0.351	0.129	-0.177	0.457	-0.073	0.759	-0.151	0.524	-0.030	0.912	0.145	0.565
Δ ESR	0.073	0.759	-0.041	0.862	0.368	0.110	-0.059	0.806	0.032	0.895	0.023	0.924	0.112	0.638	0.043	0.857	-0.008	0.973	0.031	0.895	0.064	0.790	0.072	0.763	0.009	0.970	-0.016	0.947	0.015	0.956	0.109	0.666
Δ TNFα	-0.125	0.610	-0.032	0.898	-0.144	0.557	-0.025	0.920	0.047	0.847	-0.194	0.427	-0.349	0.142	-0.066	0.788	-0.059	0.809	0.388	0.100	-0.041	0.867	0.041	0.868	0.017	0.946	-0.009	0.971	-0.051	0.857	-0.242	0.349
Δ Insulinemia	0.041	0.867	0.128	0.601	0.042	0.864	-0.316	0.188	-0.235	0.333	-0.009	0.969	0.014	0.955	-0.085	0.728	-0.025	0.920	-0.234	0.335	-0.077	0.755	-0.060	0.806	-0.092	0.708	-0.110	0.655	0.110	0.697	-0.080	0.759
Laboratory variables																																

Δ Total cholesterol	-0.148	0.533	0.206	0.383	-0.170	0.473	-0.163	0.492	-0.010	0.967	-0.263	0.262	-0.386	0.092	-0.007	0.977	-0.040	0.868	0.007	0.977	-0.152	0.522	0.074	0.758	0.046	0.849	0.001	0.997	-0.153	0.571	-0.193	0.444
Δ HDL cholesterol	-0.086	0.717	0.188	0.427	-0.249	0.289	-0.260	0.268	-0.136	0.567	0.005	0.983	-0.088	0.713	-0.213	0.367	-0.234	0.320	0.513	0.021	0.246	0.296	-0.201	0.395	-0.231	0.328	-0.260	0.269	-0.112	0.679	-0.348	0.158
Δ LDL cholesterol	-0.076	0.750	0.068	0.777	-0.130	0.585	0.075	0.753	0.150	0.527	-0.303	0.194	-0.388	0.091	0.238	0.311	0.224	0.343	0.014	0.953	-0.190	0.424	0.326	0.160	0.332	0.153	0.286	0.221	0.142	0.599	0.105	0.678
Δ Triglyceride	-0.241	0.307	-0.241	0.306	0.119	0.619	0.198	0.403	0.263	0.263	0.208	0.378	0.099	0.676	0.045	0.850	0.074	0.758	-0.473	0.035	-0.501	0.024	0.014	0.953	0.075	0.752	0.041	0.864	-0.078	0.775	0.013	0.959
Δ AST	-0.184	0.438	-0.272	0.246	-0.154	0.516	-0.018	0.940	-0.147	0.536	-0.048	0.840	-0.069	0.773	-0.215	0.362	-0.264	0.261	0.124	0.602	0.106	0.658	-0.283	0.226	-0.243	0.302	-0.251	0.286	-0.389	0.137	-0.467	0.050
Δ ALT	-0.113	0.636	-0.309	0.184	-0.165	0.486	0.257	0.274	0.058	0.808	0.020	0.933	0.056	0.815	0.009	0.970	-0.029	0.903	-0.047	0.845	0.204	0.388	-0.095	0.689	-0.056	0.814	-0.057	0.810	-0.421	0.104	-0.014	0.957
Δ GGT	0.230	0.329	-0.257	0.274	0.201	0.396	0.089	0.710	-0.020	0.932	-0.286	0.221	-0.260	0.267	0.234	0.321	0.205	0.387	-0.237	0.315	0.018	0.941	0.242	0.304	0.316	0.174	0.228	0.334	0.082	0.762	-0.156	0.537
Δ TSH	0.313	0.179	-0.108	0.652	0.209	0.376	0.003	0.990	0.153	0.518	-0.109	0.647	-0.124	0.601	-0.025	0.917	-0.036	0.880	0.001	0.997	0.110	0.645	0.005	0.985	0.000	0.999	-0.022	0.927	0.220	0.413	-0.272	0.275
Δ Creatinine	0.288	0.219	0.030	0.900	-0.048	0.840	-0.083	0.728	-0.108	0.649	0.109	0.646	0.109	0.647	-0.076	0.750	-0.080	0.737	-0.238	0.313	-0.054	0.822	-0.085	0.722	0.025	0.918	-0.050	0.835	-0.076	0.781	0.094	0.710
Δ Uricemia	0.221	0.350	-0.121	0.611	0.238	0.313	0.275	0.240	0.178	0.454	0.002	0.994	0.081	0.733	0.359	0.120	0.329	0.156	-0.329	0.157	-0.292	0.212	0.370	0.108	0.437	0.054	0.411	0.072	0.045	0.868	0.424	0.080
Δ Blood glucose	-0.223	0.345	0.151	0.526	0.049	0.839	-0.446	0.049	-0.401	0.080	-0.386	0.093	-0.456	0.043	-0.245	0.298	-0.257	0.274	0.012	0.959	0.146	0.538	-0.242	0.304	-0.336	0.148	-0.345	0.136	-0.488	0.055	-0.775	0.000
Δ Azotemia	0.277	0.237	-0.169	0.475	0.331	0.155	-0.096	0.686	-0.279	0.234	-0.398	0.082	-0.231	0.328	0.170	0.475	0.120	0.613	-0.100	0.674	0.336	0.148	0.130	0.584	0.157	0.509	0.138	0.561	0.039	0.886	-0.292	0.240
Δ Total proteins	-0.082	0.732	-0.148	0.533	0.114	0.631	-0.428	0.060	-0.511	0.021	-0.354	0.126	-0.329	0.156	-0.499	0.025	-0.461	0.041	-0.291	0.214	-0.032	0.892	-0.467	0.038	-0.446	0.049	-0.481	0.032	-0.056	0.836	-0.353	0.151
Δ Albumin	-0.003	0.989	0.265	0.259	0.050	0.833	0.001	0.997	0.101	0.672	0.233	0.322	0.127	0.595	-0.271	0.248	-0.244	0.299	0.248	0.291	0.072	0.764	-0.275	0.240	-0.355	0.125	-0.283	0.226	-0.199	0.461	-0.017	0.946
Δ α1-globulin	0.096	0.687	-0.188	0.428	0.157	0.508	-0.143	0.549	-0.032	0.894	0.152	0.523	0.189	0.426	-0.132	0.579	-0.133	0.576	-0.006	0.981	-0.044	0.855	-0.104	0.662	-0.087	0.716	-0.112	0.638	0.353	0.180	0.014	0.956
Δ α2-globulin	0.145	0.543	-0.019	0.937	-0.049	0.838	-0.079	0.741	-0.120	0.613	-0.126	0.596	-0.061	0.799	0.047	0.843	-0.020	0.932	-0.125	0.599	0.049	0.839	0.079	0.742	0.091	0.702	0.043	0.856	0.110	0.685	0.055	0.830
Δ β1-globulin	0.057	0.813	-0.284	0.225	-0.101	0.672	0.072	0.764	0.055	0.818	0.076	0.749	0.103	0.666	0.212	0.370	0.275	0.241	0.024	0.920	-0.138	0.561	0.110	0.646	0.210	0.373	0.183	0.441	-0.119	0.661	-0.063	0.805
Δ β2-globulin	-0.190	0.423	-0.608	0.004	-0.205	0.385	0.348	0.133	0.060	0.803	-0.145	0.543	-0.087	0.714	0.161	0.498	0.095	0.691	-0.293	0.210	-0.063	0.793	0.097	0.685	0.266	0.258	0.176	0.458	0.048	0.861	0.213	0.396
Δ γ-globulin	-0.238	0.311	-0.303	0.194	0.087	0.714	0.122	0.607	0.025	0.917	-0.341	0.141	-0.330	0.156	0.308	0.187	0.284	0.225	-0.276	0.239	-0.233	0.323	0.324	0.163	0.406	0.076	0.346	0.135	0.169	0.533	-0.125	0.621
Δ WBC	0.237	0.315	0.129	0.587	-0.010	0.967	-0.469	0.037	-0.233	0.323	-0.022	0.926	-0.144	0.545	-0.413	0.070	-0.362	0.116	-0.037	0.876	-0.171	0.472	-0.320	0.170	-0.368	0.110	-0.395	0.085	-0.091	0.739	-0.329	0.183
Δ RBC	-0.034	0.888	-0.093	0.698	0.352	0.128	0.027	0.910	0.047	0.845	-0.308	0.186	-0.236	0.316	0.160	0.500	0.201	0.395	0.182	0.442	0.325	0.163	0.200	0.397	0.077	0.748	0.131	0.583	0.220	0.413	-0.224	0.371
Δ Hb	-0.051	0.831	-0.166	0.485	0.346	0.136	-0.151	0.526	-0.168	0.478	-0.446	0.049	-0.402	0.079	0.025	0.917	0.057	0.810	-0.029	0.905	0.079	0.741	0.078	0.745	0.013	0.957	-0.003	0.990	0.098	0.719	-0.334	0.176
Δ MCV	-0.322	0.166	0.035	0.882	-0.266	0.256	-0.194	0.412	-0.142	0.550	0.149	0.530	0.050	0.834	-0.077	0.748	-0.070	0.769	-0.212	0.369	-0.316	0.175	-0.082	0.732	0.032	0.894	-0.061	0.799	-0.056	0.837	-0.162	0.521
Δ MCH	-0.042	0.861	0.041	0.865	0.042	0.860	-0.361	0.118	-0.381	0.097	-0.041	0.864	-0.068	0.777	-0.276	0.239	-0.291	0.213	-0.395	0.084	-0.469	0.037	-0.266	0.257	-0.156	0.513	-0.251	0.286	-0.009	0.975	-0.099	0.695
Δ MCHC	0.270	0.249	-0.136	0.568	0.241	0.307	-0.232	0.326	-0.244	0.299	-0.146	0.539	-0.138	0.563	-0.257	0.274	-0.280	0.233	-0.217	0.357	-0.355	0.125	-0.241	0.305	-0.184	0.436	-0.271	0.248	-0.236	0.378	-0.072	0.776
Δ RDW	-0.297	0.204	0.176	0.459	0.124	0.601	-0.358	0.121	-0.292	0.212	-0.185	0.434	-0.331	0.154	-0.203	0.391	-0.265	0.258	-0.143	0.547	-0.492	0.028	-0.160	0.500	-0.103	0.666	-0.184	0.436	-0.108	0.690	-0.536	0.022
Δ Platelet	0.321	0.168	0.039	0.870	0.013	0.957	-0.313	0.179	-0.290	0.214	-0.264	0.260	-0.233	0.323	-0.105	0.661	-0.123	0.606	0.014	0.955	-0.172	0.468	-0.035	0.882	-0.055	0.818	-0.076	0.750	-0.142	0.599	-0.134	0.595
Δ Neutrophils	0.283	0.226	-0.038	0.875	-0.092	0.700	-0.386	0.092	-0.202	0.394	-0.069	0.772	-0.156	0.511	-0.373	0.106	-0.299	0.200	-0.031	0.898	-0.041	0.864	-0.273	0.244	-0.315	0.177	-0.329	0.157	0.035	0.899	-0.238	0.341
Δ Lymphocytes	0.262	0.264	0.357	0.122	0.307	0.189	-0.179	0.450	0.050	0.835	0.018	0.940	-0.045	0.849	-0.001	0.997	0.026	0.914	-0.085	0.721	-0.201	0.394	0.103	0.667	0.032	0.893	0.052	0.829	0.121	0.656	-0.095	0.709
Δ Monocytes	0.337	0.146	-0.085	0.722	0.093	0.696	-0.273	0.244	-0.030	0.900	0.166	0.483	0.110	0.645	-0.172	0.467	-0.102	0.668	-0.173	0.466	-0.314	0.178	-0.129	0.589	-0.120	0.614	-0.172	0.467	-0.097	0.720	-0.091	0.719
Δ Eosinophilic	-0.169	0.476	0.089	0.710	0.034	0.886	-0.026	0.912	0.104	0.663	0.379	0.099	0.288	0.219	-0.221	0.348	-0.173	0.467	-0.105	0.660	-0.307	0.188	-0.208	0.379	-0.229	0.331	-0.182	0.443	0.136	0.615	0.263	0.292
Δ Basophil	0.108	0.651	0.024	0.921	-0.227	0.335	0.150	0.527	0.105	0.659	-0.271	0.248	-0.337	0.146	0.016	0.946	0.029	0.903	0.198	0.404	0.064	0.789	0.087	0.714	0.098	0.681	0.054	0.820	0.368	0.161	0.170	0.501
Urine test																																				
Δ pH	-0.573	0.010	0.342	0.151	0.141	0.565	-0.239	0.325	-0.205	0.399	0.198	0.417	0.151	0.538	-0.310	0.196	-0.292	0.226	0.028	0.910	-0.143	0.559	-0.370	0.119	-0.465	0.045	-0.363	0.127	-0.187	0.504	-0.234	0.365
Δ Protein	0.193	0.429	0.105	0.668	0.030	0.904	-0.053	0.830	0.112	0.648	0.279	0.248	0.254	0.293	-0.043	0.861	-0.012	0.962	0.279	0.247	0.331	0.166	-0.164	0.501	-0.213	0.382	-0.166	0.496	-0.259	0.351	-0.137	0.601
Δ Hb	-0.273	0.259	0.323	0.177	-0.149	0.544	-0.362	0.128	-0.257	0.288	-0.045	0.853	-0.151	0.538	-0.239	0.324	-0.259	0.285	-0.299	0.214	-0.179	0.464	-0.213	0.380	-0.226	0.352	-0.226	0.352	-0.069	0.808	-0.426	0.088
Δ Ketones	0.195	0.424	-0.189	0.438	0.482	0.036	0.064	0.795	0.002	0.993	0.089	0.717	0.132	0.591	-0.009	0.970	-0.095	0.700	-0.335	0.161	-0.215	0.377	0.011	0.965	0.086	0.725	0.039	0.873	0.219	0.432	0.314	0.220

Δ Urobilinogen	-0.151	0.537	-0.215	0.376	0.129	0.598	-0.258	0.286	-0.301	0.210	-0.264	0.276	-0.225	0.354	-0.367	0.122	-0.368	0.121	0.192	0.430	0.228	0.348	-0.348	0.145	-0.391	0.098	-0.370	0.119	0.133	0.637	-0.257	0.319
Δ Specific weight	-0.063	0.798	-0.077	0.753	0.023	0.925	-0.007	0.977	0.014	0.954	0.234	0.335	0.196	0.421	0.020	0.934	0.020	0.934	-0.257	0.288	-0.032	0.896	-0.124	0.612	-0.070	0.777	-0.114	0.643	-0.261	0.347	-0.108	0.680
Intestinal permeability test																																		
Δ Diuresis 6h	-0.183	0.439	0.563	0.010	0.050	0.833	-0.660	0.002	-0.493	0.027	0.123	0.607	0.079	0.742	-0.491	0.028	-0.467	0.038	-0.179	0.451	-0.115	0.629	-0.456	0.043	-0.580	0.007	-0.503	0.024	-0.497	0.050	-0.529	0.024
Δ Lactulose	0.024	0.920	0.220	0.352	-0.078	0.743	-0.277	0.238	-0.144	0.544	0.365	0.114	0.386	0.092	-0.384	0.095	-0.354	0.126	0.206	0.385	-0.054	0.822	-0.415	0.069	-0.484	0.031	-0.421	0.065	-0.246	0.359	-0.217	0.386
Δ Mannitol	-0.009	0.970	0.066	0.782	0.032	0.895	-0.301	0.198	-0.386	0.092	-0.316	0.175	-0.239	0.310	-0.339	0.144	-0.321	0.167	-0.096	0.688	0.398	0.082	-0.317	0.173	-0.441	0.051	-0.360	0.119	-0.160	0.555	-0.375	0.125
PREDIMED	0.014	0.954	-0.279	0.233	0.106	0.656	0.310	0.183	0.244	0.299	0.098	0.682	0.166	0.484	0.155	0.514	0.176	0.458	-0.311	0.182	-0.008	0.973	0.067	0.779	0.083	0.728	0.128	0.591	-0.245	0.361	0.264	0.289
Cardiovascular parameters																																		
SCORE2¤^	-0.136	0.580	0.001	0.997	0.088	0.720	-0.033	0.895	-0.091	0.713	-0.422	0.072	-0.489	0.034	-0.044	0.858	-0.068	0.782	-0.341	0.153	0.016	0.950	-0.039	0.872	-0.045	0.856	-0.090	0.713	-0.102	0.717	-0.073	0.781
CUORE§	-0.104	0.672	-0.025	0.920	0.252	0.299	-0.251	0.300	-0.226	0.353	-0.361	0.129	-0.441	0.059	-0.230	0.344	-0.252	0.298	-0.379	0.109	-0.064	0.796	-0.206	0.397	-0.223	0.359	-0.291	0.226	-0.278	0.315	-0.347	0.173
SBP	-0.337	0.146	0.141	0.553	-0.200	0.397	-0.549	0.012	-0.431	0.058	-0.223	0.345	-0.389	0.090	-0.554	0.011	-0.563	0.010	-0.052	0.829	-0.103	0.665	-0.567	0.009	-0.588	0.006	-0.659	0.002	-0.583	0.018	-0.732	0.001
DBP	-0.192	0.417	-0.018	0.939	-0.200	0.398	-0.335	0.149	-0.162	0.494	-0.267	0.255	-0.372	0.107	-0.318	0.172	-0.290	0.216	0.172	0.468	0.027	0.912	-0.269	0.252	-0.289	0.217	-0.358	0.122	-0.098	0.718	-0.404	0.096

Δ refers to difference between week 0 and week 9.

* Significance refers to the Spearman correlation test, indicated by the coefficient r_s.

¤ Computed from 19 subjects.

§ 10 year risk of cardiovascular events according to the Progetto estimator. SCORE2-OP (Older People) estimator was used for subjects >70 years. Values were adjusted for subjects with inflammatory arthritis. Probability is expressed as percentage of risk.

^ 10 year risk of cardiovascular events according to the ESC (European Society of Cardiology), SCORE2 (Systematic Coronary Risk Evaluation 2) estimator. Values were adjusted for subjects with inflammatory arthritis. Probability is expressed as percentage of risk.

The subsequent baseline variables were excluded from the analysis of the study group due to inadequate case number: elevated IL-1α, fibromyalgia, uveitis, inflammatory bowel disease, HLA-B27.

W0, week 0; DD, disease duration; DAPSA, disease activity index in psoriatic arthritis; DAS28-CRP, disease activity score on 28 joints with C reactive protein; PASI, Psoriasis Area Severity Index; BSA, Body Surface Area; BASDAI, Bath Ankylosing Spondylitis Disease Activity Index; ASDAS-CRP, Ankylosing Spondylitis Disease Activity Score – C Reactive Protein; VAS, Visual Analogue Scale; PtGA, patient global assessment; PGA, Physician Global Assessment;BMI, Body Mass Index; LEI, Leeds Enthesitis Index; SPARCC, Spondylarthritis Research Consortium of Canada; hsCRP, High Sensitivity C Reactive Protein; ESR, Erythrocyte Sedimentation Rate; TNFα, Tumor Necrosis Factor alpha; CUORE, cardiovascular unique offer reengineered; SCORE2, systematic coronary risk evaluation; SBP, systolic blood pressure; DBP, diastolic blood pressure; PREDIMED, PREvención con DIeta MEDiterránea; TJC, tender joint count; SJC, swollen joint count; HAQ, Health Assessment Questionnaire; CDAI, clinical disease activity index; SDAI, Simple Disease Activity Index; WPAI, Work Productivity and activity Impairment questionnaire; HDL, High Density Lipoprotein; LDL, Low Density Lipoprotein; ALT, alanine aminotransferase; AST, aspartate aminotransferase; GGT, gamma glutamyl transpeptidase; TSH, thyroid-stimulating hormone; WBC, white blood cells; RBC, red blood cells; Hb; hemoglobin; MCV, mean corpuscular volume; MCH, mean corpuscular hemoglobin; MCHC, mean corpuscular hemoglobin concentration; RDW, red cell distribution width.
