# Supplementary material for: Ketogenic diet improves disease activity and cardiovascular risk in psoriatic arthritis: A proof of concept study
Source: PLoS One. 2025 Apr 22;20(4):e0321140. doi: 10.1371/journal.pone.0321140 (PMC12013891; doi:10.1371/journal.pone.0321140)
Supplement: S26 Table — (PDF) [file pone.0321140.s026.pdf]

Table S26. Association between categorical variables at W0 and the modification of categorical variables (clinical, inflammatory biomarkers and cardiovascular parameters).

	**Gender**		**Smoke ever**		**Higher education**		**Employed**		**bDMARDs**		**Axial involvement**		**Comorbidities**		**Metabolic syndrome**		**Cardiovascular comorbidities**		**W0 elevated IL-1β**		**W0 elevated IL-6**		**W0 elevated fecal calprotectin**		**W0 MDA**		**W0 PASS**		**W0 physical activity°**	
	**1**	**0**	**1**	**0**	**1**	**0**	**1**	**0**	**1**	**0**	**1**	**0**	**1**	**0**	**1**	**0**	**1**	**0**	**1**	**0**	**1**	**0**	**1**	**0**	**1**	**0**	**1**	**0**	**1**	**0**
Physical activity improvement	1 (7.7)	1 (14.3)	1 (16.7)	1 (7.1)	1 (7.7)	1 (14.3)	2 (12.5)	0 (0)	1 (9.1)	1 (11.1)	1 (8.3)	1 (12.5)	0 (0)	2 (22.2)	2 (20)	0 (0)	0 (0)	2 (15.4)	1 (25)	1 (6.7)	0 (0)	2 (13.3)	0 (0)	2 (15.4)	1 (11.1)	1 (9.1)	1 (10)	1 (10)	0 (0)	2 (20)
PASS improvement	0 (0)	1 (14.3)	0 (0)	1 (7.1)	0 (0)	1 (14.3)	1 (6.3)	0 (0)	0 (0)	1 (11.1)	1 (8.3)	0 (0)	1 (9.1)	0 (0)	1 (10)	0 (0)	1 (14.3)	0 (0)	0 (0)	1 (6.7)	0 (0)	0 (0)	1 (14.3)	0 (0)	1 (11.1)	0 (0)	1 (10)	0 (0)	1 (10)	0 (0)
MDA improvement	1 (7.7)	1 (14.3)	0 (0)	2 (14.3)	1 (7.7)	1 (14.3)	1 (6.3)	1 (25)	0 (0)	2 (22.2)	2 (16.7)	0 (0)	2 (18.2)	0 (0)	2 (20)	0 (0)	1 (14.3)	1 (7.7)	0 (0)	2 (13.3)	0 (0)	1 (6.7)	2 (28.6)	0 (0)	2 (22.2)	0 (0)	2 (20)	0 (0)	1 (10)	1 (10)
CUORE class improvement §	1 (7.7)	2 (28.6)	1 (16.7)	2 (14.3)	1 (7.7)	2 (28.6)	3 (18.8)	0 (0)	2 (18.2)	1 (11.1)	2 (16.7)	1 (12.5)	1 (9.1)	2 (22.2)	3 (30)	0 (0)	1 (14.3)	2 (15.4)	1 (25)	2 (13.3)	0 (0)	3 (20)	0 (0)	3 (23.1)	1 (11.1)	2 (18.2)	1 (10)	2 (20)	1 (10)	2 (20)
SCORE2 class improvement ¤^	0 (0)	0 (0)	0 (0)	0 (0)	0 (0)	0 (0)	0 (0)	0 (0)	0 (0)	0 (0)	0 (0)	0 (0)	0 (0)	0 (0)	0 (0)	0 (0)	0 (0)	0 (0)	0 (0)	0 (0)	0 (0)	0 (0)	0 (0)	0 (0)	0 (0)	0 (0)	0 (0)	0 (0)	0 (0)	0 (0)
IL-1α improvement	0 (0)	1 (14.3)	0 (0)	1 (7.1)	0 (0)	1 (14.3)	0 (0)	1 (25)	0 (0)	1 (11.1)	1 (8.3)	0 (0)	1 (9.1)	0 (0)	0 (0)	0 (0)	0 (0)	1 (7.7)	0 (0)	0 (0)	0 (0)	1 (6.7)	0 (0)	1 (7.7)	0 (0)	1 (9.1)	0 (0)	1 (10)	1 (10)	0 (0)
IL-1β improvement	2 (15.4)	3 (42.9)	2 (33.3)	3 (21.4)	2 (15.4)	3 (42.9)	3 (18.8)	2 (50)	3 (27.3)	2 (22.2)	4 (33.3)	1 (12.5)	3 (27.3)	2 (22.2)	3 (30)	3 (30)	1 (14.3)	4 (30.8)	4 (100)	0 (0)	1 (25)	4 (26.7)	1 (14.3)	4 (30.8)	0 (0)	5 (45.5)	1 (10)	4 (40)	1 (10)	4 (40)
IL-6 improvement	1 (7.7)	1 (14.3)	0 (0)	2 (14.3)	1 (7.7)	1 (14.3)	2 (12.5)	0 (0)	0 (0)	2 (22.2)	1 (8.3)	1 (12.5)	1 (9.1)	1 (11.1)	1 (10)	1 (10)	1 (14.3)	1 (7.7)	0 (0)	2 (13.3)	1 (25)	0 (0)	2 (28.6)	0 (0)	1 (11.1)	1 (9.1)	1 (10)	1 (10)	1 (10)	1 (10)
Fecal calprotectin improvement	1 (7.7)	1 (14.3)	1 (16.7)	1 (7.1)	1 (7.7)	1 (14.3)	1 (6.3)	1 (25)	0 (0)	2 (22.2)	0 (0)	2 (25)	1 (9.1)	1 (11.1)	1 (10)	1 (10)	1 (14.3)	1 (7.7)	0 (0)	2 (13.3)	1 (25)	1 (6.7)	2 (28.6)	0 (0)	1 (11.1)	1 (9.1)	1 (10)	1 (10)	1 (10)	1 (10)

Gender “1” refers to male, “0” refers to female; for the other variables “1” refers to “yes”, “0” refers to “no”.
Improvement refers to difference between week 0 and week 9.
Data are reported as number and percentage.
Significant associations are indicated by green cells. Significance refers to the Chi square test.
° Weekly, according to the Food Frequency Questionnaire.
¤ Computed from 19 subjects.
§ 10 year risk of cardiovascular events according to the Progetto CUORE estimator. SCORE2-OP (Older People) estimator was used for subjects >70 years. Values were adjusted for subjects with inflammatory arthritis. Probability is expressed as percentage of risk.
^ 10 year risk of cardiovascular events according to the ESC (European Society of Cardiology), SCORE2 (Systematic Coronary Risk Evaluation 2) estimator. Values were adjusted for subjects with inflammatory arthritis. Probability is expressed as percentage of risk.
The subsequent baseline variables were excluded from the analysis of the study group due to inadequate case number: elevated IL-1α, fibromyalgia, uveitis, inflammatory bowel disease, HLA-B27.
W0, week 0; bDMARDs, biological disease-modifying antirheumatic drugs; IL, interleukin; MDA, Minimal Disease Activity; PASS, Patient Acceptable Symptom State; CUORE, cardiovascular unique offer reengineered; SCORE2, systematic coronary risk evaluation.
