# Supplementary material for: Ketogenic diet improves disease activity and cardiovascular risk in psoriatic arthritis: A proof of concept study
Source: PLoS One. 2025 Apr 22;20(4):e0321140. doi: 10.1371/journal.pone.0321140 (PMC12013891; doi:10.1371/journal.pone.0321140)
Supplement: S27 Table — (PDF) [file pone.0321140.s027.pdf]

Table S27. Association between continuous variables at W0 and the modification of categorical variables during the study.

		W0 weight	W0 BMI	W0 abdominal circumference	W0 DAPSA	W0 DAS28-CRP	W0 SPARCC	W0 BSA	W0 PASI	W0 LEI	W0 ASDAS-CRP	W0 BASDAI	W0 age	Dd arthritis	Dd psoriasis	W0 SBP	W0 DBP	W0 SCORE 2 *^	W0 CUORE§
IL-1α improvement	1	79 (79;79)	29.2 (29.2;29.2)	106 (106;106)	22.1 (22.1;22.1)	4.1 (4.1;4.1)	8 (8;8)	0 (0;0)	0 (0;0)	6 (6;6)	2.7 (2.7;2.7)	6.5 (6.5;6.5)	65 (65;65)	8.8 (8.8;8.8)	10.8 (10.8;10.8)	120 (120;120)	75 (75;75)	6.9 (6.9;6.9)	3.1 (3.1;3.1)
	0	91.7 (82.5;101.6)	31 (29.2;33)	106 (103.5;115.5)	11 (5.2;24.2)	2.5 (2.1;3.5)	1 (0;3.5)	0 (0;1)	0 (0;1.8)	0 (0;2)	1.4 (0.5;1.6)	3.3 (1.1;4.2)	55 (50;59)	8.7 (3.9;14.8)	25 (9.1;122.7)	140 (132.5;145)	90 (80;90)	7.3 (3.7;14.5)	3.9 (2.7;8.3)
IL-1β improvement	1	97.7 (91.7;105.5)	31.2 (30.8;34.3)	110 (106;115)	12.1 (11;22.1)	2.9 (2.7;4.1)	1 (0;6)	0 (0;1)	0 (0;0.5)	1 (0;4)	1.6 (1.5;2.7)	3.8 (3.3;6.5)	60 (59;65)	8.8 (5;11.5)	15.8 (11.5;25)	135 (130;150)	90 (80;90)	8 (6.9;15)	4 (3.1;17.1)
	0	89.2 (81.5;97)	29.8 (28.8;32.7)	105 (102.5;112)	8 (5.1;24.7)	2.4 (1.9;3.5)	2 (0;3.5)	0 (0;1)	0 (0;2.4)	0 (0;2)	0.7 (0.3;1.5)	1.7 (0.8;4.1)	52 (48.5;57.5)	8.7 (3.9;16.3)	41.5 (5.7;122.7)	140 (132.5;142.5)	85 (80;90)	6.4 (3.7;12.5)	3.6 (2.7;6.6)
IL-6 improvement	1	110.8 (92.1;129.4)	42.4 (36.1;48.7)	122 (110.5;133.5)	17.9 (11.6;24.3)	3.8 (2.9;4.8)	3 (1.5;4.5)	25 (12.5;37.5)	3.5 (1.8;5.3)	2 (1;3)	1.3 (0.9;1.7)	2.8 (2;3.7)	54.5 (52.8;56.3)	4.3 (3.8;4.8)	82.5 (62.4;102.6)	142.5 (138.8;146.3)	85 (82.5;87.5)	4.4 (3.3;5.5)	2.2 (1.6;2.8)
	0	91 (82.4;97.6)	30.9 (28.9;32.8)	106 (104.3;113.8)	11.5 (5.9;21.4)	2.6 (2.2;3.6)	1.5 (0;3.8)	0 (0;1)	0 (0;1.6)	0 (0;2)	1.5 (0.5;1.6)	3.3 (1.2;4.3)	55.5 (50;59.8)	10.1 (4.6;15.3)	20.3 (7.8;102.9)	137.5 (130;143.8)	87.5 (80;90)	8 (4.1;15)	4 (2.7;8.8)
Fecal calproctectin improvement	1	122.6 (109.9;135.3)	43.7 (38.1;49.4)	130.5 (123.3;137.8)	16.8 (9.9;23.7)	3.4 (2.2;4.6)	0 (0;0)	28 (17;39)	5.5 (4.8;6.3)	0 (0;0)	1.3 (0.8;1.7)	2.8 (1.9;3.6)	58 (54.5;61.5)	7.5 (5.4;9.7)	43 (42.7;43.4)	147.5 (141.3;153.8)	90 (85;95)	14.8 (8.5;21)	31.7 (16.3;47)
	0	89.8 (81.1;97.5)	30.3 (28.9;32.8)	106 (103.3;109.5)	11.5 (5.9;21.4)	2.6 (2.2;3.6)	2 (0;5.5)	0 (0;1)	0 (0;0.9)	0.5 (0;3.5)	1.5 (0.6;1.6)	3.3 (1.3;4.3)	55.5 (50;59)	8.8 (4.6;15.3)	20.3 (7.8;122.7)	137.5 (130;143.8)	87.5 (80;90)	6.9 (4.1;12.9)	3.7 (2.7;6.7)
Physical activity improvement	1	101.1 (98.9;103.3)	31.9 (30.7;33.1)	111.5 (109.8;113.3)	5.1 (2.6;7.7)	1.6 (1;2.2)	0.5 (0.3;0.8)	0.5 (0.3;0.8)	0.3 (0.1;0.4)	0.5 (0.3;0.8)	0.9 (0.5;1.2)	1.9 (1.2;2.6)	55 (52.5;57.5)	8.4 (6.7;10.1)	15.9 (11.4;20.5)	140 (135;145)	92.5 (91.3;93.8)	11.9 (8.9;15)	11.2 (8.2;14.1)
	0	89.8 (81.1;97.6)	30.9 (28.9;32.8)	106 (103.3;114.5)	12.1 (5.9;27.3)	2.7 (2.2;4)	2 (0;5.5)	0 (0;1)	0 (0;1.8)	0 (0;3.5)	1.5 (0.6;1.8)	3.4 (1.3;4.5)	55.5 (50.3;59)	8.8 (3.6;15.3)	31.6 (11;122.7)	137.5 (131.3;143.8)	85 (80;90)	6.9 (3.6;12.9)	3.4 (2.7;6.7)
PASS improvement	1	73.5 (73.5;73.5)	29.8 (29.8;29.8)	99 (99;99)	5.2 (5.2;5.2)	1.9 (1.9;1.9)	6 (6;6)	0 (0;0)	0 (0;0)	4 (4;4)	0.5 (0.5;0.5)	1.2 (1.2;1.2)	58 (58;58)	5.3 (5.3;5.3)	122.7 (122.7;122.7)	150 (150;150)	90 (90;90)	6.6 (6.6;6.6)	3.4 (3.4;3.4)
	0	91.7 (82.5;101.6)	31 (29;33)	106 (104.5;115.5)	12 (6.6;25.5)	2.7 (2.2;3.8)	1 (0;3.5)	0 (0;1)	0 (0;1.8)	0 (0;2)	1.5 (0.6;1.8)	3.3 (1.3;4.5)	55 (50;59.5)	8.8 (3.9;14.8)	21.7 (8.8;83.2)	135 (130;142.5)	85 (80;90)	7.4 (3.7;14.5)	3.9 (2.7;8.3)
MDA improvement	1	77 (75.3;78.8)	29.2 (28.8;29.5)	102 (100.5;103.5)	5.1 (5.1;5.2)	1.9 (1.9;1.9)	5 (4.5;5.5)	0 (0;0)	0 (0;0)	2 (1;3)	0.4 (0.3;0.4)	0.9 (0.8;1)	65 (61.5;68.5)	15.9 (10.6;21.2)	82.1 (61.8;102.4)	145 (142.5;147.5)	87.5 (86.3;88.8)	14.6 (10.6;18.5)	9.2 (6.3;12.1)
	0	93 (83.1;103.6)	31.1 (29.3;33)	106.5 (104.3;115.8)	12.1 (8;27.3)	2.8 (2.3;4)	0.5 (0;3)	0 (0;1)	0.2 (0;1.8)	0 (0;2)	1.5 (0.7;1.8)	3.4 (1.7;4.5)	54 (50;59)	8.8 (3.6;13.3)	20.3 (7.8;102.9)	135 (130;143.8)	87.5 (80;90)	6.9 (3.6;12.9)	3.7 (2.7;6.7)
CUORE class improvement §	1	96.7 (88.7;101.1)	32.9 (31.2;33.6)	108 (107.5;111.5)	10.2 (5.1;20.2)	2.7 (1.6;3.1)	1 (0.5;5.5)	0 (0;0.5)	0 (0;0.3)	1 (0.5;3.5)	1.5 (0.9;1.7)	3.3 (1.9;3.9)	57 (53.5;58.5)	5 (4.8;8.4)	6.8 (5.7;15.9)	140 (135;145)	90 (85;92.5)	6.2 (6;12.1)	6.2 (5.7;11.7)
	0	90.3 (82.2;97.7)	30.8 (28.8;32.5)	106 (103;116)	12 (5.2;22.1)	2.5 (2.2;4.1)	2 (0;4)	0 (0;1)	0 (0;1.8)	0 (0;2)	1.4 (0.5;1.6)	3.3 (1.2;4.5)	55 (50;59)	8.8 (3.3;15.8)	41.5 (11.5;122.7)	135 (130;145)	85 (80;90)	7.4 (3.5;13.4)	3.4 (2.6;7.2)
SCORE2 class improvement ¤^	1	n.a.	n.a.	n.a.	n.a.	n.a.	n.a.	n.a.	n.a.	n.a.	n.a.	n.a.	n.a.	n.a.	n.a.	n.a.	n.a.	n.a.	n.a.
	0	91 (81.8;99.7)	30.9 (29.1;33)	106 (103.8;115.3)	11.5 (5.2;23.8)	2.6 (2.1;3.7)	1.5 (0;4.5)	0 (0;1)	0 (0;1.8)	0 (0;2.5)	1.5 (0.5;1.7)	3.3 (1.1;4.5)	55.5 (50;59.3)	8.8 (4.2;14.3)	23.4 (9.8;122.7)	137.5 (130;145)	87.5 (80;90)	6.9 (3.8;14)	3.7 (2.7;7.8)

For categorical variables “1” refers to “yes”, “0” refers to “no”.
Improvement refers to difference between week 0 and week 9.
Data are reported as median and interquartile range.
Significant associations are indicated by green cells. Significance refers to the Kruskal-Wallis test.
¤ Computed from 19 subjects.
§ 10 year risk of cardiovascular events according to the Progetto CUORE estimator. SCORE2-OP (Older People) estimator was used for subjects >70 years. Values were adjusted for subjects with inflammatory arthritis. Probability is expressed as percentage of risk.
^ 10 year risk of cardiovascular events according to the ESC (European Society of Cardiology), SCORE2 (Systematic Coronary Risk Evaluation 2) estimator. Values were adjusted for subjects with inflammatory arthritis. Probability is expressed as percentage of risk.
The subsequent baseline variables were excluded from the analysis of the study group due to inadequate case number: elevated IL-1α, fibromyalgia, uveitis, inflammatory bowel disease, HLA-B27.
W0, week 0; BMI, Body Mass Index; DAPSA, disease activity index in psoriatic arthritis; DAS28-CRP, disease activity score on 28 joints with C reactive protein; SPARCC, Spondylarthritis Research Consortium of Canada; BSA, Body Surface Area; PASI, Psoriasis Area Severity Index; LEI, Leeds Enthesitis Index; ASDAS-CRP, Ankylosing Spondylitis Disease Activity Score – C Reactive Protein; BASDAI, Bath Ankylosing Spondylitis Disease Activity Index; Dd, disease duration; SBP, systolic blood pressure; DBP, diastolic blood pressure; CUORE, cardiovascular unique offer reengineered; SCORE2, systematic coronary risk evaluation; IL, interleukin; PASS, Patient Acceptable Symptom State; MDA, Minimal Disease Activity.
