# Supplementary material for: Ketogenic diet improves disease activity and cardiovascular risk in psoriatic arthritis: A proof of concept study
Source: PLoS One. 2025 Apr 22;20(4):e0321140. doi: 10.1371/journal.pone.0321140 (PMC12013891; doi:10.1371/journal.pone.0321140)
Supplement: S1 Figure — (PDF) [file pone.0321140.s028.pdf]

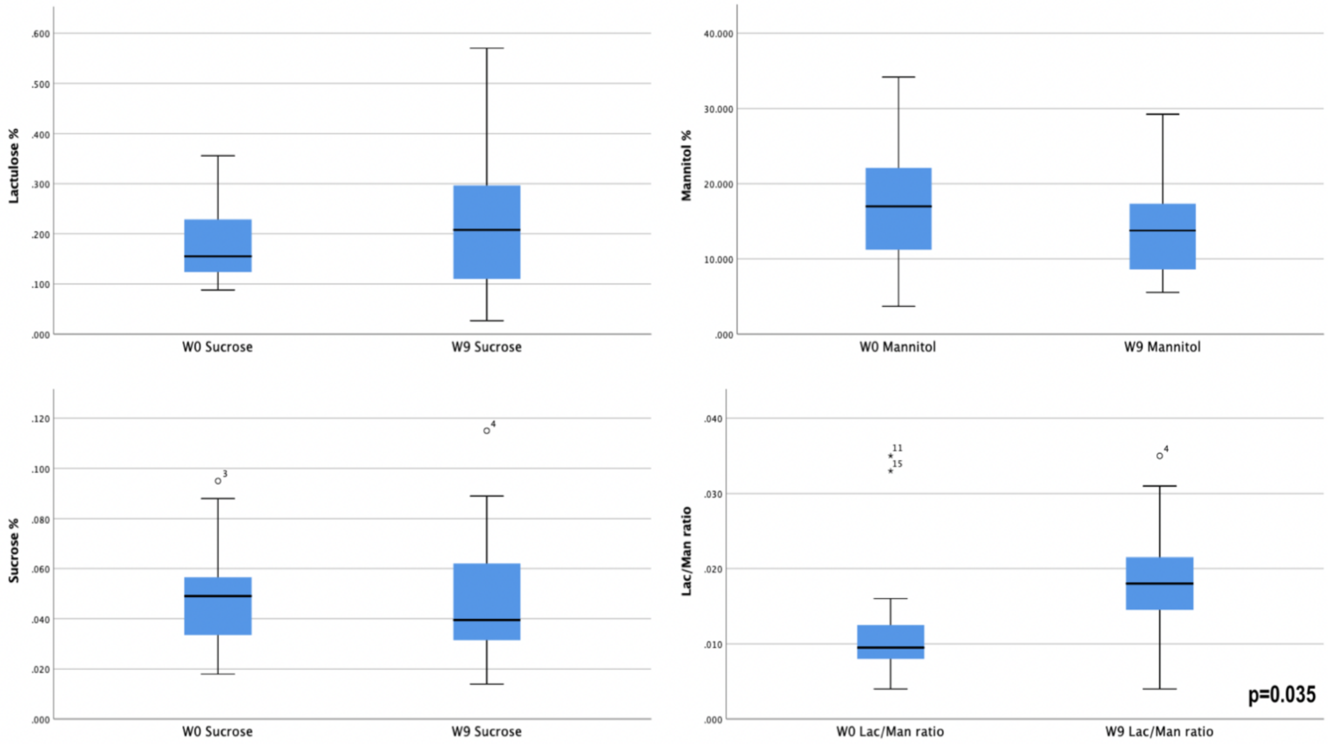

S1 Figure. Simple Boxplots of permeability tests during the study.
Significance refers to the tests of comparison between variables at W0 and W9, Wilcoxon test for continuous variables for paired data. Only significant p values are reported.
